# Specific patterns of PIWI-interacting small noncoding RNA expression in dysplastic liver nodules and hepatocellular carcinoma

**DOI:** 10.18632/oncotarget.10567

**Published:** 2016-07-13

**Authors:** Francesca Rizzo, Antonio Rinaldi, Giovanna Marchese, Elena Coviello, Assunta Sellitto, Angela Cordella, Giorgio Giurato, Giovanni Nassa, Maria Ravo, Roberta Tarallo, Luciano Milanesi, Anna Destro, Guido Torzilli, Massimo Roncalli, Luca Di Tommaso, Alessandro Weisz

**Affiliations:** ^1^ Laboratory of Molecular Medicine and Genomics, Department of Medicine, Surgery and Dentistry ‘Schola Medica Salernitana’, University of Salerno, Baronissi (SA), Italy; ^2^ Genomix4life, University of Salerno, Baronissi (SA), Italy; ^3^ Fondazione IRCCS SDN, Napoli, Italy; ^4^ Institute for Biomedical Technologies, National Research Council, Milano, Italy; ^5^ Pathology Unit, Humanitas Clinical and Research Center, Rozzano-Milan, Italy; ^6^ Department of Biomedical Sciences, Humanitas University, Rozzano-Milan, Italy; ^7^ Hepatobiliary and General Surgery Division, Humanitas Clinical and Research Center, Rozzano-Milan, Italy

**Keywords:** hepatocarcinogenesis, hepatocellular carcinoma, piRNAs, small non-coding RNA, smallRNA-seq

## Abstract

Hepatocellular carcinoma (HCC) is the result of a stepwise process, often beginning with development within a cirrhotic liver of premalignant lesions, morphologically characterized by low- (LGDN) and high-grade (HGDN) dysplastic nodules. PIWI-interacting RNAs (piRNAs) are small noncoding RNAs (sncRNAs), 23–35 nucleotide-long, exerting epigenetic and post-transcriptional regulation of gene expression. Recently the PIWI-piRNA pathway, best characterized in germline cells, has been identified also in somatic tissues, including stem and cancer cells, where it influences key cellular processes.

Small RNA sequencing was applied to search for liver piRNAs and to profile their expression patterns in cirrhotic nodules (CNs), LGDN, HGDN, early HCC and progressed HCC (pHCC), analyzing 55 samples (14 CN, 9 LGDN, 6 HGDN, 6 eHCC and 20 pHCC) from 17 patients, aiming at identifying possible relationships between these sncRNAs and liver carcinogenesis. We identified a 125 piRNA expression signature that characterize HCC from matched CNs, correlating also to microvascular invasion in HCC. Functional analysis of the predicted RNA targets of deregulated piRNAs indicates that these can target key signaling pathways involved in hepatocarcinogenesis and HCC progression, thereby affecting their activity. Interestingly, 24 piRNAs showed specific expression patterns in dysplastic nodules, respect to cirrhotic liver and/or pHCC.

The results demonstrate that the PIWI-piRNA pathway is active in human liver, where it represents a new player in the molecular events that characterize hepatocarcinogenesis, from early stages to pHCC. Furthermore, they suggest that piRNAs might be new disease biomarkers, useful for differential diagnosis of dysplastic and neoplastic liver lesions.

## INTRODUCTION

Hepatocellular carcinoma is the sixth most prevalent cancer and the third most frequent cause of cancer death [1]. In Europe, more than 90% hepatocellular carcinomas (HCCs) develop on a cirrhotic background, due to chronic hepatitis B or C infection, alcohol abuse or metabolic syndrome [2]. Human hepatocarcinogenesis is a multi-step process characterized by different nodular lesions, currently classified as low- (LGDN) and high-grade (HGDN) dysplastic nodules, early HCC (eHCC) and progressed HCC (pHCC), depending on the degree of cytological or architectural atypia [3]. The multistep nature of human hepatocarcinogenesis has long been suggested, and convincingly demonstrated by a recent molecular study showing a progressive increase in the rate of mutations of the telomerase reverse transcriptase (TERT) gene promoter from cirrhosis (no mutation) to LGDN (6%), HGDN (19%), eHCC (61%), small pHCC (42%) to advanced HCC (64%) [4]. Progressive overexpression of tumoral biomarkers in the sequence: dysplastic nodule-eHCC-pHCC further supports the multistep origin of liver cancer in cirrhosis [5]. Although many factors, including chromosomal anomalies, genetic and epigenetic alterations contribute to HCC onset and progression [6], the relevant molecular mechanisms remain largely unclear.

piRNAs comprise a large family of small (23–35 nucleotides [nt]), single stranded noncoding RNAs that bind to PIWI proteins, forming a piRNA-induced silencing complex (piRISC). PIWI proteins have been discovered in *D. melanogaster* germline tissues, but their presence has been recently reported also in mammalian somatic cells, including human cancers [7]. piRNAs can be grouped in four classes, according to their origin and function: 1) repeat-associated piRNAs, derived from intergenic loci (piRNA clusters), that are enriched in transposon fragments; 2) mRNA-derived piRNAs; 3) long noncoding RNA-derived piRNAs and 4) the worm specific 21U RNAs [8]. These sncRNAs are often transcribed as long (up to 200 kb), single-stranded primary precursors, processed in a Dicer-independent manner [9, 10] to mature piRNAs through still not fully understood mechanisms. Two main piRNA biogenesis pathways have been described in germline cells: the primary synthesis and the ‘ping-pong’ amplification mechanisms [11]. Mature piRNAs derived from piRNAs-precursors *via* primary processing, show a very strong preference for Uridine (U) at the 5′ end and no nucleotide bias at position 10. Those derived *via* secondary processing show, instead, a bias for Adenine (A) at position 10 and no 5′ end bias. Somatic cells do not use the latter amplification pathway and thus are likely to contain only primary piRNAs [12]. Furthermore, piRNA-likes have been identified and characterized by sequence analysis of expressed small RNAs in somatic tissues [13, 14], including rat liver [15].

The best-established biological role of piRNAs is inhibition of transposon mobilization by both epigenetic and post-transcriptional silencing [16, 17], but recent finding indicates their involvement in mRNA degradation in somatic cells [18, 19], acting in this case like microRNAs.

Given their regulatory role in control of genome stability and in epigenetic and post-transcriptional regulation of gene expression, it is not surprising that piRNAs has been found specifically expressed in several human neoplasms, including cervical [20], gastric [21–23], breast [24, 25], pancreatic [26], bladder [27] and endometrial [28] cancer and myeloma [29]. Recently Martinez et al. [30] demonstrated that a set of piRNAs is deregulated in many cancer types, and proposed that these might represent a core gene-set that facilitates cancer growth, while piRNAs unique to individual cancer types could contribute to cancer-specific biology. Notably, however, no information is available to date on piRNA expression in HCC and during liver carcinogenesis. Applying small RNA sequencing (smallRNA-Seq) we found that piRNAs are abundant in human liver, where the expression pattern of 125 of them clearly differentiates cirrhotic from HCC tissues. Interestingly, 24 piRNAs deregulated in advanced HCC show distinctive expression patterns also in earlier hepatic lesions, suggesting that these sncRNAs may participate to the carcinogenic process in this organ and could represent new markers of early hepatocarcinogenic lesions.

## RESULTS

### The piRNA expression pattern in liver distinguishes cirrhotic and tumor tissues

The PIWI subfamily of Argonaute proteins comprises four human members (PIWIL1/HIWI, PIWIL2/HILI, PIWIL3 and PIWIL4/HIWI2) [31], all initially found in testis. HIWI and HILI have been recently found highly expressed in a variety of human cancers [7], but little is known on their presence and expression in HCC. Therefore, we first investigated their expression in a cohort of 50 HCCs and matched normal liver tissue samples, available from The Cancer Genome Atlas (TCGA, http://cancergenome.nih.gov/). PIWIL1 and PIWIL2 mRNAs expression resulted altered in tumor tissues, confirming previous observations in HCC [32]. Furthermore, altered expression of several genes of the pathway, including DDX4, HENMT1, MAEL, PDL6, PRMT5, TDRD1, TDRD6, TDRD9, TDRKH and WDR77, suggesting that the piRNA pathway is functional in liver and its activity is modified in the liver cancer (Supplementary Figure S1).

**Figure 1:**
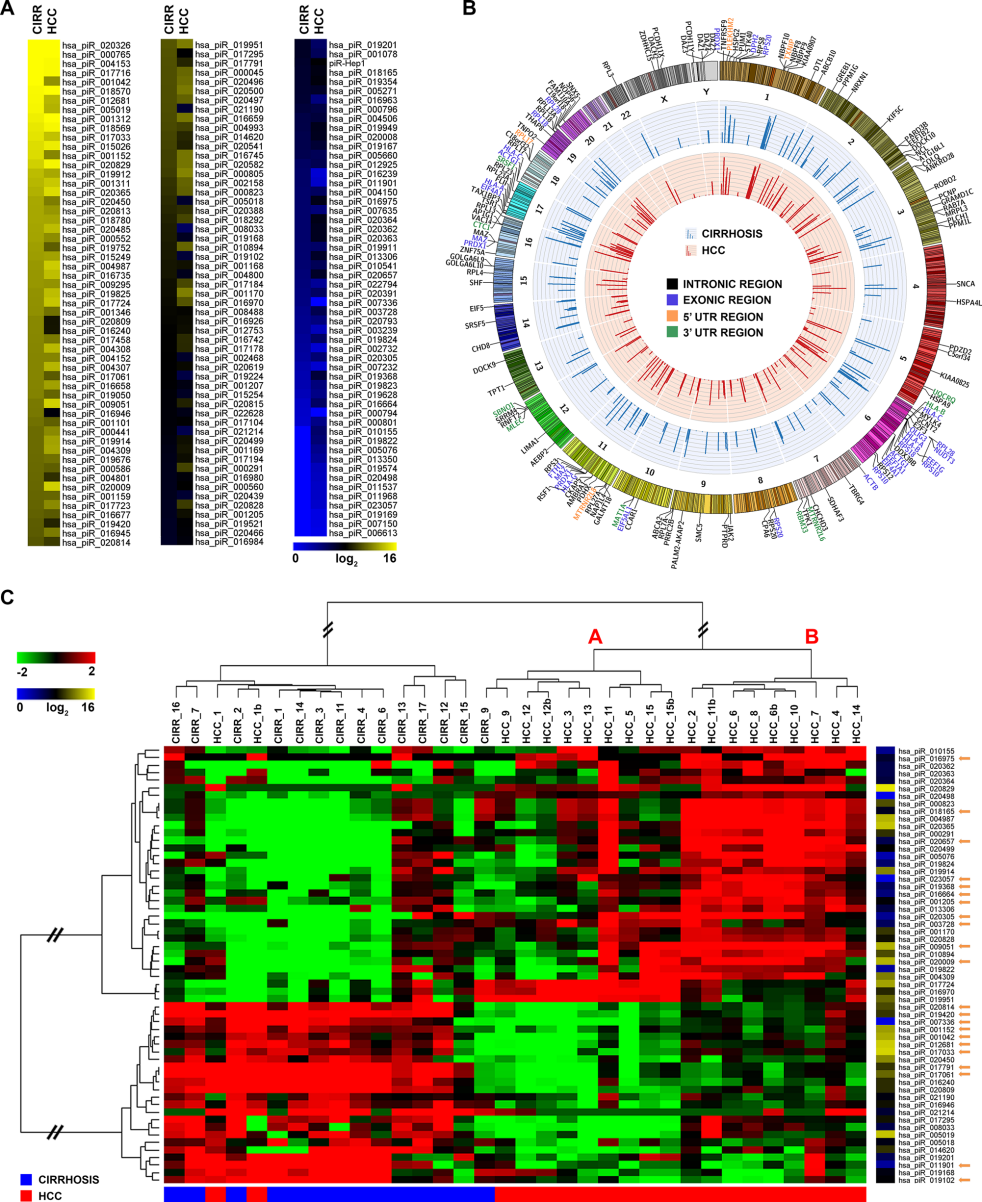
piRNA expression and genomic distribution in cirrhosis and HCC (**A**) Heatmap of piRNAs expression profile in human liver. (**B**) Catalogue of liver piRNAs identified. In the Circos plots, the outside ring shows the chromosome ideograms, with an annotation of the protein-coding transcripts in which each of them map. The middle and inner rings display average piRNA expression value in cirrhotic and HCC tissues, respectively. (**C**) Hierarchical clustering of piRNAs differentially expressed in matched cirrhotic *vs* HCC samples, identified with a non-parametric Wilcoxon Mann-Whitney test; log2(transformed RPM)-median centered expression (left) and average level of expression (right) are displayed for each RNA. Orange arrows mark piRNAs found deregulated in multiple cancers by Martinez et al. [30].

To identify and quantitate piRNAs, we then performed smallRNA-Seq of RNAs extracted from CN (*n* = 14) and HCC (*n* = 20) samples from 17 patients (Supplementary Table S1). Sequencing resulted in ~35 million reads/sample, with ~5% corresponding to known piRNAs (Supplementary Table S2), demonstrating that this class of small RNAs is indeed present in this tissue, as shown earlier in regenerating rat liver [15]. A total of 601 and 753 piRNAs were identified in cirrhotic and HCC samples, respectively. Interestingly, 81% of the total piRNA repertoire in liver was identified with few sequence reads, to indicate that these RNAs are expressed at a very low level, similarly to what generally observed for other regulatory sncRNAs, in particular microRNAs. Filtering out cases with a very low read counts (see Supplementary Materials and Methods), 197 piRNAs were selected and used for further analysis (Figure 1A and Supplementary Table S3). Notably, 41 piRNAs displayed a very high level of expression in at least one sample type (average read count ≥ 5,000 rpm, Figure 1A and Supplementary Tables 3A) and constituted ~90% of total liver piRNome. Interestingly, these RNAs were found highly expressed also in normal human liver (TCGA datasets, data not shown).

Liver piRNAs resulted distributed among all chromosomes except for the 21, with a preference for chromosome 6, that comprise 107 mapped piRNA loci (Figure 1B, Supplementary Figure S2A). Many liver piRNAs have multiple origins in the genome, with a maximum of 28 different locations for a single piRNA (hsa_piR_018596). Unlike piRNAs previously described in germline, but consistent with what observed in other somatic tissues [15, 25, 30], less than 5% liver piRNAs mapped to known human piRNA clusters, a result suggesting that the mechanism(s) driving their expression in hepatocytes is distinct from that of germline cells. The majority of liver piRNAs derive from intragenic loci (64%), with 94 (48%) mapping to introns, 22 (11%) to CDS, 35 (18%) to 5′-UTRs and 44 (23%) to 3′-UTRs (Figure 1B, Supplementary Figure S2B and Supplementary Table S3B). Of note, a sizeable fraction (78 out of 197) of the liver piRNAs identified here derived from protein coding, snoRNA and long non-coding RNA (lncRNAs) genes and from pseudogenes.

A comparative analysis of piRNA expression between cirrhosis and HCC showed significant differences between the two conditions (Figure 1A, 1B). Applying a stringent statistical analysis to search for differences in piRNAs expression, in tumors respect to matched non-malignant tissues, we identified the signature of 58 piRNAs shown in Figure 1C, including 16 belonging to the group of 41 highly expressed ones (average read count ≥ 5,000 rpm). Hierarchical clustering revealed a clear segregation of the samples in two major clusters, separating almost completely all CNs from HCCs. All the piRNAs included in the signature resulted differentially expressed in cancerous respect to cirrhotic liver (Fold Change, |FC| ≥ 1.5, False Discovery Rate, FDR ≤ 0.05); considering the median read counts within each group of samples, 34 were found overexpressed and 24 underexpressed in HCC samples (Supplementary Table S4). The HCC clade (Figure 1C) includes two recognizable sub-clusters, characterized by different piRNA expression levels. Considering FC variations in the two individuals HCC groups, respect to the cirrhotic one, it is interesting to notice that: for overexpressed piRNAs, 30 out of 34 molecules showed higher expression in clade B compared to A, while for underexpressed piRNAs, 20 out of 24 showed a more pronounced down-regulation in group A compared to B (Supplementary Table S4). Considering the biological processes associated to mRNAs targeted by the differentially expressed piRNAs, microvascular invasion, a histologic feature of HCC aggressiveness, scored more frequently in clade B (5 cases out of 9) as compared to A (1 case out of 9) (*P* < 0.04), suggesting that piRNAs could be involved in control of angiogenic processes in liver cancers.

Interestingly, many piRNAs distinguishing malignant form non-malignant liver tissues have been previously described with a similar behavior also in other cancers, specifically in gastric cancer [22, 23], myeloma [29], breast cancer [25], renal carcinoma [33], endometrial cancer [28], and pancreatic cancer [26] (see details in Supplementary Table S5). Furthermore, 22 of these (marked by orange arrows in Figure 1C) are in common with piRNAs recently found deregulated in multiples cancer types [30]. When combined, these results suggest that the piRNA signature identified here highlight involvement of these sncRNAs in liver cancer biology.

### Identification of novel liver piRNA-likes deregulated during hepatocarcinogenesis

**Figure 2:**
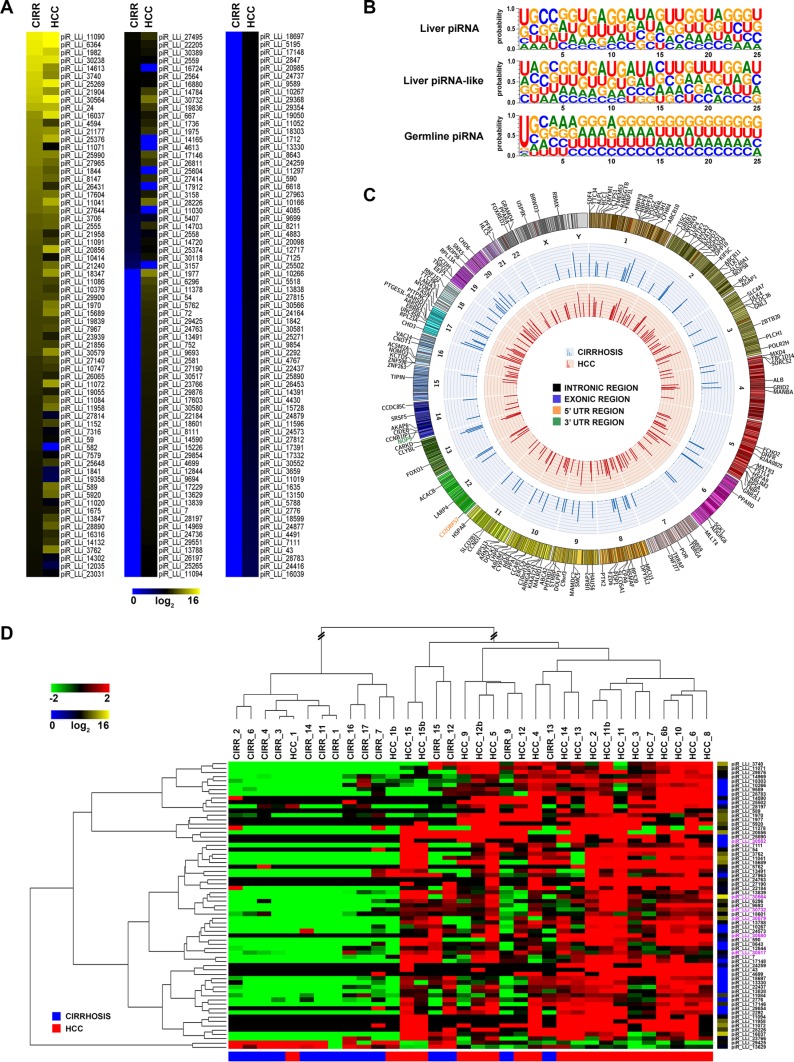
piRNAs-like RNA expression and genomic distribution in cirrhosis and HCC (**A**) Heatmap of piRNAs-like expression profile in human liver. Only piRNAs-like with median expression > 0 in at least one sample group are included. (**B**) Sequence logo showing nucleotide distribution in piRNAs and piRNA-likes expressed in liver, compared to that of germline piRNAs present in piRNAbank database. (**C**) Catalogue of human liver piRNA-like identified. In the Circos plots, the outside ring shows the chromosome ideograms, with an annotation of the protein-coding transcripts in which each of them map. The middle and inner rings display average piRNAs-like expression value of cirrhotic and HCC tissues, respectively. (**D**) Hierarchical clustering of piRNAs-likes differentially expressed in matched cirrhotic *vs* HCC samples, identified with a non-parametric Wilcoxon Mann-Whitney test; log2(transformed RPM)-median centered expression (left) and average level of expression (right) are displayed. Mitochondrial piRNA-likes are highlighted in pink.

SmallRNA-Seq in both cirrhotic and cancerous nodules revealed that ~25% of the reads aligning to the genome do not match to any annotated human RNA (Supplementary Table S2), a result in agreement with the notion that a sizeable fraction of existing RNAs is still uncharacterized. Previous works demonstrated that *in silico* prediction tools [13] allowed to identify new, non-annotated, piRNAs that were then experimentally validated and found to exert important biological activities [14]. Non annotated 21–35 nt long reads from all smallRNA-Seq datasets of CN and HCC samples, generated as described above and first analyzed for known piRNAs, were thus searched for novel piRNAs as described by Mei et al. [14]. Considering that, by definition, canonical piRNAs are PIWI protein-interacting RNAs and piRNAs present in piRNABank (http://pirnabank.ibab.ac.in/) were all originally discovered in germline cells due to their ability to bind PIWI proteins, the new somatic piRNAs discovered here were named piR_LLi (piRNA-Like in Liver), to avoid confusion. We identified 879 piR_LLis in CN and 646 in HCC samples. Likewise known liver piRNAs, several piR_LLis (71%) showed with very low read counts. Filtering for low copy molecules was thus applied and the top 359 piR_LLis were selected (Figure 2A and Supplementary Table S6) and further analyzed. These showed sequence features similar to those of known somatic piRNAs, but distinct from germline ones, including in particular a 5′-U bias and no preference for A in position 10 (Figure 2B), implying that their biogenesis occurs through the primary pathway described above. Considering their genomic location, piR_LLis show a very limited overlap with known germline piRNA clusters (< 5%), much like liver piRNAs, a preference for chromosomes 1 and 6, and, of note, 19 of them map to the mitochondrial chromosome (Supplementary Table S6B). Finally, piR_LLis originate preferentially from intragenic regions (53%) and predominately (99%) from the transcribed strand of introns (Figure 2C, Supplementary Figure S2B and Supplementary Table S6B)

piR_LLis showed a broad range of expression, with few of them expressed at very high levels (14 with > 10,000 rpm in at least one tissue type), and 108 exclusively expressed in HCCs (Figure 2A and Supplementary Table S6). To identified differences in piR_LLis expression between cirrhosis and HCC, we again applied the Wilcoxon Mann-Whitney test (|FC| ≥ 1.5, FDR ≤ 0.05), obtaining a 67 RNA signature of differently expressed piR_LLis (Figure 2D and Supplementary Table S7), including 6 of mitochondrial origin (highlighted in pink in Figure 2D). Hierarchical clustering revealed an almost complete segregation of the matched HCC-cirrhotic liver samples (Figure 2D). Notably, 65 piR_LLis resulted overexpressed and only 2 underexpressed in tumor respect to non-malignant tissues, suggesting a global increase in piRNA-like transcription in tumor tissues.

### *In silico* identification of HCC-responsive piRNA and piRNA-like target-RNAs

piRNAs were first shown to function in post-transcriptional regulation of transposon expression, inducing rapid and effective degradation of their transcripts [34], but they have been shown to form piRISC and induce mRNA deadenylation and decay *via* imperfect base-pairing in mouse elongating spermatids [18] and *Drosophila* embryo [19], inducing mRNA degradation by a mechanism that closely resembles that of microRNAs. To gain insight on the molecular processes in which piRNAs are involved in liver cancer cells, we searched for the mRNA-targets of known piRNAs and novel piR_LLis found differentially expressed in HCC respect to non-malignant tissues (Figure 1C and 2D), as described by Zhang et al. [35]. Results led to a set of 4.085 target-RNAs, including pseudogene transcripts and lncRNAs (Supplementary Figure S3A), some of which showing multiple binding sites for the same piRNA, while others binding up to 4 distinct piRNAs. Interestingly, the piRNA binding sites mapped to different regions of transcripts: 492 in 5′UTR, 612 in CDS and 2.981 in 3′UTR (Supplementary Figure S3A), similarly to what previously observed [15, 25, 28].

Ingenuity Pathway Analysis tool (IPA: www.ingenuity.com) was used to identify cellular pathways targeted by HCC-deregulated piRNAs. Results showed that the deregulated piRNAs identified here can target multiple signaling pathways, including death and TNF receptors, HIPPO, p53, PI3K/AKT, WNT/β-catenin, GADD45, AMPK, HMGB1 and PTEN pathways (Supplementary Figure S3B and Supplementary Table S8), that control, among others, cell cycle regulation, telomerase activity, protein ubiquitination, DNA methylation and apoptosis, all functions compromised in HCC. Indeed, the piRNA-targeted pathways affect such key processes as cell proliferation and death, angiogenesis, invasion, and metastasis, all known to be deregulated in HCC [3, 5, 6]. This was confirmed by downstream effects analysis (by IPA Molecule Activity Predictor tool), used to assess the overall effects (biological trends) of deregulated liver piRNAs on pathway activity. As an example, in Supplementary Figure S3C are summarized the predicted effects of the piRNAs targeting, respectively, the death receptor and PTEN pathways, that thereby influence cell proliferation (enhanced) and death (inhibited). These results support the possibility that the sncRNAs identified here represent a new class of regulators in liver cancer cells.

### piRNA and piRNA-like RNAs are deregulated during the early stages of hepatocarcinogenesis

**Figure 3:**
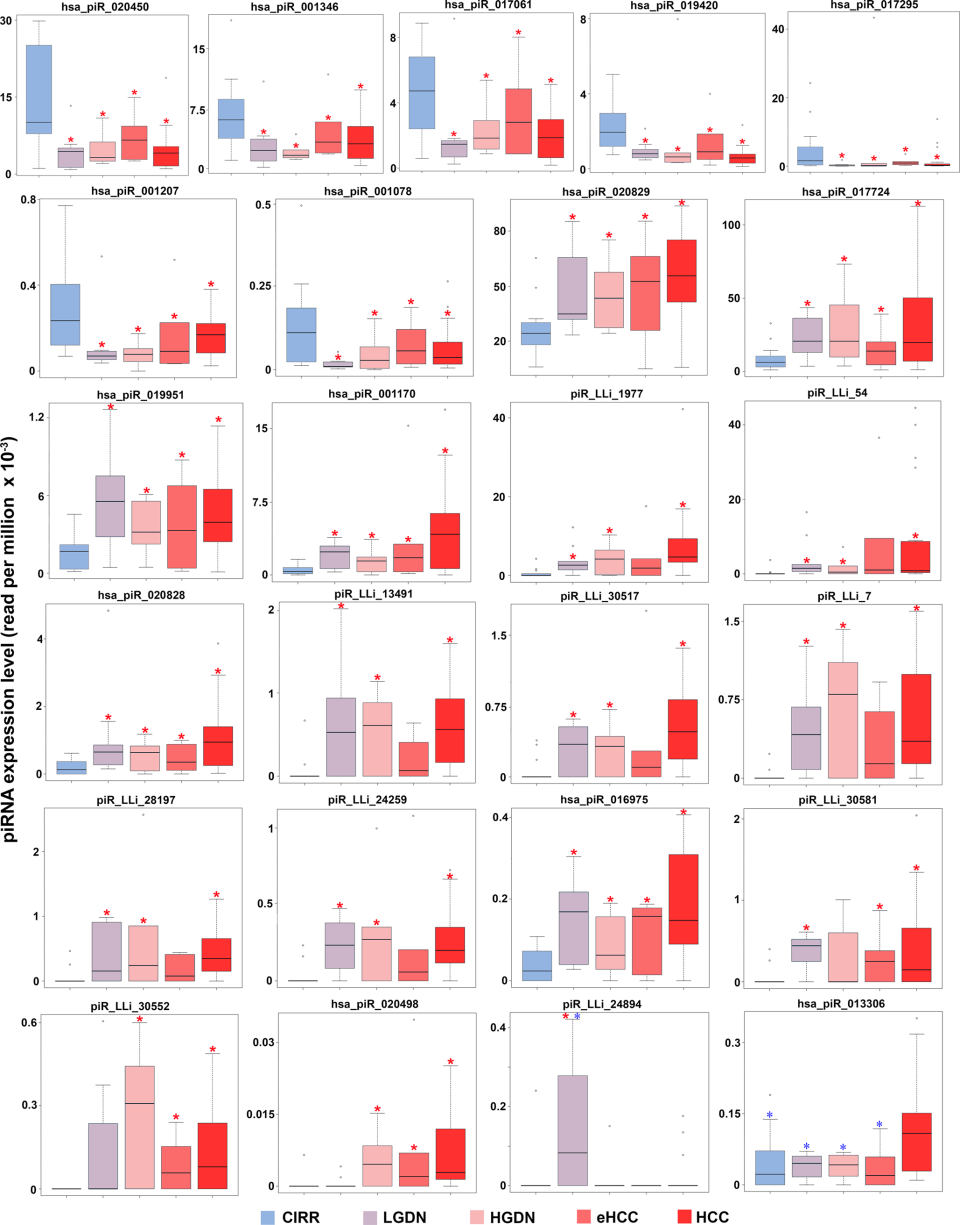
piRNA expression in early stages of hepatic carcinogenesis Boxplots summarizing differences in expression of piRNAs and piRNA-likes between cirrhotic (CIRR), LGDN, HGDN, eHCC and HCC samples, identified with a non-parametric Wilcoxon Mann-Whitnet test. Red and blue asterisks mark significant differences (*p* ≤ 0.05) respect to cirrhosis and HCC, respectively.

Human hepatocarcinogenesis is a long, stepwise process more often arising in a chronically altered hepatic microenvironment and proceeding from intermediate dysplastic lesions to eHCC and, ultimately, pHCC [36]. The sequential morphological lesions recognized during human hepatocarcinogenesis [37] are each characterized by distinctive clinicopathological features, with little molecular determinants known. A detailed knowledge of the early molecular changes occurring during liver carcinogenesis is thus much sought after, as it may provide means to help recognize the dysplastic nodules committed to malignant transformation, as well as early malignant lesions likely endowed with a more aggressive behavior. We thus performed smallRNA-Seq also in LGDN (*n* = 9), HGDN (*n* = 6) and eHCC (*n* = 6) samples (Supplementary Table S1), and investigated in detail piRNA and piR_LLi expression patterns by comparative analyses with respect to both CNs and pHCCs. This allowed identification of 15 piRNAs and 10 piR_LLis showing significant differences in expression among the groups of samples considered (Figure 3 and Supplementary Figure S4), providing clues of existing relationships with the carcinogenic process. Indeed, 7 piRNAs showed higher expression in CNs, that decreased significantly already in LGDNs and remained low across all the other pathological stages analyzed, up to pHCC. In contrast, 6 piRNAs and 8 piR_LLi displayed an opposite behavior, with very low or absent expression in cirrhotic liver and significant increases at all stages of malignant transformation. On the other hand, hsa_piR_020498 and piR_LLi_30552 were overexpressed from HGDN to progressed HCC, being almost undetectable in CN and LGDN, suggesting that they are involved only in later stages of the process. Finally, piR_LLi_24894 was detected only in LGDNs, while hsa_piR_013306 resulted up-regulated only in HCCs (Figure 3 and Supplementary Figure S4). These last results suggest that some piRNAs might be involved in stage-specific processes. When combined, these evidences indicate that dysplastic lesions are characterized by selective piRNA deregulation, which distinguishes them from cirrhotic nodules and, more often, is maintained up to pHCC.

## DISCUSSION

Understanding the molecular events leading to malignant transformation is required to unravel the genetic path of liver carcinogenesis, a necessary prerequisite to identify early diagnostic and prognostic markers and novel therapeutic targets. In recent years, several studies focused on the role of noncoding RNAs in hepatocarcinogenesis, given the pivotal role of these molecules in establishment of complex physiological and pathological phenotypes, including cancer [review in 38]. Such studies provided valuable knowledge on microRNA and lncRNA involvement in liver carcinogenesis. In this study, we investigated in detail the behavior of a new family of regulatory RNAs, piRNAs, in 14 cirrhotic and 20 matched HCC samples and thereby identified > 700 known piRNAs and 900 novel piRNA-likes expressed in human liver, indicating that PIWI-piRNAs system is active in benign and neoplastic tissues of this organ. The piRNAs identified in liver show a strong preference for Uridine at the 5th position and lack any Adenine bias at nucleotide 10, suggesting that their synthesis is likely to occur through the primary piRNA biosynthetic pathway, as previously shown in other somatic tissues (9, 15). Stringent analyses revealed a well-defined molecular signature, comprising 125 piRNAs, that discriminates HCCs from to cirrhotic liver (Figures 1C and 2D), providing a clear demonstration of piRNA deregulation in liver carcinogenesis. These results are supported by results obtained from a large TCGA cohort, demonstrating that many genes of piRNAs pathways are dys-regulated in HCC (Supplementary Figure S1). When combined, these results indicate presence of the piRNA pathway in human liver and its modulation during neoplastic transformation. This result is in line with those suggesting the potential of PIWI proteins as cancer diagnostic and prognostic markers [7], including a positive correlation between HIWI expression and liver tumor size and metastasis, matched by a negative one with survival rate [39–41]. Furthermore, MAEL, a key gene in the piRNA biogenesis pathways, has been recognized as oncogene for its role in HCC development and progression [42]. Finally, considering the known association between molecular events occurring during liver regeneration and neoplastic transformation [43–44], we recently demonstrated a significant reprogramming of the piRNome in an experimental model of liver regeneration post-hepatectomy, with proliferation-responsive piRNAs able to target key cellular processes implicated in organ regeneration [15]. By homology comparison between human and rat piRNAs, we found that 16 of the 72 piRNAs differentially expressed during rat liver regeneration are also expressed in human liver and 3 of them (hsa_piR_017724, hsa_piR_020829, hsa_piR_004309) are differentially expressed between CN and HCC in the present study, two of which (hsa_piR_017724 and hsa_piR_020829) are also deregulated already in dysplastic nodules, suggesting that these RNAs could be involved in hepatocyte proliferation. Altogether, these findings suggest that piRNAs represent new players in liver proliferation and carcinogenesis. In line with this possibility, prediction and functional analysis of the RNA targeted by piRNAs aberrantly expressed in HCC revealed a nonrandom, significant correlation with several major oncogenic pathways that are deregulated in HCC, including death receptor, Hippo, PI3K/AKT, Wnt/β-catenin, the p53 and PTEN pathways [3, 5, 6]. The piRNA targets include tumor suppressors, oncogenes, growth factors, cell cycle regulators, effectors of apoptosis and angiogenesis and cell adhesion molecules involved in cell-cell interaction (Supplementary Table S8), all known to take an active part in liver carcinogenesis and tumor progression. These results, although to be considered preliminary in the absence of rigorous experimental validation, support the possibility that the liver piRNAs discovered here target key cellular pathways in normal and transformed hepatocytes. In line with this view, the sncRNA expression patterns identified match to specific clinicopathological characteristics of HCC, since based on piRNA expression the tumor samples cluster in two different groups (Figure 2D), with group B comprising tumors characterized by a more advanced stage of the disease and increased angiogenic potential. This last process is known to be activated early during carcinogenesis and to be important for tumor growth and metastatic potential [45]. Interestingly, hsa_piR_00823, one of the piRNAs differentially up-regulated in HCCs (FC 1,9 and 5,7 for clade A and B, respectively: Figure 1C and Supplementary Table S4), has been involved in regulation of *de novo* DNA methylation and angiogenesis in multiple myeloma, where its inhibition reduced VEGF secretion by cancer cells [29].

**Figure 4:**
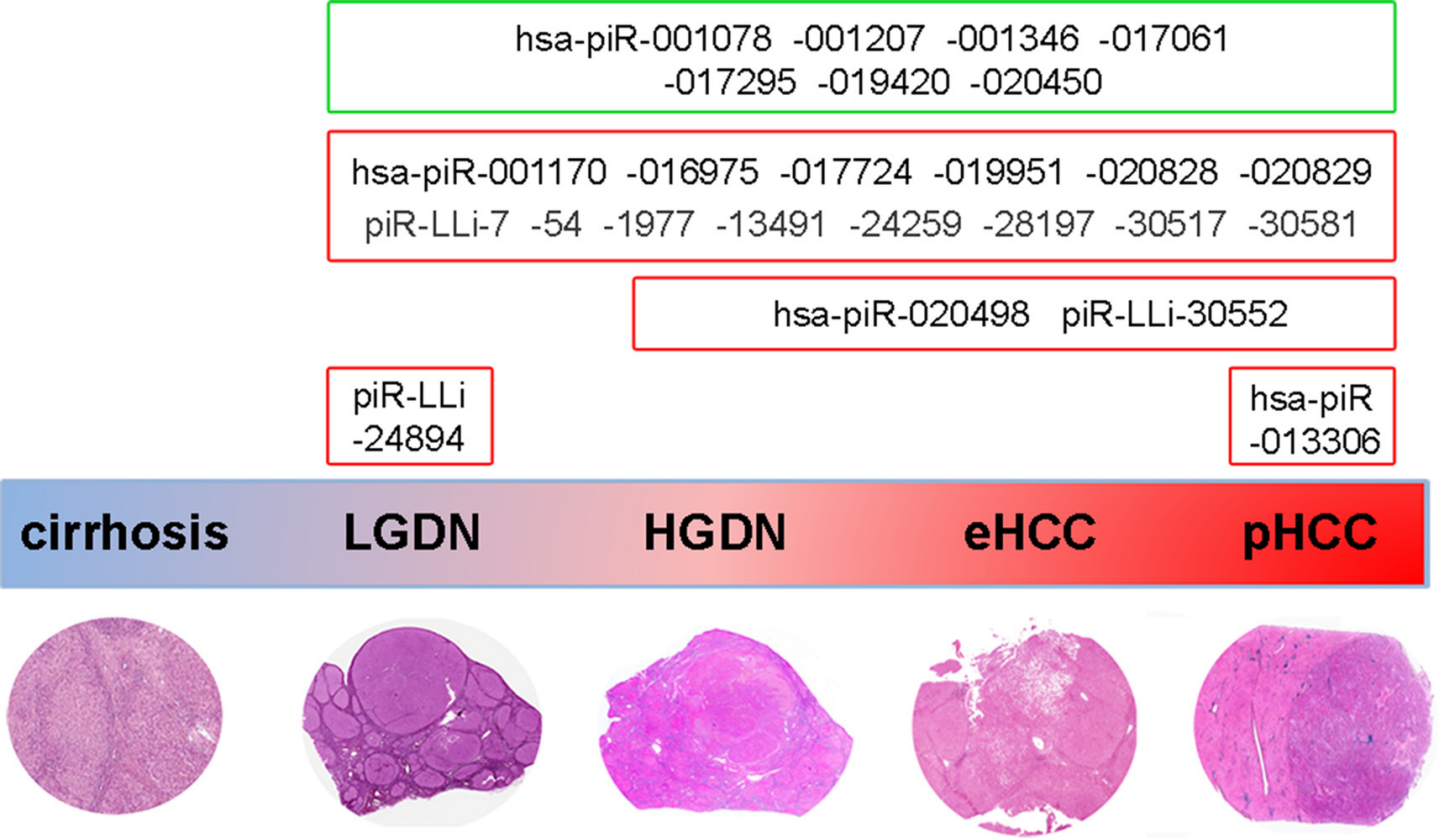
Overview of piRNA expression changes during human liver carcinogenesis The scheme summarizes the main results of piRNA and piRNA-like expression changes detected at different steps of human hepatocarcinogenesis. Deregulation of some of these molecules in dysplasia or HCC respect to cirrhosis supports the hypothesis that deregulation of these molecules may be implicated in tumor onset. A potential involvement of these small RNAs in malignant transformation and tumor progression is further suggested by specific changes in their expression confined to HGDN, eHCC and pHCC, or only in overt cancerous lesions. The green and red boxes indicate, respectively, piRNAs down- and up-regulated in HCC samples respect to paired non cancer tissues.

HCC is typically preceded by appearance of non-malignant liver nodules, that frequently contain one or more microscopic transformed foci, suggesting that dysplastic nodules, especially HGDNs, could be viewed as the earliest HCC precursors [46–48]. Due to the arduous histological distinction of pre-cancerous lesions from well-differentiated HCCs, we evaluated whether piRNAs were also differentially expressed in the different classes of nodules. To this end, we analyzed piRNA expression also a series of dysplastic nodules (9 LGDN, 6 HGDN) and eHCC (6) from the same patient series (Supplementary Table S1), and compared piRNome expression profiles both among these lesions and respect to cirrhotic and HCC nodules from the same patients. Results reveal how specific piRNA expression patterns mark known stages of the carcinogenic pathway (summarized in Figure 4): while high piR_LLi_24894 is a feature of low-grade lesions only, significant accumulation of piR_LLi_30552 and hsa_piR_020498 occurs from HGDNs up to pHCCs (Figure 4 and Supplementary Figure S4), and hsa_piR_013306 accumulates only in HCC. On the other hand, most of the changes found in HCC occur already in LGDNs, the earliest known stage of hepatocarcinogenesis investigated here. When combined, all these results strongly support the possibility that piRNAs are directly or indirectly involved in the spectrum of changes that characterize the hepatic carcinogenic process.

Comparing the results obtained here in HCC with previous work in other cancers, we observed that several piRNAs are similarly affected by cell transformation. In particular, 31 out of the 58 piRNAs of the HCC signature of Figure 1C were found aberrantly expressed in a comparable way in other cancer types, when malignant tissues were compared to non-malignant ones [22, 25, 26, 28–30, 33], suggesting that a set of these sncRNAs may be involved in common steps of the carcinogenic pathway, independently of the cell of origin. Indeed, Martinez et al. [30] reported that the piRNome exhibits specific pan-cancer, as well as tumor-type specific, expression patterns.

It is noteworthy to mention that a very high fraction (~80%) of the liver piRNA repertoire identified here is expressed at a very low level, so that these RNAs are likely to be present, on average, in less than one molecule/cell. We cannot exclude that they exert biological functions, as each of them could be expressed in only one or few cell subtypes among those present in the tissues analyzed, that comprise inflammatory and immune cells, stroma, vasculature and, of note, cancer stem cells. The molecular resolution of the analytical methods applied here (whole tissue analysis) is not sufficient to investigate this aspect, but the technological advances of single cell transcriptome sequencing [49] made now possible to address it. In this respect, it is worth mentioning that piRNAs are known to be involved in stem cell regulation, and PIWI proteins are expressed in normal and cancer stem cells.

In conclusion, we identified a piRNA expression signature specific of progressed liver cancers that distinguishes it from non-malignant liver. This finding, combined with the observation of a progressive deregulation of the liver piRNome during carcinogenesis, suggests that members of this novel family of small regulatory RNAs are likely to play a role in malignant transformation of hepatocytes. Collectively, these results indicate that piRNAs represent a new family of regulatory and effector molecules involved in liver carcinogenesis, whose better understanding will help shed light in HCC pathogenesis and is exploitable to better characterize dysplastic and neoplastic liver lesions.

## MATERIALS AND METHODS

### Samples collection

Resected specimens from 17 patients, each with multiple hepatocellular nodules (HN) well representative of different steps of human hepatocarcinogenesis, were included in this study. After proper identification of the hepatocellular nodule on the H/E section, the lesion was manually microdissected from sequential, matched 10 μm paraffin-embedded sections. These tissue samples harbored 61 HN (mean: 3,5 HN/patient; range 2-6 patient) as follows: 17 cirrhotic nodules (CN), 9 LGDN, 6 HGDN, 6 eHCC, and 23 pHCC. Clinical and pathological features of the series are illustrated in Supplementary Table S1. Of these 61 nodules, 55 had sufficient material for a complete morphological characterization and molecular analysis, namely 14 CN, 9 LGDN, 6 HGDN, 6 eHCC and 20 pHCC.

### RNA purification and small RNA sequencing

Total RNA was extracted from FFPE sections of human livers using RNeasy FFPE Kit (QIAGEN GmbH, Hilden, Germany) in duplicates and quantitated with NanoDrop-1000 spectrophotometer (Thermo Fisher Scientific, Cinisello Balsamo, Italy). For smallRNA-seq, 1μg of total RNA/samples was used for library preparation with Illumina TruSeq smallRNA sample preparation Kit and each library (8pM) was sequenced on HiSeq2500 (Illumina) for 50 cycles at Genomix4life (www.genomix4life.com). SmallRNA-Seq data were analyzed using iMir [50], see Supplementary Materials and Methods for bioinformatics analyses. Raw smallRNA-Seq data are available in ArrayExpress database (https://www.ebi.ac.uk/arrayexpress/) with accession: E-MTAB-3973.

### Bioinformatics analysis

The sequencing reads from each samples were processed using iMir [50] to detect piRNAs expression using piRNABank annotation [51] adding piR-HEP1 sequence [32]. Other non-coding RNA reads mapping were discarded in further analysis, briefly, human miRNA annotated in miRBase (v21) and other ncRNAs (rRNA, tRNA, snoRNA) annotated in UCSC were downloaded and integrated into a comprehensive dataset for annotated non-coding RNAs.

The reads failing to match known piRNAs and other sncRNAs, with lengths between 25 and 35 nt, were filtered using a piRNA prediction tool [13]. The retained reads were mapped to human genome (hg19) using Bowtie v0.12.9 [52] to infer the genomic coordinates, then BEDTools suite v2.23.0 [53] and custom python scripts were used to recognize the piRNA-LLi loci and to calculate the read coverage.

The expression values of piRNAs and piRNA-LLi were represented like read per million (RPM) values making them comparable across samples and to filter out low expression molecules *filtered.data* function of R package NOISeq [54] was used.

Differential expression analysis between different tissues were performed with R using Wilcoxon Mann-Whitney test (α < 0.05). piRNAs were considered differentially expressed when showing absolute Fold Change (FC) ≥ 1.5 and significance false discovery rate (FDR) ≤0.05.

In order to find putative mRNAs target, piRNAs and piRNA-LLi sequences were cut from second nucleotide with length twenty and mapped to reverse complement of RefSeq annotation (release 70) using Bowtie [52] with three miss-match like previous described in Zhang et al [35].

Unsupervised hierarchical clustering of samples based on expression profiles of selected piRNAs was performed using Ward's agglomeration method operated on Kendall rank correlation coefficient.

The functional analyses were generated with IPA (Ingenuity^®^ Systems, www.ingenuity.com) to identify the canonical pathways associated with putative mRNAs targeted by piRNAs. The Molecule Activity Predictor (MAP) tool in IPA was used to simulate the downstream effects of activation or inhibition of molecules in the pathway considering an inverted correlation between piRNAs and their direct putative targets.

## SUPPLEMENTARY MATERIALS
















