# Supplementary material for: Specific patterns of PIWI-interacting small noncoding RNA expression in dysplastic liver nodules and hepatocellular carcinoma
Source: Oncotarget. 2016 Jul 13;7(34):54650–61. doi: 10.18632/oncotarget.10567 (PMC5342370; doi:10.18632/oncotarget.10567)
Supplement: Supplementary file 1 [file oncotarget-07-54650-s001.pdf]

# Specific patterns of PIWI-interacting small noncoding RNA expression in dysplastic liver nodules and hepatocellular carcinoma

## Supplementary Materials

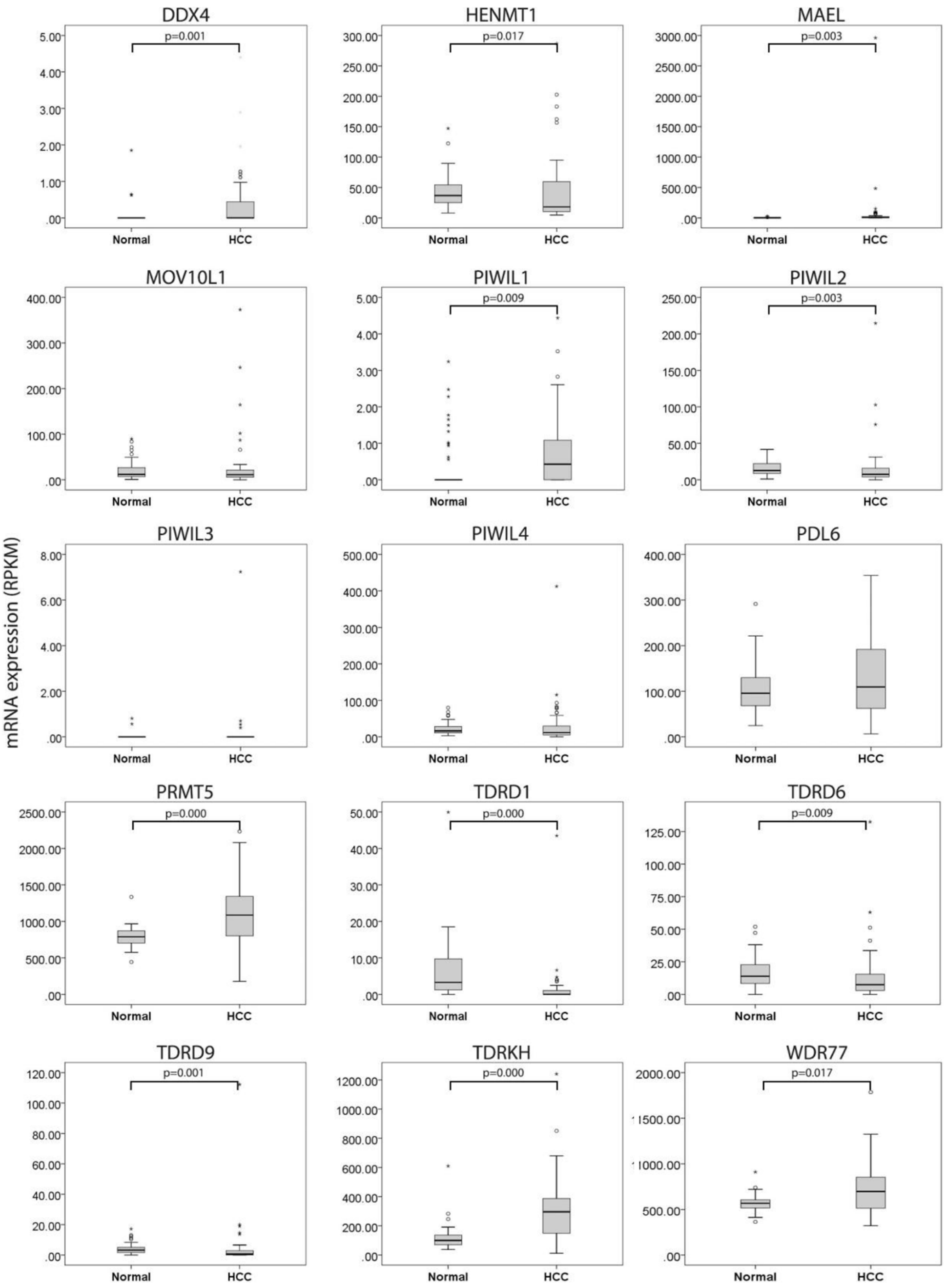


**Supplementary Figure S1: Presence of the piRNA–PIWI pathway in human liver.** Expression of transcripts encoding 12 known components of the piRNA biogenesis pathway in 50 matched non cancerous liver and HCC (data downloaded from TCGA, http://cancergenome.nih.gov/). Mann–Whitney *U* test was used in statistical comparisons and reported only when significant.

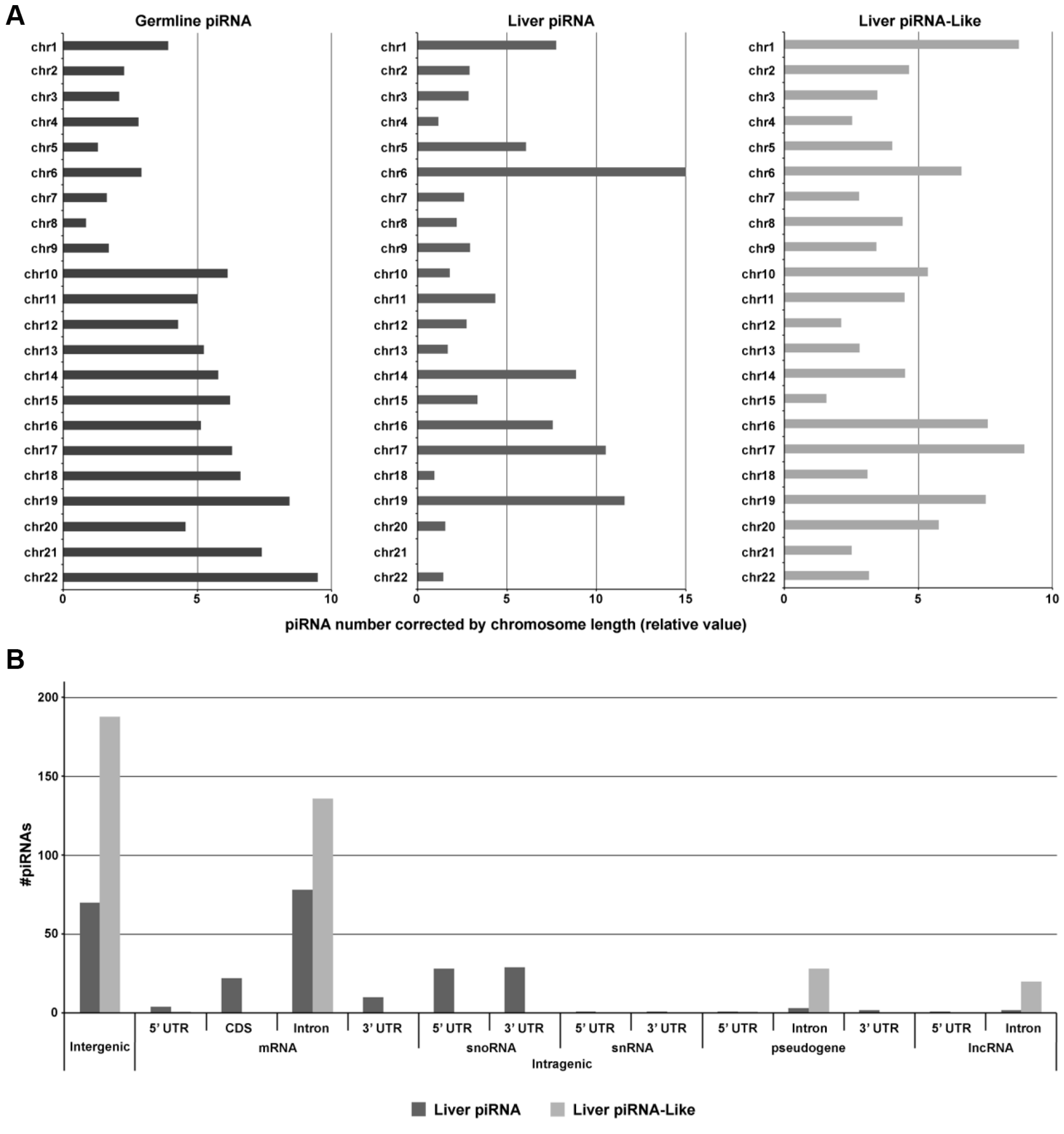


**Supplementary Figure S2: Chromosomal and genomic distribution of piRNAs and piRNAs–like.** (**A**) Bar plots of germline piRNA (piRNABank), liver piRNAs and liver piRNAs–like loci distribution between chromosomes. (**B**) Bar plot showing genomic annotation of expressed piRNAs and piRNA–likes, considering only piRNAs originating from the transcribed strand of genomic loci.

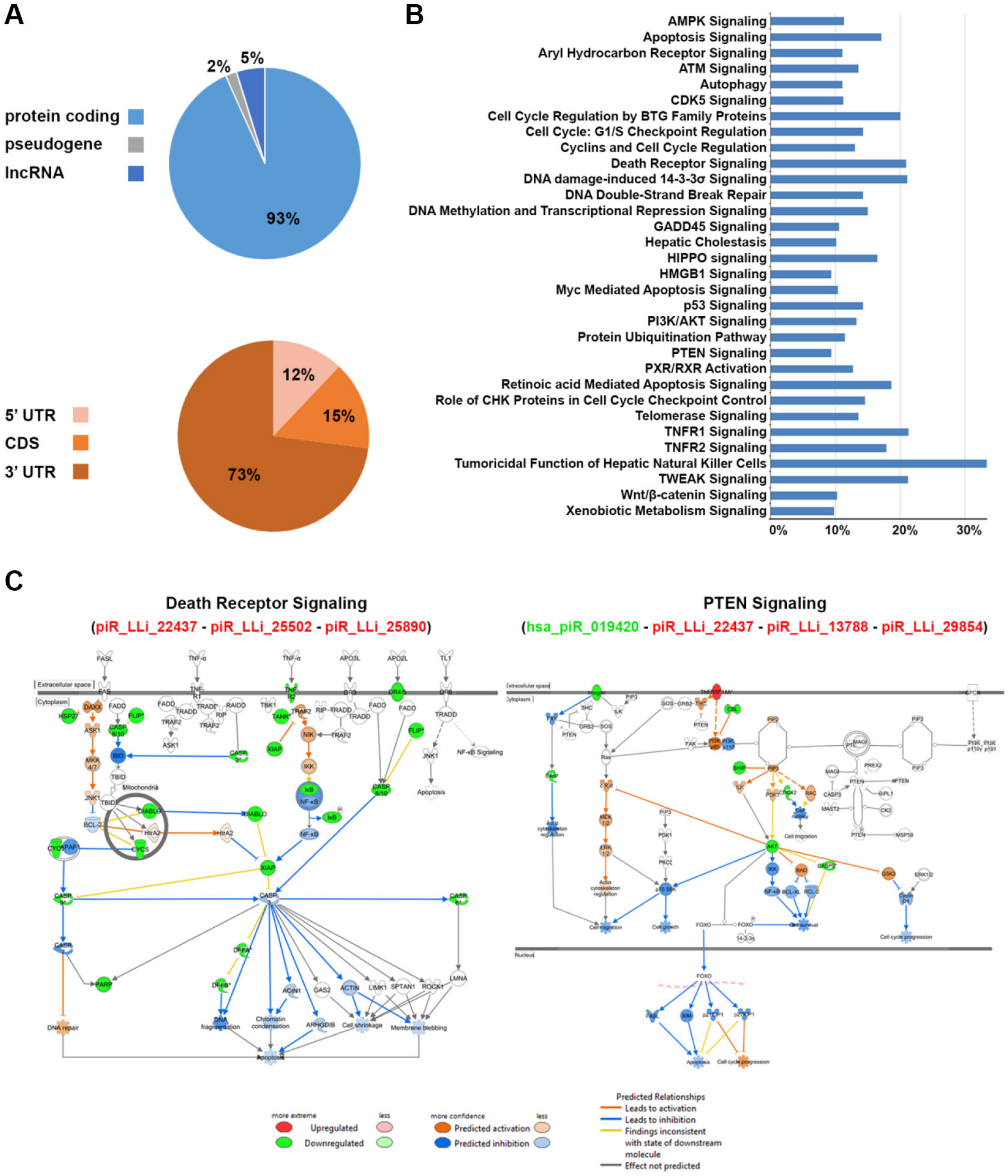


**Supplementary Figure S3: piRNA–targeted functional pathways.** (**A**) Pie charts representing the piRNA binding sites distribution in different transcripts type and transcripts regions. (**B**) The diagram shows the pathways significantly affected in HCC by the deregulated piRNAs identified here. Horizontal bars indicate the percentage of piRNA–targeted genes within each pathway. (**C**) IPA Molecule Activity Predictor analysis performed on death receptor (A) and PTEN signaling. The piRNAs regulating the pathways are reported, in each case, above the diagrams in red (up–regulated) or green (down–regulated), according to their behavior in HCCs. The expression of putative piRNA target–mRNAs have been considered only when their expression inversely correlated to that of the targeting piRNAs. The relationship between nodes (edges between pathway components) and the node themselves (pathway components) are color–coded based on the predicted relationships.

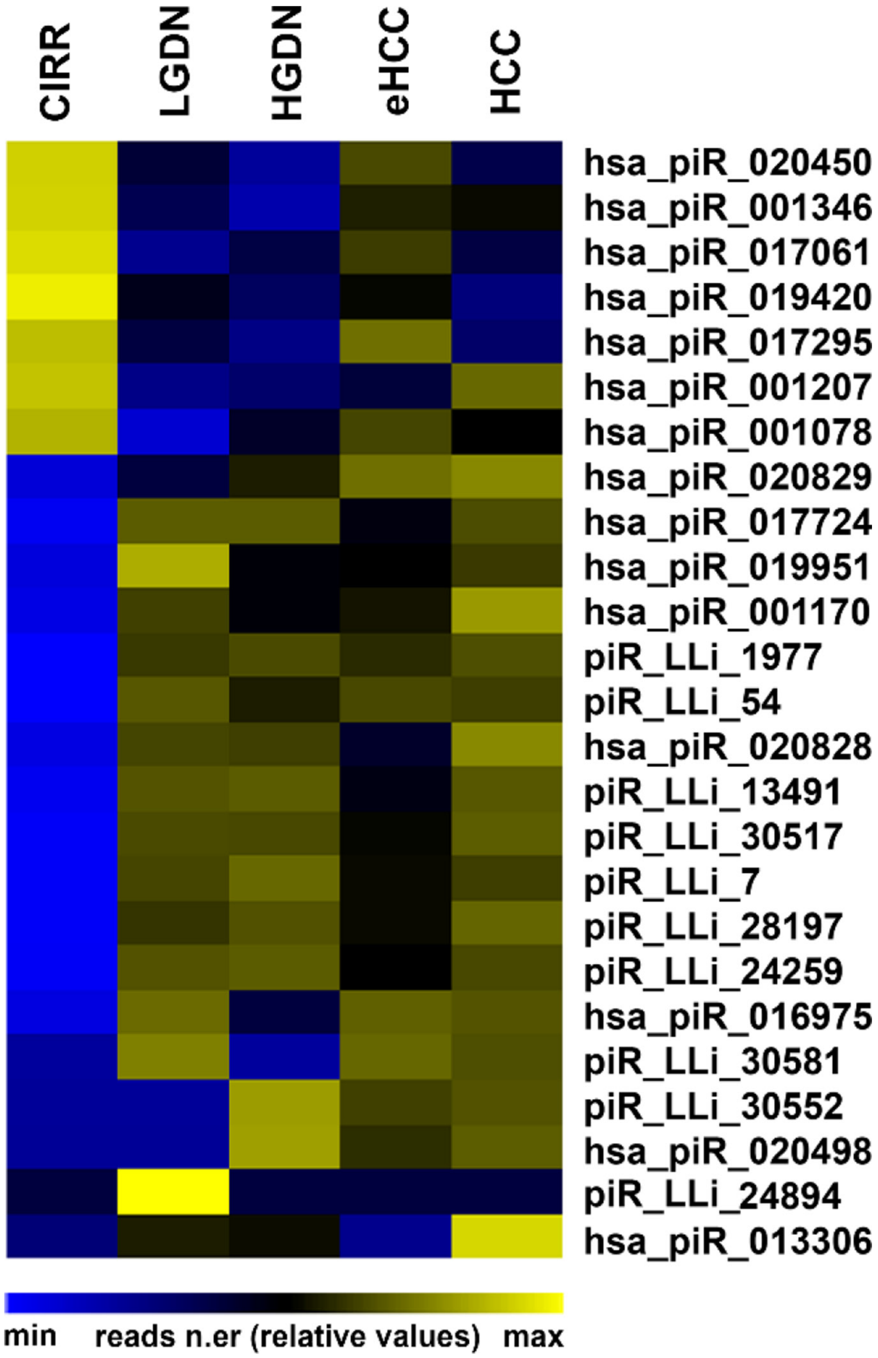


**Supplementary Figure S4: piRNAs expression profile during hepatocarcinogenesis.** Heatmap of piRNAs and piRNA–likes expression profile in cirrhotic tissue, dysplastic nodules (LGDN and HGND), eHCC and progressed HCC. Expression intensities are displayed from blue (low expression) to yellow (high expression).

## Supplementary Table S1: Clinical and pathological features of the series analyzed

| | Gender/ age | Aetiology | Type and number of hepatocellular nodules CN:LGDN:HGDN: eHCC:pHCC | HCC features | Surrounding liver | DFS | OS |
|---|---|---|---|---|---|---|---|
| Patient 1 | M, 77 | HCV | 1:0:0:0:2 | pT2, G2, V0, R0 | cirrhosis | 68 | 72 (*ad*) |
| Patient 2 | F, 75 | HCV | 1:0:0:0:1 | pT2, G2, V1, R0 | cirrhosis | 16 | 38 (*dod*) |
| Patient 3 | M, 67 | NASH | 1:1:0:0:1 | pT1, G1, V0, R0 | cirrhosis | 7 | 72 (*dod*) |
| Patient 4 | F, 65 | HCV | 1:0:0:1:1 | pT2, G2, V0, R1 | severe hepatitis | 34 | 96 (*ad*) |
| Patient 5 | M, 47 | HCV | 1:1:0:1:1 | pT2, G4, V0, R0 | cirrhosis | 48 | 48 (*and*) |
| Patient 6 | M, 65 | HBV | 1:0:0:0:3 | pT2, G2, V1, R1 | cirrhosis | 24 | 68 (*ad*) |
| Patient 7 | M, 69 | *uk* | 1:0:0:1:1 | pT3a, G2, V1, R0 | cirrhosis | 7 | 14 (*ad*) |
| Patient 8 | M, 61 | Alcohol | 1:1:0:0:1 | pT2, G1, V1, R1 | cirrhosis | 60 | 60 (*and*) |
| Patient 9 | M, 64 | Alcohol | 1:0:1:0:1 | pT2, G2, V1, R0 | severe hepatitis | 5 | 20 (*ad*) |
| Patient 10 | M, 66 | HCV | 1:1:1:1:1 | pT2, G3, V1, R0 | cirrhosis | 12 | 40 (ad) |
| Patient 11 | F, 57 | HCV | 1:0:0:1:2 | pT2, G2, V0, R0 | cirrhosis | 24 | 24 (*and*) |
| Patient 12 | M, 60 | HCV | 1:3:1:0:2 | pT2, G3, V0, R0 | cirrhosis | 84 | 84 (*and*) |
| Patient 13 | M, 74 | Alcohol | 1:0:1:0:1 | pT1, G1, V0, R0 | cirrhosis | 7 | 32 (*ad*) |
| Patient 14 | F, 70 | HCV | 1:0:0:0:1 | pT1, G1, V0, R1 | cirrhosis | 28 | 43 (*ad*) |
| Patient 15 | M, 59 | HBV | 1:0:1:0:2 | pT2, G2, V0, R0 | mild hepatitis | 6 | 6 (*ad*) |
| Patient 16 | F, 74 | *uk* | 1:1:0:1:1 | pT2, G1, V0, R1 | severe hepatitis | *uk* | *uk* |
| Patient 17 | M, 70 | *uk* | 1:1:1:0:1 | pT1, G2, V0, R0 | severe hepatitis | *uk* | *uk* |

M: male; F: female; *ad*: alive with disease; *and*: alive without disease; *dod*: died of disease; *uk*: unknown.

## Supplementary Table S2: Sequencing statistics and reads distribution

| Sample | Sequencing reads | Adapter trimmed | Length and quality filtered | rRNA reads filter out | reads in analysis | mapped reads | miRNA reads | piRNA reads | tRNA reads | Rfam annotation reads | RefGenes annotation reads | Unassigned reads |
|---|---|---|---|---|---|---|---|---|---|---|---|---|
| CIRR_1 | 39,886,295 | 39,225,918 | 2,985,609 | 135,903 | 2,849,706 | 1,548,304 | 9,300 | 3,885 | 10,468 | 14,881 | 71,196 | 1,374,192 |
| CIRR_2 | 51,489,975 | 50,828,200 | 7,464,337 | 3,990,547 | 3,473,790 | 2,426,400 | 791,165 | 103,962 | 131,203 | 118,421 | 767,014 | 474,322 |
| CIRR_3 | 40,945,296 | 40,518,388 | 5,098,872 | 2,635,893 | 2,462,979 | 1,587,651 | 151,250 | 58,317 | 224,023 | 37,950 | 239,133 | 817,488 |
| CIRR_4 | 40,000,084 | 38,882,530 | 4,084,493 | 2,151,974 | 1,932,519 | 1,141,551 | 177,915 | 50,820 | 169,140 | 55,841 | 211,311 | 432,325 |
| CIRR_6 | 40,381,965 | 39,835,305 | 4,042,964 | 718,180 | 3,324,784 | 1,359,469 | 227,150 | 73,015 | 198,498 | 37,223 | 234,925 | 505,600 |
| CIRR_7 | 34,870,179 | 34,252,889 | 10,333,629 | 3,968,752 | 6,364,877 | 4,355,480 | 1,895,221 | 128,848 | 447,510 | 127,327 | 693,909 | 960,965 |
| CIRR_9 | 46,877,444 | 45,393,999 | 17,787,722 | 8,031,220 | 9,756,502 | 7,236,109 | 1,645,205 | 521,037 | 2,780,952 | 226,012 | 553,131 | 1,373,622 |
| CIRR_11 | 18,768,870 | 18,625,875 | 1,570,572 | 1,323,383 | 247,189 | 121,170 | 16,194 | 5,507 | 11,894 | 3,444 | 32,014 | 49,147 |
| CIRR_12 | 23,341,154 | 23,021,632 | 6,984,836 | 1,977,439 | 5,007,397 | 3,791,881 | 2,041,890 | 126,754 | 159,071 | 99,826 | 655,952 | 628,648 |
| CIRR_13 | 35,756,347 | 35,209,513 | 13,312,189 | 4,972,095 | 8,340,094 | 6,231,543 | 4,015,782 | 152,989 | 422,026 | 126,145 | 579,384 | 819,491 |
| CIRR_14 | 35,090,155 | 34,731,462 | 1,698,620 | 604,940 | 1,093,680 | 724,259 | 172,671 | 23,195 | 42,680 | 37,108 | 216,255 | 218,109 |
| CIRR_15 | 28,795,563 | 28,455,139 | 11,336,225 | 1,632,476 | 9,703,749 | 8,084,226 | 5,722,948 | 411,891 | 618,618 | 130,744 | 397,735 | 677,386 |
| CIRR_16 | 27,561,943 | 27,140,271 | 7,133,108 | 2,809,612 | 4,323,496 | 3,111,859 | 1,449,725 | 141,869 | 387,411 | 84,319 | 400,786 | 596,066 |
| CIRR_17 | 27,777,226 | 27,407,597 | 8,781,463 | 3,436,690 | 5,344,773 | 3,555,663 | 1,484,299 | 98,382 | 893,586 | 69,071 | 308,650 | 652,793 |
| HCC_1 | 37,508,700 | 37,071,642 | 4,791,383 | 2,165,994 | 2,625,389 | 1,671,093 | 290,445 | 52,824 | 310,561 | 48,153 | 152,132 | 727,830 |
| HCC_1b | 41,182,406 | 40,764,018 | 3,829,002 | 1,628,965 | 2,200,037 | 1,402,791 | 213,439 | 52,540 | 135,190 | 53,692 | 330,316 | 586,035 |
| HCC_2 | 62,049,238 | 57,483,853 | 31,947,720 | 19,173,870 | 12,773,850 | 9,174,050 | 3,810,048 | 281,956 | 1,233,720 | 273,674 | 1,223,173 | 2,196,675 |
| HCC_3 | 37,411,107 | 36,040,876 | 25,088,561 | 7,899,833 | 17,188,728 | 13,214,184 | 1,605,946 | 611,649 | 9,320,261 | 203,208 | 497,596 | 872,043 |
| HCC_4 | 35,800,306 | 33,264,032 | 16,649,882 | 11,667,526 | 4,982,356 | 3,342,270 | 1,346,632 | 129,124 | 358,815 | 119,140 | 569,375 | 754,616 |
| HCC_5 | 27,923,090 | 27,442,913 | 12,377,478 | 4,946,783 | 7,430,695 | 5,486,468 | 1,139,813 | 609,825 | 2,221,161 | 249,302 | 546,374 | 675,345 |
| HCC_6 | 39,893,650 | 39,240,026 | 22,927,797 | 9,427,029 | 13,500,768 | 10,352,633 | 7,027,488 | 328,292 | 807,492 | 191,920 | 685,457 | 1,160,575 |
| HCC_6b | 41,600,752 | 38,263,542 | 22,461,249 | 12,249,960 | 10,211,289 | 7,158,257 | 3,889,616 | 247,372 | 701,810 | 166,265 | 764,229 | 1,269,057 |
| HCC_7 | 36,806,364 | 35,164,151 | 15,880,386 | 6,537,212 | 9,343,174 | 6,960,685 | 3,197,051 | 181,627 | 1,018,411 | 153,867 | 574,144 | 1,726,659 |
| HCC_8 | 31,162,799 | 30,117,190 | 15,096,303 | 8,188,194 | 6,908,109 | 4,551,260 | 2,581,396 | 159,097 | 338,858 | 100,791 | 394,978 | 888,424 |
| HCC_9 | 41,948,446 | 41,378,741 | 11,872,833 | 4,570,161 | 7,302,672 | 5,186,427 | 1,283,320 | 408,912 | 1,954,216 | 191,487 | 343,825 | 922,378 |
| HCC_10 | 34,195,204 | 33,596,761 | 11,180,810 | 5,035,308 | 6,145,502 | 4,532,665 | 3,044,405 | 157,170 | 220,546 | 92,330 | 326,360 | 630,499 |
| HCC_11 | 23,512,360 | 23,216,346 | 11,649,127 | 4,133,579 | 7,515,548 | 5,249,527 | 1,590,020 | 309,444 | 880,076 | 203,856 | 694,686 | 1,485,000 |
| HCC_11b | 30,229,052 | 29,664,930 | 17,152,692 | 7,014,189 | 10,138,503 | 7,869,158 | 4,261,155 | 239,803 | 725,587 | 224,131 | 721,905 | 1,591,576 |
| HCC_12 | 26,023,524 | 25,553,155 | 12,562,895 | 4,422,712 | 8,140,183 | 5,901,802 | 744,926 | 639,929 | 3,334,135 | 358,117 | 337,610 | 455,880 |
| HCC_12b | 30,664,580 | 30,019,781 | 15,570,970 | 5,418,943 | 10,152,027 | 7,491,423 | 1,057,392 | 820,608 | 4,100,452 | 360,185 | 444,009 | 675,238 |
| HCC_13 | 34,189,349 | 32,698,076 | 23,348,482 | 3,851,814 | 19,496,668 | 15,380,555 | 3,625,228 | 572,002 | 9,354,847 | 124,849 | 720,756 | 891,802 |
| HCC_14 | 51,175,779 | 50,424,281 | 22,156,136 | 7,576,892 | 14,579,244 | 11,177,124 | 3,826,041 | 481,468 | 4,623,063 | 235,730 | 941,759 | 984,915 |
| HCC_15 | 30,227,699 | 29,694,810 | 18,313,170 | 1,426,157 | 16,887,013 | 13,355,602 | 3,673,484 | 415,710 | 5,390,310 | 137,609 | 484,294 | 3,117,887 |
| HCC_15b | 30,375,300 | 29,911,820 | 18,565,205 | 861,261 | 17,703,944 | 14,325,368 | 3,535,989 | 373,143 | 7,852,606 | 103,463 | 321,983 | 2,055,014 |

Small RNA sequencing statistics and distribution of reads mapped in different RNA annotation.

**Supplementary Table S3A: piRNA expression in cirrhosis and HCC tissues.** See Supplementary_Table_S3A

**Supplementary Table S3B: Annotation of liver piRNA.** See Supplementary_Table_S3B

**Supplementary Table S4: piRNAs differential expressed between cirrhosis and HCC tissues.** See Supplementary_Table_S4

## Supplementary Table S5: piRNAs differentially expressed in different cancer tissues

| piRNA ID | Breast cancer *[1–S]* | Endometrial cancer *[2–S]* | Gastric cancer *[3–S]* | Gastric cancer *[4–S]* | Myeloma *[5–S]* | Renal carcinoma *[6–S]* | Multiple cancer types *[7–S]* |
|---|---|---|---|---|---|---|---|
| has_piR_000823 | | | X | | X | | |
| hsa_piR_001042 | | | | X | | | X |
| hsa_piR_001152 | | X | | X | | | X |
| has_piR_001170 | | X | | | | X | |
| hsa_piR_001205 | | | | X | | | X |
| has_piR_003728 | | | | | | | X |
| hsa_piR_007336 | | | | X | | | X |
| has_piR_009051 | | | | | | | X |
| has_piR_010894 | | | | X | | X | |
| has_piR_011901 | | | | | | | X |
| has_piR_012681 | | | | | | | X |
| hsa_piR_013306 | | | | X | | | X |
| has_piR_016664 | | | | | | | X |
| hsa_piR_016975 | | | | | | | X |
| has_piR_017033 | | | | | | | X |
| hsa_piR_017061 | | | | X | | | X |
| hsa_piR_017791 | | | | X | | | X |
| hsa_piR_011901 | | | | X | | | |
| hsa_piR_012681 | | | | X | | | |
| hsa_piR_014620 | | | | X | | | |
| hsa_piR_018165 | | | | | | | X |
| hsa_piR_019102 | | | | X | | | X |
| has_piR_019168 | X | X | | | | | |
| has_piR_019368 | | | | | | | X |
| has_piR_019420 | X | | | X | | | X |
| has_piR_020009 | | X | | | | X | X |
| has_piR_020305 | | | | | | | X |
| has_piR_020499 | X | | | | | | |
| hsa_piR_020450 | | | | X | | | |
| hsa_piR_020657 | | | | X | | | X |
| hsa_piR_020814 | | | | X | | | X |
| has_piR_020829 | X | | | | | | |
| has_piR_023057 | | | | X | | | X |

X: piRNA differentially expressed in cancer tissues.


*1–S.* Hashim A, Rizzo F, Marchese G, Ravo M, Tarallo R, Nassa G, Giurato G, Santamaria G, Cordella A, Cantarella C, Weisz A. RNA sequencing identifies specific PIWI–interacting small non–coding RNA expression patterns in breast cancer. Oncotarget. 2014; 5:9901–9910.

*2–S.* Ravo M, Cordella A, Rinaldi A, Bruno G, Alexandrova E, Saggese P, Nassa G, Giurato G, Tarallo R, Marchese G, Rizzo F, Stellato C, Biancardi R, Troisi J, Di Spiezio Sardo A, Zullo F, et al. Small non–coding RNA deregulation in endometrial carcinogenesis. Oncotarget. 2015; 6:4677–4691.

*3–S.* Cheng J, Deng H, Xiao B, Zhou H, Zhou F, Shen Z, Guo J. piR–823, a novel non–coding small RNA, demonstrates in vitro and in vivo tumor suppressive activity in human gastric cancer cells. Cancer Lett. 2012; 315:12–17.

*4–S.* Martinez VD, Enfield KS, Rowbotham DA, Lam WL. An atlas of gastric PIWI–interacting RNA transcriptomes and their utility for identifying signatures of gastric cancer recurrence. Gastric Cancer. 2015; 19:660–665.

*5–S.* Yan H, Wu QL, Sun CY, Ai LS, Deng J, Zhang L, Chen L, Chu ZB, Tang B, Wang K, Wu XF, Xu J, Hu Y. piRNA–823 contributes to tumorigenesis by regulating de novo DNA methylation and angiogenesis in multiple myeloma. Leukemia. 2015; 29:196–206.

*6–S.* Li Y, Wu X, Gao H, Jin JM, Li AX, Kim YS, Pal SK, Nelson RA, Lau CM, Guo C, Mu B, Wang J, Wang F, Zhao Y, Chen W, Rossi JJ, et al. PIWI–interacting RNAs are dysregulated in renal cell carcinoma and associated with tumor metastasis and cancer specific survival. Mol Med. 2015; 21:381–388

*7–S.* Martinez VD, Vucic EA, Thu KL, Hubaux R, Enfield KS, Pikor LA, Becker–Santos DD, Brown CJ, Lam S, Lam WL. Unique somatic and malignant expression patterns implicate PIWI–interacting RNAs in cancer–type specific biology. Sci Rep. 2015; 5:10423.

**Supplementary Table S6A: piRNA–like expression in cirrhosis and HCC tissues.** See Supplementary_Table_S6A

**Supplementary Table S6B: Annotation of liver piRNA–Like.** See Supplementary_Table_S6B

**Supplementary Table S7: piRNA–like differential expression analysis between cirrhosis and HCC tissues.** See Supplementary_Table_S7

**Supplementary Table S8: Top canonical pathways predicted to be target of piRNAs deregulated in HCC.** See Supplementary_Table_S8
