# Supplementary material for: Specific patterns of PIWI-interacting small noncoding RNA expression in dysplastic liver nodules and hepatocellular carcinoma
Source: Oncotarget. 2016 Jul 13;7(34):54650–61. doi: 10.18632/oncotarget.10567 (PMC5342370; doi:10.18632/oncotarget.10567)
Supplement: Supplementary file 2 [file oncotarget-07-54650-s002.docx]

| Supplementary Table S3A: piRNA expression in cirrhosis and HCC tissues | | |
| --- | --- | --- |
| **piRNA ID** | **Median CIRR** | **Median HCC** |
| hsa_piR_020326 | 162361 | 200512 |
| hsa_piR_000765 | 162361 | 200510 |
| hsa_piR_004153 | 69509 | 61133 |
| hsa_piR_017716 | 69474 | 61105 |
| hsa_piR_001042 | 66498 | 6277 |
| hsa_piR_018570 | 59375 | 85702 |
| hsa_piR_012681 | 58367 | 14032 |
| hsa_piR_005019 | 55571 | 12587 |
| hsa_piR_001312 | 47992 | 65640 |
| hsa_piR_018569 | 41016 | 56577 |
| hsa_piR_017033 | 37448 | 17752 |
| hsa_piR_015026 | 32395 | 45370 |
| hsa_piR_001152 | 31752 | 4895 |
| hsa_piR_020829 | 24229 | 55613 |
| hsa_piR_019912 | 15899 | 10175 |
| hsa_piR_001311 | 14256 | 20413 |
| hsa_piR_020365 | 12497 | 31466 |
| hsa_piR_020450 | 10115 | 4091 |
| hsa_piR_020813 | 10093 | 6987 |
| hsa_piR_018780 | 9888 | 9503 |
| hsa_piR_020485 | 9612 | 16272 |
| hsa_piR_000552 | 9458 | 5625 |
| hsa_piR_019752 | 8478 | 2005 |
| hsa_piR_015249 | 8035 | 8914 |
| hsa_piR_004987 | 7265 | 22243 |
| hsa_piR_016735 | 7111 | 16499 |
| hsa_piR_009295 | 7023 | 6498 |
| hsa_piR_019825 | 6350 | 16224 |
| hsa_piR_017724 | 6209 | 19631 |
| hsa_piR_001346 | 6102 | 3115 |
| hsa_piR_020809 | 5142 | 402 |
| hsa_piR_016240 | 5050 | 407 |
| hsa_piR_017458 | 4949 | 3653 |
| hsa_piR_004308 | 4872 | 15253 |
| hsa_piR_004152 | 4815 | 4728 |
| hsa_piR_004307 | 4748 | 13771 |
| hsa_piR_017061 | 4724 | 1866 |
| hsa_piR_016658 | 4665 | 8761 |
| hsa_piR_019050 | 4591 | 2371 |
| hsa_piR_009051 | 3637 | 25617 |
| hsa_piR_016946 | 3597 | 203 |
| hsa_piR_001101 | 3080 | 2068 |
| hsa_piR_000441 | 2724 | 5296 |
| hsa_piR_019914 | 2712 | 9055 |
| hsa_piR_004309 | 2702 | 9673 |
| hsa_piR_019676 | 2660 | 3091 |
| hsa_piR_000586 | 2575 | 2028 |
| hsa_piR_004801 | 2531 | 716 |
| hsa_piR_020009 | 2458 | 24525 |
| hsa_piR_001159 | 2370 | 2429 |
| hsa_piR_017723 | 2312 | 5365 |
| hsa_piR_016677 | 2193 | 1823 |
| hsa_piR_019420 | 1941 | 568 |
| hsa_piR_016945 | 1925 | 3813 |
| hsa_piR_020814 | 1722 | 949 |
| hsa_piR_019951 | 1688 | 3927 |
| hsa_piR_017295 | 1585 | 244 |
| hsa_piR_017791 | 1510 | 573 |
| hsa_piR_000045 | 1472 | 2504 |
| hsa_piR_020496 | 1460 | 2806 |
| hsa_piR_020500 | 1443 | 3664 |
| hsa_piR_020497 | 1427 | 2245 |
| hsa_piR_021190 | 1403 | 87 |
| hsa_piR_016659 | 1345 | 3472 |
| hsa_piR_004993 | 1294 | 1059 |
| hsa_piR_014620 | 1173 | 600 |
| hsa_piR_020541 | 1133 | 524 |
| hsa_piR_016745 | 1062 | 3392 |
| hsa_piR_020582 | 1060 | 3341 |
| hsa_piR_000805 | 991 | 4638 |
| hsa_piR_002158 | 796 | 2315 |
| hsa_piR_000823 | 745 | 2143 |
| hsa_piR_005018 | 662 | 94 |
| hsa_piR_020388 | 600 | 723 |
| hsa_piR_018292 | 542 | 1193 |
| hsa_piR_008033 | 496 | 30 |
| hsa_piR_019168 | 488 | 124 |
| hsa_piR_010894 | 456 | 2379 |
| hsa_piR_019102 | 445 | 75 |
| hsa_piR_001168 | 418 | 376 |
| hsa_piR_004800 | 417 | 138 |
| hsa_piR_017184 | 361 | 1259 |
| hsa_piR_001170 | 351 | 4166 |
| hsa_piR_016970 | 327 | 1640 |
| hsa_piR_008488 | 308 | 290 |
| hsa_piR_016926 | 295 | 326 |
| hsa_piR_012753 | 273 | 347 |
| hsa_piR_016742 | 271 | 642 |
| hsa_piR_017178 | 270 | 631 |
| hsa_piR_002468 | 252 | 101 |
| hsa_piR_020619 | 247 | 314 |
| hsa_piR_019224 | 242 | 56 |
| hsa_piR_001207 | 235 | 169 |
| hsa_piR_015254 | 219 | 115 |
| hsa_piR_020815 | 216 | 536 |
| hsa_piR_017104 | 214 | 243 |
| hsa_piR_022628 | 214 | 156 |
| hsa_piR_021214 | 202 | 47 |
| hsa_piR_020499 | 180 | 359 |
| hsa_piR_001169 | 162 | 408 |
| hsa_piR_017194 | 154 | 345 |
| hsa_piR_000291 | 145 | 1128 |
| hsa_piR_016980 | 144 | 210 |
| hsa_piR_000560 | 140 | 254 |
| hsa_piR_020439 | 137 | 84 |
| hsa_piR_020828 | 129 | 943 |
| hsa_piR_001205 | 127 | 359 |
| hsa_piR_019521 | 118 | 237 |
| hsa_piR_020466 | 118 | 221 |
| hsa_piR_016984 | 115 | 197 |
| hsa_piR_019201 | 113 | 28 |
| hsa_piR_001078 | 111 | 37 |
| piR–Hep1 | 99 | 238 |
| hsa_piR_018165 | 92 | 171 |
| hsa_piR_019354 | 91 | 194 |
| hsa_piR_005271 | 88 | 104 |
| hsa_piR_016963 | 85 | 16 |
| hsa_piR_000796 | 70 | 56 |
| hsa_piR_004506 | 55 | 70 |
| hsa_piR_019949 | 53 | 138 |
| hsa_piR_020008 | 48 | 130 |
| hsa_piR_019167 | 47 | 79 |
| hsa_piR_005660 | 44 | 38 |
| hsa_piR_012925 | 29 | 43 |
| hsa_piR_016239 | 26 | 2 |
| hsa_piR_011901 | 26 | 2 |
| hsa_piR_020364 | 24 | 153 |
| hsa_piR_016975 | 24 | 148 |
| hsa_piR_020362 | 24 | 129 |
| hsa_piR_020363 | 24 | 128 |
| hsa_piR_004150 | 24 | 31 |
| hsa_piR_007635 | 24 | 18 |
| hsa_piR_019911 | 23 | 26 |
| hsa_piR_013306 | 22 | 108 |
| hsa_piR_010541 | 21 | 38 |
| hsa_piR_020657 | 18 | 107 |
| hsa_piR_022794 | 17 | 8 |
| hsa_piR_020391 | 15 | 29 |
| hsa_piR_007336 | 15 | 0 |
| hsa_piR_003728 | 12 | 44 |
| hsa_piR_020793 | 11 | 16 |
| hsa_piR_003239 | 10 | 3 |
| hsa_piR_019824 | 9 | 27 |
| hsa_piR_002732 | 8 | 2 |
| hsa_piR_020305 | 7 | 20 |
| hsa_piR_007232 | 5 | 2 |
| hsa_piR_019368 | 4 | 70 |
| hsa_piR_019823 | 4 | 16 |
| hsa_piR_019628 | 4 | 13 |
| hsa_piR_016664 | 2 | 29 |
| hsa_piR_000801 | 2 | 12 |
| hsa_piR_000794 | 2 | 0 |
| hsa_piR_010155 | 0 | 25 |
| hsa_piR_019822 | 0 | 20 |
| hsa_piR_005076 | 0 | 11 |
| hsa_piR_013350 | 0 | 8 |
| hsa_piR_019574 | 0 | 3 |
| hsa_piR_020498 | 0 | 3 |
| hsa_piR_011537 | 0 | 2 |
| hsa_piR_011968 | 0 | 2 |
| hsa_piR_023057 | 0 | 2 |
| hsa_piR_007150 | 0 | 1 |
| hsa_piR_019169 | 0 | 1 |
| hsa_piR_006613 | 0 | 1 |
| hsa_piR_020575 | 0 | 0 |
| hsa_piR_011209 | 0 | 0 |
| hsa_piR_017809 | 0 | 0 |
| hsa_piR_018811 | 0 | 0 |
| hsa_piR_002973 | 0 | 0 |
| hsa_piR_012734 | 0 | 0 |
| hsa_piR_009894 | 0 | 0 |
| hsa_piR_003785 | 0 | 0 |
| hsa_piR_020345 | 0 | 0 |
| hsa_piR_009502 | 0 | 0 |
| hsa_piR_001421 | 0 | 0 |
| hsa_piR_000330 | 0 | 0 |
| hsa_piR_019166 | 0 | 0 |
| hsa_piR_011181 | 0 | 0 |
| hsa_piR_004216 | 0 | 0 |
| hsa_piR_001179 | 0 | 0 |
| hsa_piR_000925 | 0 | 0 |
| hsa_piR_011398 | 0 | 0 |
| hsa_piR_001925 | 0 | 0 |
| hsa_piR_018380 | 0 | 0 |
| hsa_piR_009237 | 0 | 0 |
| hsa_piR_020492 | 0 | 0 |
| hsa_piR_022236 | 0 | 0 |
| hsa_piR_008683 | 0 | 0 |
| hsa_piR_009016 | 0 | 0 |
| hsa_piR_022114 | 0 | 0 |
| hsa_piR_012558 | 0 | 0 |
| hsa_piR_005336 | 0 | 0 |
| hsa_piR_022113 | 0 | 0 |
| hsa_piR_015476 | 0 | 0 |
| hsa_piR_021764 | 0 | 0 |
| hsa_piR_011300 | 0 | 0 |
| hsa_piR_018596 | 0 | 0 |
| Expression value (read per million) of 197 piRNAs detected in cirrhosis and HCC tissues. | | |
