# Supplementary material for: Specific patterns of PIWI-interacting small noncoding RNA expression in dysplastic liver nodules and hepatocellular carcinoma
Source: Oncotarget. 2016 Jul 13;7(34):54650–61. doi: 10.18632/oncotarget.10567 (PMC5342370; doi:10.18632/oncotarget.10567)
Supplement: Supplementary file 3 [file oncotarget-07-54650-s003.docx]

| Supplementary Table S3B: Annotation of liver piRNA | | | | | | | | | | |
| --- | --- | --- | --- | --- | --- | --- | --- | --- | --- | --- |
| **piRNA_ID** | **piRNA_chr** | **piRNA_start** | **piRNA_end** | **piRNA_strand** | **Gene_Region** | **Gene_name** | **Gene_chr** | **Gene_start** | **Gene_end** | **Gene_strand** |
| hsa_piR_003785 | chr10 | 82032585 | 82032614 | – | 3_UTR | MAT1A | chr10 | 82031575 | 82033536 | – |
| hsa_piR_020814 | chr22 | 39709882 | 39709913 | – | INTRON | RPL3 | chr22 | 39709734 | 39710111 | – |
| hsa_piR_002732 | chr22 | 39709884 | 39709911 | – | INTRON | RPL3 | chr22 | 39709734 | 39710111 | – |
| hsa_piR_000552 | chr22 | 39715057 | 39715084 | – | INTRON | RPL3 | chr22 | 39714597 | 39715599 | – |
| hsa_piR_019050 | chr15 | 66795150 | 66795177 | – | INTRON | RPL4 | chr15 | 66795088 | 66795395 | – |
| hsa_piR_010541 | chr9 | 136217702 | 136217733 | + | INTRON | RPL7A | chr9 | 136217582 | 136217856 | + |
| hsa_piR_001207 | chr16 | 89627859 | 89627884 | + | INTRON | RPL13 | chr16 | 89627776 | 89627985 | + |
| hsa_piR_016664 | chr17 | 37009222 | 37009248 | – | INTRON | RPL23 | chr17 | 37008985 | 37009274 | – |
| hsa_piR_000925 | chr19 | 49121048 | 49121076 | – | CDS | RPL18 | chr19 | 49121047 | 49121134 | – |
| hsa_piR_000560 | chr17 | 27047601 | 27047631 | + | INTRON | RPL23A | chr17 | 27047048 | 27047724 | + |
| hsa_piR_001346 | chr17 | 27049639 | 27049667 | + | INTRON | RPL23A | chr17 | 27047908 | 27049740 | + |
| hsa_piR_018292 | chr18 | 47015648 | 47015678 | – | INTRON | RPL17 | chr18 | 47014936 | 47015728 | – |
| hsa_piR_017178 | chr18 | 47017687 | 47017717 | – | INTRON | RPL17 | chr18 | 47017297 | 47017774 | – |
| hsa_piR_017194 | chr18 | 47018068 | 47018099 | – | 5_UTR | RPL17 | chr18 | 47017941 | 47018203 | – |
| hsa_piR_001205 | chr11 | 8705774 | 8705805 | + | INTRON | RPL27A | chr11 | 8705628 | 8706264 | + |
| hsa_piR_009894 | chr19 | 55897739 | 55897764 | + | CDS | RPL28 | chr19 | 55897725 | 55897806 | + |
| hsa_piR_017791 | chr11 | 75111436 | 75111465 | + | INTRON | RPS3 | chr11 | 75110621 | 75111737 | + |
| hsa_piR_004506 | chrY | 25342907 | 25342934 | – | INTRON | DAZ4 | chrY | 25338345 | 25344944 | – |
| hsa_piR_004506 | chrY | 26982287 | 26982314 | + | INTRON | DAZ4 | chrY | 26980276 | 26986877 | + |
| hsa_piR_004506 | chrY | 25367901 | 25367928 | + | INTRON | DAZ2 | chrY | 25365890 | 25372490 | + |
| hsa_piR_004506 | chrY | 26982287 | 26982314 | + | INTRON | DAZ2 | chrY | 26980276 | 26997725 | + |
| hsa_piR_004506 | chrY | 25367901 | 25367928 | + | INTRON | DAZ2 | chrY | 25365890 | 25372490 | + |
| hsa_piR_004506 | chrY | 26982287 | 26982314 | + | INTRON | DAZ2 | chrY | 26980276 | 26997725 | + |
| hsa_piR_007635 | chr11 | 46784021 | 46784047 | – | INTRON | CKAP5 | chr11 | 46783743 | 46784176 | – |
| hsa_piR_001101 | chr1 | 45244100 | 45244127 | + | INTRON | RPS8 | chr1 | 45243801 | 45244247 | + |
| hsa_piR_008683 | chr6 | 34392969 | 34392994 | – | CDS | RPS10 | chr6 | 34392848 | 34392998 | – |
| hsa_piR_017104 | chr6 | 133137966 | 133137994 | + | INTRON | RPS12 | chr6 | 133137702 | 133138098 | + |
| hsa_piR_005271 | chr6 | 133137967 | 133137992 | + | INTRON | RPS12 | chr6 | 133137702 | 133138098 | + |
| hsa_piR_012925 | chr1 | 31408589 | 31408618 | – | INTRON | PUM1 | chr1 | 31406189 | 31409489 | – |
| hsa_piR_012925 | chr1 | 31422018 | 31422047 | – | INTRON | PUM1 | chr1 | 31418330 | 31422973 | – |
| hsa_piR_020813 | chr1 | 31441013 | 31441042 | – | INTRON | PUM1 | chr1 | 31440157 | 31441200 | – |
| hsa_piR_017458 | chr1 | 31441015 | 31441041 | – | INTRON | PUM1 | chr1 | 31440157 | 31441200 | – |
| hsa_piR_005336 | chr8 | 56986264 | 56986290 | – | CDS | RPS20 | chr8 | 56986253 | 56986327 | – |
| hsa_piR_013306 | chr8 | 56986429 | 56986460 | – | INTRON | RPS20 | chr8 | 56986327 | 56986618 | – |
| hsa_piR_002158 | chr16 | 71792312 | 71792340 | – | INTRON | AP1G1 | chr16 | 71790062 | 71792710 | – |
| hsa_piR_020814 | chr22 | 39709882 | 39709913 | – | INTRON | RPL3 | chr22 | 39709734 | 39710111 | – |
| hsa_piR_002732 | chr22 | 39709884 | 39709911 | – | INTRON | RPL3 | chr22 | 39709734 | 39710111 | – |
| hsa_piR_000552 | chr22 | 39715057 | 39715084 | – | INTRON | RPL3 | chr22 | 39714597 | 39715599 | – |
| hsa_piR_018292 | chr18 | 47015648 | 47015678 | – | INTRON | RPL17 | chr18 | 47014936 | 47015728 | – |
| hsa_piR_017178 | chr18 | 47017687 | 47017717 | – | INTRON | RPL17 | chr18 | 47017297 | 47017774 | – |
| hsa_piR_017194 | chr18 | 47018068 | 47018099 | – | INTRON | RPL17 | chr18 | 47017954 | 47018827 | – |
| hsa_piR_004987 | chr1 | 147774888 | 147774918 | – | INTRON | NBPF8 | chr1 | 147576928 | 148006225 | – |
| hsa_piR_001152 | chr2 | 207026645 | 207026674 | + | INTRON | EEF1B2 | chr2 | 207026196 | 207026761 | + |
| hsa_piR_022794 | chr14 | 70235682 | 70235710 | + | INTRON | SRSF5 | chr14 | 70235613 | 70235898 | + |
| hsa_piR_017809 | chr1 | 44436748 | 44436774 | + | CDS | DPH2 | chr1 | 44436637 | 44436861 | + |
| hsa_piR_002973 | chr1 | 145438509 | 145438537 | + | INTRON | NBPF10 | chr1 | 145365459 | 146420910 | + |
| hsa_piR_011181 | chr7 | 144345474 | 144345500 | – | INTRON | TPK1 | chr7 | 144320354 | 144345899 | – |
| hsa_piR_015476 | chr16 | 29819002 | 29819031 | + | CDS | MAZ | chr16 | 29818298 | 29819149 | + |
| hsa_piR_001421 | chr17 | 56082521 | 56082549 | – | 3_UTR | SRSF1 | chr17 | 56078279 | 56083107 | – |
| hsa_piR_012558 | chr10 | 81272792 | 81272821 | + | CDS | EIF5AL1 | chr10 | 81272405 | 81272870 | + |
| hsa_piR_022113 | chr7 | 5568123 | 5568152 | – | CDS | ACTB | chr7 | 5567911 | 5568350 | – |
| hsa_piR_019822 | chr12 | 50610770 | 50610797 | – | INTRON | LIMA1 | chr12 | 50599851 | 50615803 | – |
| hsa_piR_019822 | chr12 | 50610770 | 50610797 | – | INTRON | LIMA1 | chr12 | 50599851 | 50615803 | – |
| hsa_piR_002158 | chr16 | 71792312 | 71792340 | – | INTRON | AP1G1 | chr16 | 71790062 | 71792710 | – |
| hsa_piR_020575 | chr13 | 99639319 | 99639345 | – | INTRON | DOCK9 | chr13 | 99607805 | 99738476 | – |
| hsa_piR_020582 | chr11 | 35001077 | 35001105 | + | INTRON | PDHX | chr11 | 34999729 | 35006116 | + |
| hsa_piR_020364 | chr2 | 51163342 | 51163367 | – | INTRON | NRXN1 | chr2 | 51153093 | 51253508 | – |
| hsa_piR_009894 | chr19 | 55897739 | 55897764 | + | CDS | RPL28 | chr19 | 55897725 | 55897806 | + |
| hsa_piR_009894 | chr19 | 55897739 | 55897764 | + | CDS | RPL28 | chr19 | 55897725 | 55897806 | + |
| hsa_piR_009894 | chr19 | 55897739 | 55897764 | + | CDS | RPL28 | chr19 | 55897725 | 55897806 | + |
| hsa_piR_009894 | chr19 | 55897739 | 55897764 | + | CDS | RPL28 | chr19 | 55897725 | 55897806 | + |
| hsa_piR_020439 | chr19 | 12817303 | 12817331 | – | INTRON | TNPO2 | chr19 | 12817181 | 12817383 | – |
| hsa_piR_020439 | chr19 | 12817303 | 12817331 | – | INTRON | TNPO2 | chr19 | 12817181 | 12817383 | – |
| hsa_piR_014620 | chr5 | 93905174 | 93905200 | – | INTRON | KIAA0825 | chr5 | 93872832 | 93918137 | – |
| hsa_piR_005336 | chr8 | 56986264 | 56986290 | – | CDS | RPS20 | chr8 | 56986253 | 56986327 | – |
| hsa_piR_013306 | chr8 | 56986429 | 56986460 | – | INTRON | RPS20 | chr8 | 56986327 | 56986618 | – |
| hsa_piR_004216 | chr15 | 82764337 | 82764367 | – | INTRON | GOLGA6L10 | chr15 | 82635575 | 82933938 | – |
| hsa_piR_020582 | chr11 | 35001077 | 35001105 | + | INTRON | PDHX | chr11 | 34969153 | 35006116 | + |
| hsa_piR_009016 | chr12 | 123776709 | 123776739 | – | 3_UTR | SBNO1 | chr12 | 123773655 | 123780454 | – |
| hsa_piR_019911 | chr14 | 21860382 | 21860413 | – | INTRON | CHD8 | chr14 | 21860105 | 21860665 | – |
| hsa_piR_003728 | chr14 | 21865530 | 21865560 | – | INTRON | CHD8 | chr14 | 21864051 | 21865981 | – |
| hsa_piR_013350 | chr2 | 234197555 | 234197584 | + | INTRON | ATG16L1 | chr2 | 234191399 | 234198499 | + |
| hsa_piR_013350 | chr2 | 234197555 | 234197584 | + | INTRON | ATG16L1 | chr2 | 234191399 | 234198499 | + |
| hsa_piR_001168 | chr7 | 142375404 | 142375431 | + | 3_UTR | MTRNR2L6 | chr7 | 142375137 | 142375525 | + |
| hsa_piR_020008 | chr11 | 10530444 | 10530474 | – | 5_UTR | MTRNR2L8 | chr11 | 10529773 | 10530723 | – |
| hsa_piR_018292 | chr18 | 47015648 | 47015678 | – | INTRON | RPL17 | chr18 | 47014936 | 47015728 | – |
| hsa_piR_017178 | chr18 | 47017687 | 47017717 | – | INTRON | RPL17 | chr18 | 47017297 | 47017774 | – |
| hsa_piR_017194 | chr18 | 47018068 | 47018099 | – | INTRON | RPL17 | chr18 | 47017954 | 47018105 | – |
| hsa_piR_018292 | chr18 | 47015648 | 47015678 | – | INTRON | RPL17 | chr18 | 47014936 | 47015728 | – |
| hsa_piR_017178 | chr18 | 47017687 | 47017717 | – | INTRON | RPL17 | chr18 | 47017297 | 47017774 | – |
| hsa_piR_017194 | chr18 | 47018068 | 47018099 | – | INTRON | RPL17 | chr18 | 47017954 | 47018627 | – |
| hsa_piR_018292 | chr18 | 47015648 | 47015678 | – | INTRON | RPL17 | chr18 | 47014936 | 47015728 | – |
| hsa_piR_017178 | chr18 | 47017687 | 47017717 | – | INTRON | RPL17 | chr18 | 47017297 | 47017774 | – |
| hsa_piR_017194 | chr18 | 47018068 | 47018099 | – | INTRON | RPL17 | chr18 | 47017954 | 47018105 | – |
| hsa_piR_018292 | chr18 | 47015648 | 47015678 | – | INTRON | RPL17 | chr18 | 47014936 | 47015728 | – |
| hsa_piR_017178 | chr18 | 47017687 | 47017717 | – | INTRON | RPL17 | chr18 | 47017297 | 47017774 | – |
| hsa_piR_017194 | chr18 | 47018068 | 47018099 | – | INTRON | RPL17 | chr18 | 47017954 | 47018105 | – |
| hsa_piR_018292 | chr18 | 47015648 | 47015678 | – | INTRON | RPL17 | chr18 | 47014936 | 47015728 | – |
| hsa_piR_017178 | chr18 | 47017687 | 47017717 | – | INTRON | RPL17 | chr18 | 47017297 | 47017774 | – |
| hsa_piR_017194 | chr18 | 47018068 | 47018099 | – | INTRON | RPL17 | chr18 | 47017954 | 47018644 | – |
| hsa_piR_018292 | chr18 | 47015648 | 47015678 | – | INTRON | RPL17 | chr18 | 47014936 | 47015728 | – |
| hsa_piR_017178 | chr18 | 47017687 | 47017717 | – | INTRON | RPL17 | chr18 | 47017297 | 47017901 | – |
| hsa_piR_017194 | chr18 | 47018068 | 47018099 | – | INTRON | RPL17 | chr18 | 47017954 | 47018827 | – |
| hsa_piR_018292 | chr18 | 47015648 | 47015678 | – | INTRON | RPL17–C18orf32 | chr18 | 47010141 | 47015728 | – |
| hsa_piR_017178 | chr18 | 47017687 | 47017717 | – | INTRON | RPL17–C18orf32 | chr18 | 47017297 | 47017774 | – |
| hsa_piR_018292 | chr18 | 47015648 | 47015678 | – | INTRON | RPL17–C18orf32 | chr18 | 47010141 | 47015728 | – |
| hsa_piR_017178 | chr18 | 47017687 | 47017717 | – | INTRON | RPL17–C18orf32 | chr18 | 47017297 | 47017901 | – |
| hsa_piR_017194 | chr18 | 47018068 | 47018099 | – | INTRON | RPL17–C18orf32 | chr18 | 47017954 | 47018827 | – |
| hsa_piR_022114 | chr17 | 79478425 | 79478454 | – | CDS | ACTG1 | chr17 | 79478213 | 79478652 | – |
| hsa_piR_021764 | chr1 | 45980634 | 45980661 | – | CDS | PRDX1 | chr1 | 45980544 | 45980667 | – |
| hsa_piR_008683 | chr6 | 34392969 | 34392994 | – | CDS | RPS10–NUDT3 | chr6 | 34392848 | 34392998 | – |
| hsa_piR_008683 | chr6 | 34392969 | 34392994 | – | CDS | RPS10 | chr6 | 34392848 | 34392998 | – |
| hsa_piR_008683 | chr6 | 34392969 | 34392994 | – | CDS | RPS10 | chr6 | 34392848 | 34392998 | – |
| hsa_piR_011398 | chr17 | 7479915 | 7479943 | + | CDS | EIF4A1 | chr17 | 7479841 | 7480010 | + |
| hsa_piR_022236 | chr6 | 29910611 | 29910640 | + | CDS | HLA–A | chr6 | 29910533 | 29910803 | + |
| hsa_piR_022236 | chr6 | 31239539 | 31239568 | – | CDS | HLA–C | chr6 | 31239375 | 31239645 | – |
| hsa_piR_001207 | chr16 | 89627859 | 89627884 | + | INTRON | RPL13 | chr16 | 89627737 | 89628003 | + |
| hsa_piR_001207 | chr16 | 89627859 | 89627884 | + | INTRON | RPL13 | chr16 | 89627776 | 89627985 | + |
| hsa_piR_019628 | chr17 | 18157227 | 18157253 | – | INTRON | FLII | chr17 | 18157033 | 18157392 | – |
| hsa_piR_019628 | chr17 | 18157227 | 18157253 | – | INTRON | FLII | chr17 | 18157033 | 18157392 | – |
| hsa_piR_017791 | chr11 | 75111436 | 75111465 | + | INTRON | RPS3 | chr11 | 75110621 | 75111737 | + |
| hsa_piR_017791 | chr11 | 75111436 | 75111465 | + | INTRON | RPS3 | chr11 | 75110621 | 75111737 | + |
| hsa_piR_017791 | chr11 | 75111436 | 75111465 | + | INTRON | RPS3 | chr11 | 75110621 | 75112683 | + |
| hsa_piR_010155 | chr7 | 45144052 | 45144081 | – | INTRON | TBRG4 | chr7 | 45143855 | 45144136 | – |
| hsa_piR_008683 | chr11 | 46450671 | 46450696 | – | INTRON | AMBRA1 | chr11 | 46439602 | 46455023 | – |
| hsa_piR_008683 | chr11 | 46450671 | 46450696 | – | INTRON | AMBRA1 | chr11 | 46439602 | 46455023 | – |
| hsa_piR_000925 | chr19 | 49121048 | 49121076 | – | INTRON | RPL18 | chr19 | 49120680 | 49122397 | – |
| hsa_piR_016975 | chr19 | 49994164 | 49994191 | + | INTRON | RPL13A | chr19 | 49994121 | 49994296 | + |
| hsa_piR_020305 | chr19 | 49994432 | 49994457 | + | INTRON | RPL13A | chr19 | 49994356 | 49994681 | + |
| hsa_piR_015476 | chr16 | 29819002 | 29819031 | + | CDS | MAZ | chr16 | 29818298 | 29819149 | + |
| hsa_piR_015476 | chr16 | 29819002 | 29819031 | + | INTRON | MAZ | chr16 | 29818214 | 29819890 | + |
| hsa_piR_002973 | chr1 | 145438509 | 145438537 | + | INTRON | NBPF20 | chr1 | 145364674 | 146420018 | + |
| hsa_piR_017033 | chr20 | 17943355 | 17943384 | – | INTRON | SNX5 | chr20 | 17937681 | 17949342 | – |
| hsa_piR_018380 | chr1 | 36812280 | 36812307 | – | INTRON | STK40 | chr1 | 36809865 | 36814300 | – |
| hsa_piR_018380 | chr1 | 36812280 | 36812307 | – | INTRON | STK40 | chr1 | 36809865 | 36814300 | – |
| hsa_piR_000045 | chr10 | 70514964 | 70514993 | + | INTRON | CCAR1 | chr10 | 70514584 | 70515126 | + |
| hsa_piR_000045 | chr10 | 70514964 | 70514993 | + | INTRON | CCAR1 | chr10 | 70514584 | 70515126 | + |
| hsa_piR_017184 | chr13 | 45911717 | 45911744 | – | INTRON | TPT1 | chr13 | 45911688 | 45912794 | – |
| hsa_piR_017184 | chr13 | 45911717 | 45911744 | – | INTRON | TPT1 | chr13 | 45911523 | 45912794 | – |
| hsa_piR_004216 | chr15 | 82975509 | 82975539 | + | INTRON | GOLGA6L9 | chr15 | 82805935 | 83101542 | + |
| hsa_piR_008683 | chr11 | 46450671 | 46450696 | – | INTRON | AMBRA1 | chr11 | 46439602 | 46455023 | – |
| hsa_piR_002973 | chr1 | 145438509 | 145438537 | + | INTRON | NBPF10 | chr1 | 145365459 | 146420910 | + |
| hsa_piR_016677 | chr2 | 206218954 | 206218983 | + | INTRON | PARD3B | chr2 | 206166425 | 206265736 | + |
| hsa_piR_011209 | chr12 | 121138849 | 121138880 | + | 3_UTR | MLEC | chr12 | 121134348 | 121139667 | + |
| hsa_piR_011209 | chr12 | 121138849 | 121138880 | + | 3_UTR | MLEC | chr12 | 121134358 | 121139667 | + |
| hsa_piR_017809 | chr1 | 44436748 | 44436774 | + | CDS | DPH2 | chr1 | 44436637 | 44436861 | + |
| hsa_piR_011968 | chr6 | 74227621 | 74227650 | – | CDS | EEF1A1 | chr6 | 74227532 | 74227657 | – |
| hsa_piR_011901 | chr6 | 74228895 | 74228925 | – | CDS | EEF1A1 | chr6 | 74228654 | 74228951 | – |
| hsa_piR_007336 | chr6 | 74228923 | 74228949 | – | CDS | EEF1A1 | chr6 | 74228654 | 74228951 | – |
| hsa_piR_011181 | chr6 | 74229190 | 74229216 | – | CDS | EEF1A1 | chr6 | 74229059 | 74229239 | – |
| hsa_piR_018811 | chr11 | 62339339 | 62339364 | – | CDS | EEF1G | chr11 | 62339309 | 62339373 | – |
| hsa_piR_011398 | chr17 | 7479915 | 7479943 | + | CDS | EIF4A1 | chr17 | 7479841 | 7480010 | + |
| hsa_piR_004150 | chr1 | 7990382 | 7990408 | – | INTRON | TNFRSF9 | chr1 | 7980983 | 7993221 | – |
| hsa_piR_000796 | chr9 | 139908202 | 139908227 | – | INTRON | ABCA2 | chr9 | 139908015 | 139908283 | – |
| hsa_piR_022114 | chr17 | 79478425 | 79478454 | – | CDS | ACTG1 | chr17 | 79478213 | 79478652 | – |
| hsa_piR_001152 | chr2 | 207026645 | 207026674 | + | INTRON | EEF1B2 | chr2 | 207026196 | 207026761 | + |
| hsa_piR_018165 | chr14 | 103804182 | 103804210 | + | INTRON | EIF5 | chr14 | 103803564 | 103804663 | + |
| hsa_piR_019628 | chr17 | 18157227 | 18157253 | – | INTRON | FLII | chr17 | 18157033 | 18157392 | – |
| hsa_piR_001925 | chr11 | 61732284 | 61732314 | – | CDS | FTH1 | chr11 | 61732198 | 61732363 | – |
| hsa_piR_009237 | chr11 | 61732289 | 61732316 | – | CDS | FTH1 | chr11 | 61732198 | 61732363 | – |
| hsa_piR_006613 | chr11 | 61732864 | 61732890 | – | CDS | FTH1 | chr11 | 61732840 | 61732987 | – |
| hsa_piR_022236 | chr6 | 29910611 | 29910640 | + | CDS | HLA–A | chr6 | 29910533 | 29910803 | + |
| hsa_piR_022236 | chr6 | 31239539 | 31239568 | – | CDS | HLA–C | chr6 | 31239375 | 31239645 | – |
| hsa_piR_022236 | chr6 | 29795901 | 29795930 | + | CDS | HLA–G | chr6 | 29795823 | 29796093 | + |
| hsa_piR_015476 | chr16 | 29819002 | 29819031 | + | CDS | MAZ | chr16 | 29818298 | 29819149 | + |
| hsa_piR_021764 | chr1 | 45980634 | 45980661 | – | CDS | PRDX1 | chr1 | 45980544 | 45980667 | – |
| hsa_piR_017184 | chr13 | 45911717 | 45911744 | – | INTRON | TPT1 | chr13 | 45911523 | 45912794 | – |
| hsa_piR_020582 | chr11 | 35001077 | 35001105 | + | INTRON | PDHX | chr11 | 34999729 | 35006116 | + |
| hsa_piR_004506 | chrY | 25342907 | 25342934 | – | INTRON | DAZ1 | chrY | 25338345 | 25344944 | – |
| hsa_piR_000586 | chr5 | 137896735 | 137896762 | – | INTRON | HSPA9 | chr5 | 137895780 | 137897268 | – |
| hsa_piR_001078 | chr6 | 31508903 | 31508931 | – | INTRON | DDX39B | chr6 | 31508441 | 31509726 | – |
| hsa_piR_010155 | chr7 | 45144052 | 45144081 | – | INTRON | TBRG4 | chr7 | 45143855 | 45144136 | – |
| hsa_piR_020364 | chr2 | 51163342 | 51163367 | – | INTRON | NRXN1 | chr2 | 51153093 | 51254639 | – |
| hsa_piR_001168 | chr9 | 5093768 | 5093795 | + | INTRON | JAK2 | chr9 | 5090911 | 5123003 | + |
| hsa_piR_012753 | chr2 | 232321209 | 232321234 | – | INTRON | NCL | chr2 | 232320847 | 232321341 | – |
| hsa_piR_000441 | chr2 | 232325085 | 232325113 | – | INTRON | NCL | chr2 | 232325004 | 232325188 | – |
| hsa_piR_011537 | chr6 | 31321707 | 31321732 | – | 3_UTR | HLA–B | chr6 | 31321648 | 31322073 | – |
| hsa_piR_007150 | chr6 | 31324487 | 31324517 | – | CDS | HLA–B | chr6 | 31324464 | 31324734 | – |
| hsa_piR_019354 | chr11 | 2985098 | 2985123 | – | INTRON | NAP1L4 | chr11 | 2981139 | 2985909 | – |
| hsa_piR_019676 | chr20 | 2634901 | 2634931 | + | INTRON | NOP56 | chr20 | 2634039 | 2635059 | + |
| hsa_piR_012681 | chr20 | 2637585 | 2637613 | + | INTRON | NOP56 | chr20 | 2637541 | 2637726 | + |
| hsa_piR_002973 | chr1 | 145438509 | 145438537 | + | 5_UTR | TXNIP | chr1 | 145438437 | 145438802 | + |
| hsa_piR_001421 | chr17 | 56082521 | 56082549 | – | 3_UTR | SRSF1 | chr17 | 56078279 | 56082766 | – |
| hsa_piR_022794 | chr14 | 70235682 | 70235710 | + | INTRON | SRSF5 | chr14 | 70235613 | 70235898 | + |
| hsa_piR_020466 | chr3 | 131198050 | 131198077 | – | INTRON | MRPL3 | chr3 | 131190123 | 131206523 | – |
| hsa_piR_016975 | chr19 | 49994164 | 49994191 | + | INTRON | RPL13A | chr19 | 49994121 | 49994296 | + |
| hsa_piR_020305 | chr19 | 49994432 | 49994457 | + | INTRON | RPL13A | chr19 | 49994356 | 49994681 | + |
| hsa_piR_020450 | chr9 | 134361109 | 134361138 | + | INTRON | PRRC2B | chr9 | 134360527 | 134361471 | + |
| hsa_piR_020450 | chr9 | 134365930 | 134365959 | + | INTRON | PRRC2B | chr9 | 134363483 | 134366811 | + |
| hsa_piR_020439 | chr19 | 12817303 | 12817331 | – | INTRON | TNPO2 | chr19 | 12817181 | 12817383 | – |
| hsa_piR_007232 | chr5 | 132203289 | 132203316 | + | 3_UTR | UQCRQ | chr5 | 132203274 | 132204536 | + |
| hsa_piR_017033 | chr20 | 17943355 | 17943384 | – | INTRON | SNX5 | chr20 | 17937681 | 17949017 | – |
| hsa_piR_015254 | chr2 | 11724983 | 11725010 | + | INTRON | GREB1 | chr2 | 11720958 | 11725286 | + |
| hsa_piR_012925 | chr1 | 31408589 | 31408618 | – | INTRON | PUM1 | chr1 | 31406189 | 31409489 | – |
| hsa_piR_012925 | chr1 | 31422018 | 31422047 | – | INTRON | PUM1 | chr1 | 31418330 | 31422979 | – |
| hsa_piR_020813 | chr1 | 31441013 | 31441042 | – | INTRON | PUM1 | chr1 | 31440157 | 31441200 | – |
| hsa_piR_017458 | chr1 | 31441015 | 31441041 | – | INTRON | PUM1 | chr1 | 31440157 | 31441200 | – |
| hsa_piR_011209 | chr12 | 121138849 | 121138880 | + | 3_UTR | MLEC | chr12 | 121134348 | 121139667 | + |
| hsa_piR_007635 | chr11 | 46784021 | 46784047 | – | INTRON | CKAP5 | chr11 | 46783743 | 46784176 | – |
| hsa_piR_019368 | chr1 | 155889808 | 155889838 | – | INTRON | KIAA0907 | chr1 | 155887463 | 155891165 | – |
| hsa_piR_020492 | chr1 | 16011004 | 16011029 | + | 5_UTR | PLEKHM2 | chr1 | 16010826 | 16011053 | + |
| hsa_piR_020575 | chr13 | 99639319 | 99639345 | – | INTRON | DOCK9 | chr13 | 99607805 | 99738476 | – |
| hsa_piR_019822 | chr12 | 50610770 | 50610797 | – | INTRON | LIMA1 | chr12 | 50599851 | 50615803 | – |
| hsa_piR_006613 | chr11 | 77445834 | 77445860 | – | INTRON | RSF1 | chr11 | 77436744 | 77451775 | – |
| hsa_piR_008683 | chr11 | 46450671 | 46450696 | – | INTRON | AMBRA1 | chr11 | 46439602 | 46455023 | – |
| hsa_piR_018811 | chr7 | 132720475 | 132720500 | – | INTRON | CHCHD3 | chr7 | 132709387 | 132754901 | – |
| hsa_piR_013350 | chr2 | 234197555 | 234197584 | + | INTRON | ATG16L1 | chr2 | 234191399 | 234198499 | + |
| hsa_piR_018570 | chr16 | 70812150 | 70812181 | – | INTRON | VAC14 | chr16 | 70806075 | 70814692 | – |
| hsa_piR_001312 | chr16 | 70812152 | 70812182 | – | INTRON | VAC14 | chr16 | 70806075 | 70814692 | – |
| hsa_piR_000765 | chr16 | 70812155 | 70812185 | – | INTRON | VAC14 | chr16 | 70806075 | 70814692 | – |
| hsa_piR_018570 | chr16 | 70812978 | 70813009 | – | INTRON | VAC14 | chr16 | 70806075 | 70814692 | – |
| hsa_piR_001312 | chr16 | 70812980 | 70813010 | – | INTRON | VAC14 | chr16 | 70806075 | 70814692 | – |
| hsa_piR_000765 | chr16 | 70812983 | 70813013 | – | INTRON | VAC14 | chr16 | 70806075 | 70814692 | – |
| hsa_piR_017061 | chr17 | 2233581 | 2233612 | – | INTRON | TSR1 | chr17 | 2232769 | 2233808 | – |
| hsa_piR_009016 | chr12 | 123776709 | 123776739 | – | 3_UTR | SBNO1 | chr12 | 123773655 | 123780454 | – |
| hsa_piR_000045 | chr10 | 70514964 | 70514993 | + | INTRON | CCAR1 | chr10 | 70514584 | 70515126 | + |
| hsa_piR_010894 | chr8 | 68497704 | 68497734 | – | INTRON | CPA6 | chr8 | 68430282 | 68536410 | – |
| hsa_piR_004506 | chrY | 25367901 | 25367928 | + | INTRON | DAZ2 | chrY | 25365890 | 25372490 | + |
| hsa_piR_004506 | chrY | 25367901 | 25367928 | + | INTRON | DAZ3 | chrY | 25365890 | 25372490 | + |
| hsa_piR_004506 | chrY | 26982287 | 26982314 | + | INTRON | DAZ3 | chrY | 26980276 | 26997725 | + |
| hsa_piR_004506 | chrY | 25342907 | 25342934 | – | INTRON | DAZ4 | chrY | 25316657 | 25344944 | – |
| hsa_piR_004506 | chrY | 25367901 | 25367928 | + | INTRON | DAZ4 | chrY | 25365890 | 25372490 | + |
| hsa_piR_004506 | chrY | 26982287 | 26982314 | + | INTRON | DAZ4 | chrY | 26980276 | 26997725 | + |
| hsa_piR_019911 | chr14 | 21860382 | 21860413 | – | INTRON | CHD8 | chr14 | 21860105 | 21860665 | – |
| hsa_piR_003728 | chr14 | 21865530 | 21865560 | – | INTRON | CHD8 | chr14 | 21864051 | 21865981 | – |
| hsa_piR_001152 | chr2 | 207026645 | 207026674 | + | INTRON | EEF1B2 | chr2 | 207026196 | 207026761 | + |
| hsa_piR_011181 | chr7 | 144345474 | 144345500 | – | INTRON | TPK1 | chr7 | 144320354 | 144345899 | – |
| hsa_piR_011300 | chr6 | 33240439 | 33240465 | + | CDS | RPS18 | chr6 | 33240404 | 33240503 | + |
| hsa_piR_021214 | chr17 | 8130316 | 8130346 | – | 3_UTR | CTC1 | chr17 | 8128138 | 8131497 | – |
| hsa_piR_020364 | chr17 | 8130357 | 8130382 | – | 3_UTR | CTC1 | chr17 | 8128138 | 8131497 | – |
| hsa_piR_013350 | chr2 | 234197555 | 234197584 | + | INTRON | ATG16L1 | chr2 | 234191399 | 234198499 | + |
| hsa_piR_010155 | chr7 | 45144052 | 45144081 | – | INTRON | TBRG4 | chr7 | 45143042 | 45145039 | – |
| hsa_piR_018380 | chr1 | 36812280 | 36812307 | – | INTRON | STK40 | chr1 | 36809865 | 36814300 | – |
| hsa_piR_015254 | chr2 | 11724983 | 11725010 | + | INTRON | GREB1 | chr2 | 11720958 | 11725286 | + |
| hsa_piR_001207 | chr16 | 89627859 | 89627884 | + | INTRON | RPL13 | chr16 | 89627776 | 89627985 | + |
| hsa_piR_023057 | chr7 | 155569358 | 155569386 | + | 3_UTR | RBM33 | chr7 | 155567735 | 155574179 | + |
| hsa_piR_016677 | chr2 | 206218954 | 206218983 | + | INTRON | PARD3B | chr2 | 206166425 | 206265736 | + |
| hsa_piR_001078 | chr6 | 31508903 | 31508931 | – | INTRON | DDX39B | chr6 | 31508311 | 31509726 | – |
| hsa_piR_004987 | chr15 | 45490847 | 45490877 | – | INTRON | SHF | chr15 | 45470504 | 45490969 | – |
| hsa_piR_004987 | chr15 | 45492654 | 45492684 | – | INTRON | SHF | chr15 | 45491318 | 45492924 | – |
| hsa_piR_020008 | chr3 | 160665449 | 160665479 | + | INTRON | PPM1L | chr3 | 160474495 | 160679523 | + |
| hsa_piR_015254 | chr2 | 11724983 | 11725010 | + | INTRON | GREB1 | chr2 | 11720958 | 11725286 | + |
| hsa_piR_017033 | chr20 | 17943355 | 17943384 | – | INTRON | SNX5 | chr20 | 17937681 | 17949017 | – |
| hsa_piR_016677 | chr2 | 206218954 | 206218983 | + | INTRON | PARD3B | chr2 | 206166425 | 206265736 | + |
| hsa_piR_020499 | chr19 | 36538015 | 36538044 | – | INTRON | THAP8 | chr19 | 36531038 | 36545036 | – |
| hsa_piR_014620 | chr5 | 93905174 | 93905200 | – | INTRON | KIAA0825 | chr5 | 93872832 | 93918137 | – |
| hsa_piR_009237 | chr2 | 27615741 | 27615768 | – | INTRON | PPM1G | chr2 | 27610025 | 27632169 | – |
| hsa_piR_021764 | chr1 | 45980634 | 45980661 | – | CDS | PRDX1 | chr1 | 45980544 | 45980667 | – |
| hsa_piR_021764 | chr1 | 45980634 | 45980661 | – | CDS | PRDX1 | chr1 | 45980544 | 45980667 | – |
| hsa_piR_018165 | chr14 | 103804182 | 103804210 | + | INTRON | EIF5 | chr14 | 103803564 | 103804663 | + |
| hsa_piR_008488 | chr12 | 119431891 | 119431919 | + | INTRON | SRRM4 | chr12 | 119419818 | 119540040 | + |
| hsa_piR_011968 | chr5 | 43495263 | 43495292 | – | INTRON | C5orf34 | chr5 | 43494703 | 43502473 | – |
| hsa_piR_013350 | chr2 | 234197555 | 234197584 | + | INTRON | ATG16L1 | chr2 | 234191399 | 234198499 | + |
| hsa_piR_010155 | chr7 | 45144052 | 45144081 | – | INTRON | TBRG4 | chr7 | 45143042 | 45145039 | – |
| hsa_piR_016677 | chr2 | 206218954 | 206218983 | + | INTRON | PARD3B | chr2 | 206166425 | 206265736 | + |
| hsa_piR_000796 | chr9 | 139908202 | 139908227 | – | INTRON | ABCA2 | chr9 | 139908015 | 139908283 | – |
| hsa_piR_017791 | chr11 | 75111436 | 75111465 | + | 5_UTR | SNORD15A | chr11 | 75111434 | 75111582 | + |
| hsa_piR_001346 | chr17 | 27049639 | 27049667 | + | 5_UTR | SNORD4A | chr17 | 27049599 | 27049671 | + |
| hsa_piR_000560 | chr17 | 27047601 | 27047631 | + | 5_UTR | SNORD42B | chr17 | 27047567 | 27047634 | + |
| hsa_piR_010541 | chr9 | 136217702 | 136217733 | + | 5_UTR | SNORD36C | chr9 | 136217700 | 136217767 | + |
| hsa_piR_020305 | chr19 | 49994432 | 49994457 | + | 5_UTR | SNORD35A | chr19 | 49994431 | 49994517 | + |
| hsa_piR_016975 | chr19 | 49994164 | 49994191 | + | 5_UTR | SNORD34 | chr19 | 49994163 | 49994229 | + |
| hsa_piR_020814 | chr22 | 39709882 | 39709913 | – | 3_UTR | SNORD83B | chr22 | 39709823 | 39709916 | – |
| hsa_piR_002732 | chr22 | 39709884 | 39709911 | – | 3_UTR | SNORD83B | chr22 | 39709823 | 39709916 | – |
| hsa_piR_020815 | chr15 | 25227142 | 25227171 | + | 5_UTR | SNORD107 | chr15 | 25227140 | 25227215 | + |
| hsa_piR_001101 | chr1 | 45244100 | 45244127 | + | 5_UTR | SNORD38B | chr1 | 45244061 | 45244130 | + |
| hsa_piR_009237 | chr2 | 27615741 | 27615768 | – | 3_UTR | FTH1P3 | chr2 | 27615489 | 27616443 | – |
| hsa_piR_017104 | chr6 | 133137966 | 133137994 | + | 5_UTR | SNORD100 | chr6 | 133137940 | 133138016 | + |
| hsa_piR_005271 | chr6 | 133137967 | 133137992 | + | 5_UTR | SNORD100 | chr6 | 133137940 | 133138016 | + |
| hsa_piR_013306 | chr8 | 56986429 | 56986460 | – | 3_UTR | SNORD54 | chr8 | 56986397 | 56986460 | – |
| hsa_piR_000552 | chr22 | 39715057 | 39715084 | – | 3_UTR | SNORD43 | chr22 | 39715056 | 39715118 | – |
| hsa_piR_019050 | chr15 | 66795150 | 66795177 | – | 3_UTR | SNORD16 | chr15 | 66795148 | 66795248 | – |
| hsa_piR_001207 | chr16 | 89627859 | 89627884 | + | 5_UTR | SNORD68 | chr16 | 89627837 | 89627909 | + |
| hsa_piR_017178 | chr18 | 47017687 | 47017717 | – | 3_UTR | SNORD58A | chr18 | 47017652 | 47017717 | – |
| hsa_piR_017194 | chr18 | 47018068 | 47018099 | – | 3_UTR | SNORD58B | chr18 | 47018033 | 47018099 | – |
| hsa_piR_016664 | chr17 | 37009222 | 37009248 | – | 3_UTR | SNORA21 | chr17 | 37009115 | 37009248 | – |
| hsa_piR_001205 | chr11 | 8705774 | 8705805 | + | 5_UTR | SNORA3A | chr11 | 8705773 | 8705903 | + |
| hsa_piR_001152 | chr2 | 207026645 | 207026674 | + | 5_UTR | SNORD51 | chr2 | 207026604 | 207026674 | + |
| hsa_piR_012681 | chr20 | 2637585 | 2637613 | + | 5_UTR | SNORD57 | chr20 | 2637584 | 2637656 | + |
| hsa_piR_001159 | chr17 | 16343391 | 16343420 | + | 5_UTR | SNORD49A | chr17 | 16343349 | 16343420 | + |
| hsa_piR_020439 | chr19 | 12817303 | 12817331 | – | 3_UTR | SNORD41 | chr19 | 12817262 | 12817332 | – |
| hsa_piR_012753 | chr2 | 232321209 | 232321234 | – | 3_UTR | SNORD20 | chr2 | 232321154 | 232321234 | – |
| hsa_piR_000586 | chr5 | 137896735 | 137896762 | – | 3_UTR | SNORD63 | chr5 | 137896731 | 137896799 | – |
| hsa_piR_003728 | chr14 | 21865530 | 21865560 | – | 3_UTR | SNORD8 | chr14 | 21865451 | 21865560 | – |
| hsa_piR_010155 | chr7 | 45144052 | 45144081 | – | 3_UTR | SNORA5A | chr7 | 45143947 | 45144081 | – |
| hsa_piR_017184 | chr13 | 45911717 | 45911744 | – | 3_UTR | SNORA31 | chr13 | 45911614 | 45911744 | – |
| hsa_piR_019354 | chr11 | 2985098 | 2985123 | – | 3_UTR | SNORA54 | chr11 | 2985000 | 2985123 | – |
| hsa_piR_020466 | chr3 | 131198050 | 131198077 | – | 3_UTR | SNORA58 | chr3 | 131197841 | 131198077 | – |
| hsa_piR_020619 | chr1 | 28906276 | 28906307 | – | 3_UTR | SNORA61 | chr1 | 28906275 | 28906405 | – |
| hsa_piR_013350 | chr2 | 234197555 | 234197584 | + | 5_UTR | SCARNA6 | chr2 | 234197321 | 234197587 | + |
| hsa_piR_001042 | chr11 | 93464672 | 93464701 | – | 3_UTR | SNORD6 | chr11 | 93464668 | 93464739 | – |
| hsa_piR_017033 | chr20 | 17943355 | 17943384 | – | 3_UTR | SNORD17 | chr20 | 17943352 | 17943589 | – |
| hsa_piR_007635 | chr11 | 46784021 | 46784047 | – | 3_UTR | SNORD67 | chr11 | 46783938 | 46784049 | – |
| hsa_piR_002158 | chr16 | 71792312 | 71792340 | – | 3_UTR | SNORD71 | chr16 | 71792304 | 71792390 | – |
| hsa_piR_001078 | chr6 | 31508903 | 31508931 | – | 3_UTR | SNORD84 | chr6 | 31508877 | 31508955 | – |
| hsa_piR_020813 | chr1 | 31441013 | 31441042 | – | 3_UTR | SNORD103C | chr1 | 31441009 | 31441084 | – |
| hsa_piR_017458 | chr1 | 31441015 | 31441041 | – | 3_UTR | SNORD103C | chr1 | 31441009 | 31441084 | – |
| hsa_piR_019420 | chr2 | 101889482 | 101889508 | – | 3_UTR | SNORD89 | chr2 | 101889397 | 101889511 | – |
| hsa_piR_017061 | chr17 | 2233581 | 2233612 | – | 3_UTR | SNORD91A | chr17 | 2233572 | 2233664 | – |
| hsa_piR_000045 | chr10 | 70514964 | 70514993 | + | 5_UTR | SNORD98 | chr10 | 70514928 | 70514995 | + |
| hsa_piR_019676 | chr20 | 2634901 | 2634931 | + | 5_UTR | SNORD110 | chr20 | 2634857 | 2634932 | + |
| hsa_piR_019201 | chr14 | 101416172 | 101416202 | + | 5_UTR | SNORD114–1 | chr14 | 101416169 | 101416240 | + |
| hsa_piR_019224 | chr14 | 101416207 | 101416237 | + | 5_UTR | SNORD114–1 | chr14 | 101416169 | 101416240 | + |
| hsa_piR_016963 | chr14 | 101416209 | 101416236 | + | 5_UTR | SNORD114–1 | chr14 | 101416169 | 101416240 | + |
| hsa_piR_019166 | chr14 | 101419688 | 101419718 | + | 5_UTR | SNORD114–3 | chr14 | 101419685 | 101419759 | + |
| hsa_piR_019574 | chr14 | 101449265 | 101449295 | + | 5_UTR | SNORD114–22 | chr14 | 101449262 | 101449333 | + |
| hsa_piR_019102 | chr14 | 101450250 | 101450279 | + | 5_UTR | SNORD114–23 | chr14 | 101450212 | 101450283 | + |
| hsa_piR_001179 | chr14 | 101411989 | 101412015 | + | 5_UTR | SNORD113–9 | chr14 | 101411985 | 101412056 | + |
| hsa_piR_018292 | chr18 | 47015648 | 47015678 | – | 3_UTR | SNORD58C | chr18 | 47015604 | 47015694 | – |
| hsa_piR_012925 | chr1 | 31408589 | 31408618 | – | 3_UTR | SNORD103A | chr1 | 31408535 | 31408623 | – |
| hsa_piR_012925 | chr1 | 31422018 | 31422047 | – | 3_UTR | SNORD103A | chr1 | 31421964 | 31422052 | – |
| hsa_piR_000801 | chr17 | 74557719 | 74557750 | + | 5_UTR | SNORD1A | chr17 | 74557714 | 74557788 | + |
| hsa_piR_020657 | chr17 | 74557195 | 74557226 | + | 5_UTR | SNORD1B | chr17 | 74557189 | 74557275 | + |
| hsa_piR_018780 | chr17 | 74557242 | 74557269 | + | 5_UTR | SNORD1B | chr17 | 74557189 | 74557275 | + |
| hsa_piR_000441 | chr2 | 232325085 | 232325113 | – | 3_UTR | SNORD82 | chr2 | 232325078 | 232325153 | – |
| hsa_piR_020345 | chr19 | 48421765 | 48421791 | + | 5_UTR | SNAR–A1 | chr19 | 48421685 | 48421807 | + |
| hsa_piR_020345 | chr19 | 48437517 | 48437543 | + | 5_UTR | SNAR–A1 | chr19 | 48437437 | 48437559 | + |
| hsa_piR_020345 | chr19 | 48421765 | 48421791 | + | 5_UTR | SNAR–A2 | chr19 | 48421685 | 48421807 | + |
| hsa_piR_020345 | chr19 | 48437517 | 48437543 | + | 5_UTR | SNAR–A2 | chr19 | 48437437 | 48437559 | + |
| hsa_piR_020345 | chr19 | 48411014 | 48411040 | + | 5_UTR | SNAR–A12 | chr19 | 48410934 | 48411056 | + |
| hsa_piR_020345 | chr19 | 48448258 | 48448284 | + | 5_UTR | SNAR–A12 | chr19 | 48448178 | 48448300 | + |
| hsa_piR_020815 | chr15 | 25227142 | 25227171 | + | 5_UTR | PWARSN | chr15 | 25227140 | 25228937 | + |
| hsa_piR_020619 | chr1 | 28906276 | 28906307 | – | INTRON | SNHG12 | chr1 | 28906099 | 28906423 | – |
| hsa_piR_019167 | chr14 | 101364259 | 101364286 | + | INTRON | MEG8 | chr14 | 101361376 | 101365293 | + |
| hsa_piR_020345 | chr19 | 48427115 | 48427141 | + | 5_UTR | SNAR–A3 | chr19 | 48427035 | 48427156 | + |
| hsa_piR_020345 | chr19 | 50595761 | 50595787 | – | 3_UTR | SNAR–A3 | chr19 | 50595745 | 50595866 | – |
| hsa_piR_020345 | chr19 | 50601098 | 50601124 | – | 3_UTR | SNAR–A3 | chr19 | 50601082 | 50601203 | – |
| hsa_piR_020345 | chr19 | 50604163 | 50604189 | – | 3_UTR | SNAR–A3 | chr19 | 50604147 | 50604268 | – |
| hsa_piR_020345 | chr19 | 50607227 | 50607253 | – | 3_UTR | SNAR–A3 | chr19 | 50607211 | 50607332 | – |
| hsa_piR_020345 | chr19 | 50610286 | 50610312 | – | 3_UTR | SNAR–A3 | chr19 | 50610270 | 50610391 | – |
| hsa_piR_020345 | chr19 | 50615640 | 50615666 | – | 3_UTR | SNAR–A3 | chr19 | 50615624 | 50615745 | – |
| hsa_piR_020345 | chr19 | 50620992 | 50621018 | – | 3_UTR | SNAR–A3 | chr19 | 50620976 | 50621097 | – |
| hsa_piR_020345 | chr19 | 50626346 | 50626372 | – | 3_UTR | SNAR–A3 | chr19 | 50626330 | 50626451 | – |
| hsa_piR_020345 | chr19 | 50631674 | 50631700 | – | 3_UTR | SNAR–A3 | chr19 | 50631658 | 50631779 | – |
| hsa_piR_020345 | chr19 | 48427115 | 48427141 | + | 5_UTR | SNAR–A4 | chr19 | 48427035 | 48427156 | + |
| hsa_piR_020345 | chr19 | 50595761 | 50595787 | – | 3_UTR | SNAR–A4 | chr19 | 50595745 | 50595866 | – |
| hsa_piR_020345 | chr19 | 50601098 | 50601124 | – | 3_UTR | SNAR–A4 | chr19 | 50601082 | 50601203 | – |
| hsa_piR_020345 | chr19 | 50604163 | 50604189 | – | 3_UTR | SNAR–A4 | chr19 | 50604147 | 50604268 | – |
| hsa_piR_020345 | chr19 | 50607227 | 50607253 | – | 3_UTR | SNAR–A4 | chr19 | 50607211 | 50607332 | – |
| hsa_piR_020345 | chr19 | 50610286 | 50610312 | – | 3_UTR | SNAR–A4 | chr19 | 50610270 | 50610391 | – |
| hsa_piR_020345 | chr19 | 50615640 | 50615666 | – | 3_UTR | SNAR–A4 | chr19 | 50615624 | 50615745 | – |
| hsa_piR_020345 | chr19 | 50620992 | 50621018 | – | 3_UTR | SNAR–A4 | chr19 | 50620976 | 50621097 | – |
| hsa_piR_020345 | chr19 | 50626346 | 50626372 | – | 3_UTR | SNAR–A4 | chr19 | 50626330 | 50626451 | – |
| hsa_piR_020345 | chr19 | 50631674 | 50631700 | – | 3_UTR | SNAR–A4 | chr19 | 50631658 | 50631779 | – |
| hsa_piR_020345 | chr19 | 48411014 | 48411040 | + | 5_UTR | SNAR–A13 | chr19 | 48410934 | 48411056 | + |
| hsa_piR_020345 | chr19 | 48448258 | 48448284 | + | 5_UTR | SNAR–A13 | chr19 | 48448178 | 48448300 | + |
| hsa_piR_020345 | chr19 | 48427115 | 48427141 | + | 5_UTR | SNAR–A5 | chr19 | 48427035 | 48427156 | + |
| hsa_piR_020345 | chr19 | 50595761 | 50595787 | – | 3_UTR | SNAR–A5 | chr19 | 50595745 | 50595866 | – |
| hsa_piR_020345 | chr19 | 50601098 | 50601124 | – | 3_UTR | SNAR–A5 | chr19 | 50601082 | 50601203 | – |
| hsa_piR_020345 | chr19 | 50604163 | 50604189 | – | 3_UTR | SNAR–A5 | chr19 | 50604147 | 50604268 | – |
| hsa_piR_020345 | chr19 | 50607227 | 50607253 | – | 3_UTR | SNAR–A5 | chr19 | 50607211 | 50607332 | – |
| hsa_piR_020345 | chr19 | 50610286 | 50610312 | – | 3_UTR | SNAR–A5 | chr19 | 50610270 | 50610391 | – |
| hsa_piR_020345 | chr19 | 50615640 | 50615666 | – | 3_UTR | SNAR–A5 | chr19 | 50615624 | 50615745 | – |
| hsa_piR_020345 | chr19 | 50620992 | 50621018 | – | 3_UTR | SNAR–A5 | chr19 | 50620976 | 50621097 | – |
| hsa_piR_020345 | chr19 | 50626346 | 50626372 | – | 3_UTR | SNAR–A5 | chr19 | 50626330 | 50626451 | – |
| hsa_piR_020345 | chr19 | 50631674 | 50631700 | – | 3_UTR | SNAR–A5 | chr19 | 50631658 | 50631779 | – |
| hsa_piR_020345 | chr19 | 48427115 | 48427141 | + | 5_UTR | SNAR–A7 | chr19 | 48427035 | 48427156 | + |
| hsa_piR_020345 | chr19 | 50595761 | 50595787 | – | 3_UTR | SNAR–A7 | chr19 | 50595745 | 50595866 | – |
| hsa_piR_020345 | chr19 | 50601098 | 50601124 | – | 3_UTR | SNAR–A7 | chr19 | 50601082 | 50601203 | – |
| hsa_piR_020345 | chr19 | 50604163 | 50604189 | – | 3_UTR | SNAR–A7 | chr19 | 50604147 | 50604268 | – |
| hsa_piR_020345 | chr19 | 50607227 | 50607253 | – | 3_UTR | SNAR–A7 | chr19 | 50607211 | 50607332 | – |
| hsa_piR_020345 | chr19 | 50610286 | 50610312 | – | 3_UTR | SNAR–A7 | chr19 | 50610270 | 50610391 | – |
| hsa_piR_020345 | chr19 | 50615640 | 50615666 | – | 3_UTR | SNAR–A7 | chr19 | 50615624 | 50615745 | – |
| hsa_piR_020345 | chr19 | 50620992 | 50621018 | – | 3_UTR | SNAR–A7 | chr19 | 50620976 | 50621097 | – |
| hsa_piR_020345 | chr19 | 50626346 | 50626372 | – | 3_UTR | SNAR–A7 | chr19 | 50626330 | 50626451 | – |
| hsa_piR_020345 | chr19 | 50631674 | 50631700 | – | 3_UTR | SNAR–A7 | chr19 | 50631658 | 50631779 | – |
| hsa_piR_020345 | chr19 | 48427115 | 48427141 | + | 5_UTR | SNAR–A11 | chr19 | 48427035 | 48427156 | + |
| hsa_piR_020345 | chr19 | 50595761 | 50595787 | – | 3_UTR | SNAR–A11 | chr19 | 50595745 | 50595866 | – |
| hsa_piR_020345 | chr19 | 50601098 | 50601124 | – | 3_UTR | SNAR–A11 | chr19 | 50601082 | 50601203 | – |
| hsa_piR_020345 | chr19 | 50604163 | 50604189 | – | 3_UTR | SNAR–A11 | chr19 | 50604147 | 50604268 | – |
| hsa_piR_020345 | chr19 | 50607227 | 50607253 | – | 3_UTR | SNAR–A11 | chr19 | 50607211 | 50607332 | – |
| hsa_piR_020345 | chr19 | 50610286 | 50610312 | – | 3_UTR | SNAR–A11 | chr19 | 50610270 | 50610391 | – |
| hsa_piR_020345 | chr19 | 50615640 | 50615666 | – | 3_UTR | SNAR–A11 | chr19 | 50615624 | 50615745 | – |
| hsa_piR_020345 | chr19 | 50620992 | 50621018 | – | 3_UTR | SNAR–A11 | chr19 | 50620976 | 50621097 | – |
| hsa_piR_020345 | chr19 | 50626346 | 50626372 | – | 3_UTR | SNAR–A11 | chr19 | 50626330 | 50626451 | – |
| hsa_piR_020345 | chr19 | 50631674 | 50631700 | – | 3_UTR | SNAR–A11 | chr19 | 50631658 | 50631779 | – |
| hsa_piR_020345 | chr19 | 48427115 | 48427141 | + | 5_UTR | SNAR–A9 | chr19 | 48427035 | 48427156 | + |
| hsa_piR_020345 | chr19 | 50595761 | 50595787 | – | 3_UTR | SNAR–A9 | chr19 | 50595745 | 50595866 | – |
| hsa_piR_020345 | chr19 | 50601098 | 50601124 | – | 3_UTR | SNAR–A9 | chr19 | 50601082 | 50601203 | – |
| hsa_piR_020345 | chr19 | 50604163 | 50604189 | – | 3_UTR | SNAR–A9 | chr19 | 50604147 | 50604268 | – |
| hsa_piR_020345 | chr19 | 50607227 | 50607253 | – | 3_UTR | SNAR–A9 | chr19 | 50607211 | 50607332 | – |
| hsa_piR_020345 | chr19 | 50610286 | 50610312 | – | 3_UTR | SNAR–A9 | chr19 | 50610270 | 50610391 | – |
| hsa_piR_020345 | chr19 | 50615640 | 50615666 | – | 3_UTR | SNAR–A9 | chr19 | 50615624 | 50615745 | – |
| hsa_piR_020345 | chr19 | 50620992 | 50621018 | – | 3_UTR | SNAR–A9 | chr19 | 50620976 | 50621097 | – |
| hsa_piR_020345 | chr19 | 50626346 | 50626372 | – | 3_UTR | SNAR–A9 | chr19 | 50626330 | 50626451 | – |
| hsa_piR_020345 | chr19 | 50631674 | 50631700 | – | 3_UTR | SNAR–A9 | chr19 | 50631658 | 50631779 | – |
| hsa_piR_020345 | chr19 | 48427115 | 48427141 | + | 5_UTR | SNAR–A6 | chr19 | 48427035 | 48427156 | + |
| hsa_piR_020345 | chr19 | 50595761 | 50595787 | – | 3_UTR | SNAR–A6 | chr19 | 50595745 | 50595866 | – |
| hsa_piR_020345 | chr19 | 50601098 | 50601124 | – | 3_UTR | SNAR–A6 | chr19 | 50601082 | 50601203 | – |
| hsa_piR_020345 | chr19 | 50604163 | 50604189 | – | 3_UTR | SNAR–A6 | chr19 | 50604147 | 50604268 | – |
| hsa_piR_020345 | chr19 | 50607227 | 50607253 | – | 3_UTR | SNAR–A6 | chr19 | 50607211 | 50607332 | – |
| hsa_piR_020345 | chr19 | 50610286 | 50610312 | – | 3_UTR | SNAR–A6 | chr19 | 50610270 | 50610391 | – |
| hsa_piR_020345 | chr19 | 50615640 | 50615666 | – | 3_UTR | SNAR–A6 | chr19 | 50615624 | 50615745 | – |
| hsa_piR_020345 | chr19 | 50620992 | 50621018 | – | 3_UTR | SNAR–A6 | chr19 | 50620976 | 50621097 | – |
| hsa_piR_020345 | chr19 | 50626346 | 50626372 | – | 3_UTR | SNAR–A6 | chr19 | 50626330 | 50626451 | – |
| hsa_piR_020345 | chr19 | 50631674 | 50631700 | – | 3_UTR | SNAR–A6 | chr19 | 50631658 | 50631779 | – |
| hsa_piR_020345 | chr19 | 48427115 | 48427141 | + | 5_UTR | SNAR–A8 | chr19 | 48427035 | 48427156 | + |
| hsa_piR_020345 | chr19 | 50595761 | 50595787 | – | 3_UTR | SNAR–A8 | chr19 | 50595745 | 50595866 | – |
| hsa_piR_020345 | chr19 | 50601098 | 50601124 | – | 3_UTR | SNAR–A8 | chr19 | 50601082 | 50601203 | – |
| hsa_piR_020345 | chr19 | 50604163 | 50604189 | – | 3_UTR | SNAR–A8 | chr19 | 50604147 | 50604268 | – |
| hsa_piR_020345 | chr19 | 50607227 | 50607253 | – | 3_UTR | SNAR–A8 | chr19 | 50607211 | 50607332 | – |
| hsa_piR_020345 | chr19 | 50610286 | 50610312 | – | 3_UTR | SNAR–A8 | chr19 | 50610270 | 50610391 | – |
| hsa_piR_020345 | chr19 | 50615640 | 50615666 | – | 3_UTR | SNAR–A8 | chr19 | 50615624 | 50615745 | – |
| hsa_piR_020345 | chr19 | 50620992 | 50621018 | – | 3_UTR | SNAR–A8 | chr19 | 50620976 | 50621097 | – |
| hsa_piR_020345 | chr19 | 50626346 | 50626372 | – | 3_UTR | SNAR–A8 | chr19 | 50626330 | 50626451 | – |
| hsa_piR_020345 | chr19 | 50631674 | 50631700 | – | 3_UTR | SNAR–A8 | chr19 | 50631658 | 50631779 | – |
| hsa_piR_020345 | chr19 | 48427115 | 48427141 | + | 5_UTR | SNAR–A10 | chr19 | 48427035 | 48427156 | + |
| hsa_piR_020345 | chr19 | 50595761 | 50595787 | – | 3_UTR | SNAR–A10 | chr19 | 50595745 | 50595866 | – |
| hsa_piR_020345 | chr19 | 50601098 | 50601124 | – | 3_UTR | SNAR–A10 | chr19 | 50601082 | 50601203 | – |
| hsa_piR_020345 | chr19 | 50604163 | 50604189 | – | 3_UTR | SNAR–A10 | chr19 | 50604147 | 50604268 | – |
| hsa_piR_020345 | chr19 | 50607227 | 50607253 | – | 3_UTR | SNAR–A10 | chr19 | 50607211 | 50607332 | – |
| hsa_piR_020345 | chr19 | 50610286 | 50610312 | – | 3_UTR | SNAR–A10 | chr19 | 50610270 | 50610391 | – |
| hsa_piR_020345 | chr19 | 50615640 | 50615666 | – | 3_UTR | SNAR–A10 | chr19 | 50615624 | 50615745 | – |
| hsa_piR_020345 | chr19 | 50620992 | 50621018 | – | 3_UTR | SNAR–A10 | chr19 | 50620976 | 50621097 | – |
| hsa_piR_020345 | chr19 | 50626346 | 50626372 | – | 3_UTR | SNAR–A10 | chr19 | 50626330 | 50626451 | – |
| hsa_piR_020345 | chr19 | 50631674 | 50631700 | – | 3_UTR | SNAR–A10 | chr19 | 50631658 | 50631779 | – |
| hsa_piR_020345 | chr19 | 48427115 | 48427141 | + | 5_UTR | SNAR–A14 | chr19 | 48427035 | 48427156 | + |
| hsa_piR_020345 | chr19 | 50595761 | 50595787 | – | 3_UTR | SNAR–A14 | chr19 | 50595745 | 50595866 | – |
| hsa_piR_020345 | chr19 | 50601098 | 50601124 | – | 3_UTR | SNAR–A14 | chr19 | 50601082 | 50601203 | – |
| hsa_piR_020345 | chr19 | 50604163 | 50604189 | – | 3_UTR | SNAR–A14 | chr19 | 50604147 | 50604268 | – |
| hsa_piR_020345 | chr19 | 50607227 | 50607253 | – | 3_UTR | SNAR–A14 | chr19 | 50607211 | 50607332 | – |
| hsa_piR_020345 | chr19 | 50610286 | 50610312 | – | 3_UTR | SNAR–A14 | chr19 | 50610270 | 50610391 | – |
| hsa_piR_020345 | chr19 | 50615640 | 50615666 | – | 3_UTR | SNAR–A14 | chr19 | 50615624 | 50615745 | – |
| hsa_piR_020345 | chr19 | 50620992 | 50621018 | – | 3_UTR | SNAR–A14 | chr19 | 50620976 | 50621097 | – |
| hsa_piR_020345 | chr19 | 50626346 | 50626372 | – | 3_UTR | SNAR–A14 | chr19 | 50626330 | 50626451 | – |
| hsa_piR_020345 | chr19 | 50631674 | 50631700 | – | 3_UTR | SNAR–A14 | chr19 | 50631658 | 50631779 | – |
| hsa_piR_016975 | chr19 | 49994164 | 49994191 | + | INTRON | RPL13AP5 | chr19 | 49994121 | 49994296 | + |
| hsa_piR_020305 | chr19 | 49994432 | 49994457 | + | INTRON | RPL13AP5 | chr19 | 49994356 | 49994681 | + |
| hsa_piR_004216 | chr15 | 82764337 | 82764367 | – | 3_UTR | GOLGA2P10 | chr15 | 82763612 | 82765622 | – |
| hsa_piR_004216 | chr15 | 82975509 | 82975539 | + | 5_UTR | GOLGA2P10 | chr15 | 82974255 | 82976263 | + |
| hsa_piR_004216 | chr15 | 83140923 | 83140953 | – | 3_UTR | GOLGA2P10 | chr15 | 83140198 | 83142206 | – |
| hsa_piR_004216 | chr15 | 84868322 | 84868352 | – | 3_UTR | GOLGA2P7 | chr15 | 84867599 | 84869960 | – |
| hsa_piR_001159 | chr17 | 16343391 | 16343420 | + | INTRON | LRRC75A–AS1 | chr17 | 16342728 | 16343498 | + |
| hsa_piR_001159 | chr17 | 16343391 | 16343420 | + | INTRON | LRRC75A–AS1 | chr17 | 16342728 | 16343498 | + |
| hsa_piR_001159 | chr17 | 16343391 | 16343420 | + | INTRON | LRRC75A–AS1 | chr17 | 16343017 | 16343498 | + |
| hsa_piR_001159 | chr17 | 16343391 | 16343420 | + | INTRON | LRRC75A–AS1 | chr17 | 16343017 | 16343498 | + |
| hsa_piR_001159 | chr17 | 16343391 | 16343420 | + | INTRON | LRRC75A–AS1 | chr17 | 16343017 | 16343498 | + |
| hsa_piR_001159 | chr17 | 16343391 | 16343420 | + | INTRON | LRRC75A–AS1 | chr17 | 16343017 | 16343498 | + |
| hsa_piR_001159 | chr17 | 16343391 | 16343420 | + | INTRON | LRRC75A–AS1 | chr17 | 16343017 | 16343498 | + |
| hsa_piR_001159 | chr17 | 16343391 | 16343420 | + | INTRON | LRRC75A–AS1 | chr17 | 16343017 | 16343498 | + |
| hsa_piR_001159 | chr17 | 16343391 | 16343420 | + | INTRON | LRRC75A–AS1 | chr17 | 16342728 | 16343498 | + |
| hsa_piR_001159 | chr17 | 16343391 | 16343420 | + | INTRON | LRRC75A–AS1 | chr17 | 16343017 | 16343498 | + |
| hsa_piR_001159 | chr17 | 16343391 | 16343420 | + | INTRON | LRRC75A–AS1 | chr17 | 16343017 | 16343498 | + |
| hsa_piR_001159 | chr17 | 16343391 | 16343420 | + | INTRON | LRRC75A–AS1 | chr17 | 16343017 | 16343498 | + |
| hsa_piR_001159 | chr17 | 16343391 | 16343420 | + | INTRON | LRRC75A–AS1 | chr17 | 16343017 | 16343498 | + |
| hsa_piR_001159 | chr17 | 16343391 | 16343420 | + | INTRON | LRRC75A–AS1 | chr17 | 16342728 | 16343498 | + |
| hsa_piR_001159 | chr17 | 16343391 | 16343420 | + | INTRON | LRRC75A–AS1 | chr17 | 16342728 | 16343498 | + |
| hsa_piR_001159 | chr17 | 16343391 | 16343420 | + | INTRON | LRRC75A–AS1 | chr17 | 16342728 | 16343498 | + |
| hsa_piR_001159 | chr17 | 16343391 | 16343420 | + | INTRON | LRRC75A–AS1 | chr17 | 16342728 | 16343498 | + |
| hsa_piR_001159 | chr17 | 16343391 | 16343420 | + | INTRON | LRRC75A–AS1 | chr17 | 16342728 | 16343498 | + |
| hsa_piR_001159 | chr17 | 16343391 | 16343420 | + | INTRON | LRRC75A–AS1 | chr17 | 16342728 | 16343498 | + |
| hsa_piR_001159 | chr17 | 16343391 | 16343420 | + | INTRON | LRRC75A–AS1 | chr17 | 16342728 | 16343498 | + |
| hsa_piR_001159 | chr17 | 16343391 | 16343420 | + | INTRON | LRRC75A–AS1 | chr17 | 16342728 | 16343498 | + |
| hsa_piR_001159 | chr17 | 16343391 | 16343420 | + | INTRON | LRRC75A–AS1 | chr17 | 16342728 | 16343498 | + |
| hsa_piR_001159 | chr17 | 16343391 | 16343420 | + | INTRON | LRRC75A–AS1 | chr17 | 16343017 | 16343498 | + |
| hsa_piR_019676 | chr20 | 2634901 | 2634931 | + | INTRON | NOP56 | chr20 | 2634039 | 2635059 | + |
| hsa_piR_012681 | chr20 | 2637585 | 2637613 | + | INTRON | NOP56 | chr20 | 2637541 | 2637726 | + |
| hsa_piR_012925 | chr1 | 31408589 | 31408618 | – | 3_UTR | SNORD103B | chr1 | 31408535 | 31408623 | – |
| hsa_piR_012925 | chr1 | 31422018 | 31422047 | – | 3_UTR | SNORD103B | chr1 | 31421964 | 31422052 | – |
| hsa_piR_004216 | chr15 | 82764337 | 82764367 | – | 3_UTR | GOLGA2P10 | chr15 | 82763612 | 82765954 | – |
| hsa_piR_004216 | chr15 | 82975509 | 82975539 | + | 5_UTR | GOLGA2P10 | chr15 | 82973923 | 82976263 | + |
| hsa_piR_004216 | chr15 | 83140923 | 83140953 | – | 3_UTR | GOLGA2P10 | chr15 | 83140198 | 83142538 | – |
| hsa_piR_020362 | chrX | 3756466 | 3756491 | – | INTRON | LOC389906 | chrX | 3747433 | 3761381 | – |
| hsa_piR_001421 | chr17 | 56082521 | 56082549 | – | 3_UTR | SRSF1 | chr17 | 56082283 | 56083334 | – |
| hsa_piR_022114 | chr17 | 79478425 | 79478454 | – | 3_UTR | ACTG1 | chr17 | 79478213 | 79478652 | – |
| hsa_piR_001078 | chr6 | 31508903 | 31508931 | – | INTRON | DDX39B | chr6 | 31508441 | 31509726 | – |
| hsa_piR_001078 | chr6 | 31508903 | 31508931 | – | INTRON | ATP6V1G2–DDX39B | chr6 | 31508441 | 31509726 | – |
| hsa_piR_011398 | chr17 | 7479915 | 7479943 | + | 5_UTR | SENP3–EIF4A1 | chr17 | 7479841 | 7480010 | + |
| hsa_piR_006613 | chr1 | 228823234 | 228823260 | + | INTRON | RHOU | chr1 | 228780779 | 228873419 | + |
| hsa_piR_020657 | chr17 | 74557195 | 74557226 | + | INTRON | SNHG16 | chr17 | 74555125 | 74557369 | + |
| hsa_piR_018780 | chr17 | 74557242 | 74557269 | + | INTRON | SNHG16 | chr17 | 74555125 | 74557369 | + |
| hsa_piR_000801 | chr17 | 74557719 | 74557750 | + | INTRON | SNHG16 | chr17 | 74557484 | 74559200 | + |
| hsa_piR_020657 | chr17 | 74557195 | 74557226 | + | INTRON | SNHG16 | chr17 | 74555125 | 74557369 | + |
| hsa_piR_018780 | chr17 | 74557242 | 74557269 | + | INTRON | SNHG16 | chr17 | 74555125 | 74557369 | + |
| hsa_piR_000801 | chr17 | 74557719 | 74557750 | + | INTRON | SNHG16 | chr17 | 74557480 | 74559200 | + |
| hsa_piR_020657 | chr17 | 74557195 | 74557226 | + | INTRON | SNHG16 | chr17 | 74553939 | 74557369 | + |
| hsa_piR_018780 | chr17 | 74557242 | 74557269 | + | INTRON | SNHG16 | chr17 | 74553939 | 74557369 | + |
| hsa_piR_000801 | chr17 | 74557719 | 74557750 | + | INTRON | SNHG16 | chr17 | 74557484 | 74559200 | + |
| hsa_piR_020657 | chr17 | 74557195 | 74557226 | + | INTRON | SNHG16 | chr17 | 74553939 | 74557369 | + |
| hsa_piR_018780 | chr17 | 74557242 | 74557269 | + | INTRON | SNHG16 | chr17 | 74553939 | 74557369 | + |
| hsa_piR_000801 | chr17 | 74557719 | 74557750 | + | INTRON | SNHG16 | chr17 | 74557480 | 74559200 | + |
| hsa_piR_001159 | chr17 | 16343391 | 16343420 | + | INTRON | LRRC75A–AS1 | chr17 | 16342707 | 16343498 | + |
| hsa_piR_001159 | chr17 | 16343391 | 16343420 | + | INTRON | LRRC75A–AS1 | chr17 | 16343017 | 16343424 | + |
| hsa_piR_001159 | chr17 | 16343391 | 16343420 | + | INTRON | LRRC75A–AS1 | chr17 | 16342728 | 16343498 | + |
| hsa_piR_001159 | chr17 | 16343391 | 16343420 | + | INTRON | LRRC75A–AS1 | chr17 | 16342728 | 16343498 | + |
| hsa_piR_001159 | chr17 | 16343391 | 16343420 | + | INTRON | LRRC75A–AS1 | chr17 | 16342374 | 16343498 | + |
| hsa_piR_001159 | chr17 | 16343391 | 16343420 | + | INTRON | LRRC75A–AS1 | chr17 | 16343017 | 16343498 | + |
| hsa_piR_001159 | chr17 | 16343391 | 16343420 | + | INTRON | LRRC75A–AS1 | chr17 | 16342728 | 16343498 | + |
| hsa_piR_001159 | chr17 | 16343391 | 16343420 | + | INTRON | LRRC75A–AS1 | chr17 | 16343017 | 16343498 | + |
| hsa_piR_001159 | chr17 | 16343391 | 16343420 | + | INTRON | LRRC75A–AS1 | chr17 | 16342728 | 16343424 | + |
| hsa_piR_021214 | chr17 | 8130316 | 8130346 | – | 3_UTR | CTC1 | chr17 | 8128138 | 8131637 | – |
| hsa_piR_020364 | chr17 | 8130357 | 8130382 | – | 3_UTR | CTC1 | chr17 | 8128138 | 8131637 | – |
| hsa_piR_000925 | chr19 | 49121048 | 49121076 | – | 3_UTR | RPL18 | chr19 | 49121047 | 49121134 | – |
| hsa_piR_016975 | chr19 | 49994164 | 49994191 | + | INTRON | RPL13A | chr19 | 49994121 | 49994296 | + |
| hsa_piR_020305 | chr19 | 49994432 | 49994457 | + | INTRON | RPL13A | chr19 | 49994356 | 49994681 | + |
| hsa_piR_015476 | chr16 | 29819002 | 29819031 | + | INTRON | MAZ | chr16 | 29818214 | 29819550 | + |
| hsa_piR_004987 | chr1 | 147774888 | 147774918 | – | INTRON | NBPF8 | chr1 | 147576928 | 148006225 | – |
| hsa_piR_004987 | chr1 | 147774888 | 147774918 | – | INTRON | NBPF8 | chr1 | 147576928 | 148006225 | – |
| hsa_piR_004216 | chr15 | 82975509 | 82975539 | + | 5_UTR | LOC727751 | chr15 | 82973923 | 82976258 | + |
| hsa_piR_004216 | chr15 | 83140923 | 83140953 | – | 3_UTR | LOC727751 | chr15 | 83140203 | 83142538 | – |
| hsa_piR_004216 | chr15 | 82975509 | 82975539 | + | 5_UTR | LOC727751 | chr15 | 82974255 | 82976258 | + |
| hsa_piR_004216 | chr15 | 83140923 | 83140953 | – | 3_UTR | LOC727751 | chr15 | 83140203 | 83142206 | – |
| hsa_piR_004216 | chr15 | 82764337 | 82764367 | – | 3_UTR | GOLGA2P10 | chr15 | 82763612 | 82765622 | – |
| hsa_piR_004216 | chr15 | 82975509 | 82975539 | + | 5_UTR | GOLGA2P10 | chr15 | 82974255 | 82976263 | + |
| hsa_piR_004216 | chr15 | 83140923 | 83140953 | – | 3_UTR | GOLGA2P10 | chr15 | 83140198 | 83142206 | – |
| hsa_piR_020809 | chr1 | 148598324 | 148598353 | – | INTRON | NBPF25P | chr1 | 148346793 | 149108982 | – |
| hsa_piR_021190 | chr1 | 148598325 | 148598351 | – | INTRON | NBPF25P | chr1 | 148346793 | 149108982 | – |
| hsa_piR_016240 | chr1 | 148598326 | 148598354 | – | INTRON | NBPF25P | chr1 | 148346793 | 149108982 | – |
| hsa_piR_016946 | chr1 | 148598333 | 148598358 | – | INTRON | NBPF25P | chr1 | 148346793 | 149108982 | – |
| hsa_piR_020809 | chr1 | 148760366 | 148760395 | – | INTRON | NBPF25P | chr1 | 148346793 | 149108982 | – |
| hsa_piR_021190 | chr1 | 148760367 | 148760393 | – | INTRON | NBPF25P | chr1 | 148346793 | 149108982 | – |
| hsa_piR_016240 | chr1 | 148760368 | 148760396 | – | INTRON | NBPF25P | chr1 | 148346793 | 149108982 | – |
| hsa_piR_016946 | chr1 | 148760375 | 148760400 | – | INTRON | NBPF25P | chr1 | 148346793 | 149108982 | – |
| hsa_piR_000045 | chr10 | 70514964 | 70514993 | + | INTRON | CCAR1 | chr10 | 70514584 | 70515126 | + |
| hsa_piR_004993 | chr5 | 180618687 | 180618713 | – | INTRON | LOC102577426 | chr5 | 180618345 | 180618741 | – |
| hsa_piR_019574 | chr14 | 101449265 | 101449295 | + | INTRON | SNHG24 | chr14 | 101448922 | 101449797 | + |
| hsa_piR_019102 | chr14 | 101450250 | 101450279 | + | INTRON | SNHG24 | chr14 | 101449879 | 101450684 | + |
| hsa_piR_004309 | chr2 | 47507898 | 47507929 | – | INTRON | LOC101927043 | chr2 | 47448462 | 47572039 | – |
| hsa_piR_017723 | chr2 | 47507901 | 47507930 | – | INTRON | LOC101927043 | chr2 | 47448462 | 47572039 | – |
| hsa_piR_004309 | chr2 | 47507898 | 47507929 | – | INTRON | LOC101927043 | chr2 | 47426682 | 47559411 | – |
| hsa_piR_017723 | chr2 | 47507901 | 47507930 | – | INTRON | LOC101927043 | chr2 | 47426682 | 47559411 | – |
| hsa_piR_004216 | chr15 | 82975509 | 82975539 | + | INTRON | GOLGA6L17P | chr15 | 82808795 | 83193062 | + |
