# Supplementary material for: Specific patterns of PIWI-interacting small noncoding RNA expression in dysplastic liver nodules and hepatocellular carcinoma
Source: Oncotarget. 2016 Jul 13;7(34):54650–61. doi: 10.18632/oncotarget.10567 (PMC5342370; doi:10.18632/oncotarget.10567)
Supplement: Supplementary file 4 [file oncotarget-07-54650-s004.docx]

| Supplementary Table S4: piRNAs differential expressed between cirrhosis and HCC tissues | | | | | |
| --- | --- | --- | --- | --- | --- |
|  | **HCC_vs_Cirrhosis** | | | **HCC_A_vs_Cirr** | **HCC_B_vs_Cirr** |
| **piRNA ID** | ***p*Value** | **FDR** | **FC** | **FC** | **FC** |
| hsa_piR_016946 | 0.00010 | 0.00388 | –17.60 | –50.90 | –19.55 |
| hsa_piR_021190 | 0.00016 | 0.00388 | –16.00 | –39.67 | –22.09 |
| hsa_piR_008033 | 0.00570 | 0.02726 | –15.88 | –21.28 | –20.14 |
| hsa_piR_007336 | 0.00792 | 0.03449 | –15.70 | –20.24 | –20.24 |
| hsa_piR_020809 | 0.00016 | 0.00388 | –12.77 | –43.92 | –10.96 |
| hsa_piR_016240 | 0.00016 | 0.00388 | –12.39 | –40.53 | –10.87 |
| hsa_piR_001042 | 0.00027 | 0.00487 | –10.59 | –20.48 | –8.90 |
| hsa_piR_011901 | 0.00291 | 0.02015 | –8.49 | –31.49 | –3.47 |
| hsa_piR_005018 | 0.00018 | 0.00388 | –6.99 | –6.72 | –10.11 |
| hsa_piR_001152 | 0.00012 | 0.00388 | –6.49 | –15.11 | –5.31 |
| hsa_piR_017295 | 0.00961 | 0.03845 | –6.47 | –8.00 | –10.08 |
| hsa_piR_019102 | 0.00824 | 0.03510 | –5.83 | –15.40 | –1.88 |
| hsa_piR_005019 | 0.00961 | 0.03845 | –4.41 | –4.27 | –6.36 |
| hsa_piR_021214 | 0.00294 | 0.02015 | –4.22 | –9.86 | –4.14 |
| hsa_piR_012681 | 0.00100 | 0.01158 | –4.16 | –13.99 | –3.33 |
| hsa_piR_019201 | 0.00781 | 0.03449 | –3.96 | –15.02 | –2.87 |
| hsa_piR_019168 | 0.00053 | 0.00868 | –3.91 | –11.70 | –2.55 |
| hsa_piR_019420 | 0.00002 | 0.00385 | –3.41 | –7.04 | –3.01 |
| hsa_piR_017791 | 0.00089 | 0.01086 | –2.63 | –3.98 | –2.45 |
| hsa_piR_017061 | 0.00164 | 0.01458 | –2.53 | –9.51 | –1.63 |
| hsa_piR_020450 | 0.00145 | 0.01354 | –2.47 | –7.52 | –1.92 |
| hsa_piR_017033 | 0.00184 | 0.01506 | –2.11 | –9.65 | –1.42 |
| hsa_piR_014620 | 0.01064 | 0.04169 | –1.95 | –3.66 | –1.75 |
| hsa_piR_020814 | 0.00114 | 0.01238 | –1.81 | –2.24 | –1.63 |
| hsa_piR_018165 | 0.01416 | 0.04752 | 1.84 | 1.27 | 6.71 |
| hsa_piR_020499 | 0.01173 | 0.04346 | 1.98 | 1.72 | 4.76 |
| hsa_piR_020829 | 0.00089 | 0.01086 | 2.30 | 2.24 | 3.25 |
| hsa_piR_019951 | 0.00294 | 0.02015 | 2.33 | 4.10 | 2.49 |
| hsa_piR_020365 | 0.00570 | 0.02726 | 2.52 | 1.85 | 4.98 |
| hsa_piR_020305 | 0.00309 | 0.02015 | 2.58 | 1.46 | 6.34 |
| hsa_piR_001205 | 0.01297 | 0.04461 | 2.81 | 1.55 | 5.98 |
| hsa_piR_000823 | 0.00512 | 0.02573 | 2.87 | 1.90 | 5.70 |
| hsa_piR_019824 | 0.00395 | 0.02215 | 2.90 | 1.54 | 2.21 |
| hsa_piR_023057 | 0.01211 | 0.04396 | 3.04 | 1.00 | 8.74 |
| hsa_piR_004987 | 0.00368 | 0.02121 | 3.06 | 2.38 | 4.98 |
| hsa_piR_017724 | 0.00868 | 0.03619 | 3.16 | 11.76 | 1.65 |
| hsa_piR_019914 | 0.01431 | 0.04752 | 3.34 | 2.33 | 6.33 |
| hsa_piR_004309 | 0.01175 | 0.04346 | 3.58 | 9.15 | 1.39 |
| hsa_piR_003728 | 0.01259 | 0.04461 | 3.58 | 2.29 | 4.55 |
| hsa_piR_020498 | 0.00018 | 0.00388 | 3.86 | 2.75 | 8.74 |
| hsa_piR_013306 | 0.01162 | 0.04346 | 4.74 | 1.64 | 7.08 |
| hsa_piR_016970 | 0.00634 | 0.02961 | 5.01 | 10.93 | 2.58 |
| hsa_piR_010894 | 0.00329 | 0.02015 | 5.21 | 2.96 | 7.01 |
| hsa_piR_020363 | 0.00303 | 0.02015 | 5.25 | 1.86 | 14.63 |
| hsa_piR_020362 | 0.00303 | 0.02015 | 5.29 | 1.86 | 14.63 |
| hsa_piR_020657 | 0.00756 | 0.03448 | 5.60 | 5.08 | 5.84 |
| hsa_piR_016975 | 0.00014 | 0.00388 | 5.94 | 5.48 | 14.84 |
| hsa_piR_020364 | 0.00339 | 0.02015 | 6.28 | 2.74 | 17.20 |
| hsa_piR_009051 | 0.00069 | 0.01037 | 7.04 | 3.08 | 12.99 |
| hsa_piR_020828 | 0.00088 | 0.01086 | 7.28 | 2.42 | 11.01 |
| hsa_piR_000291 | 0.00145 | 0.01354 | 7.73 | 8.15 | 65.03 |
| hsa_piR_016664 | 0.00410 | 0.02232 | 8.66 | 9.04 | 62.94 |
| hsa_piR_020009 | 0.01297 | 0.04461 | 9.98 | 3.28 | 18.36 |
| hsa_piR_001170 | 0.00174 | 0.01479 | 11.85 | 2.86 | 17.00 |
| hsa_piR_005076 | 0.00497 | 0.02566 | 12.07 | 5.92 | 21.85 |
| hsa_piR_019368 | 0.00141 | 0.01354 | 12.95 | 3.41 | 18.67 |
| hsa_piR_019822 | 0.00334 | 0.02015 | 20.74 | 13.23 | 39.17 |
| hsa_piR_010155 | 0.00020 | 0.00388 | 25.76 | 13.03 | 76.05 |
| The 58 piRNAs differential expressed between cirrhosis and HCC tissues, for each piRNA is reported p Value, FDR and FC (in green FC ≤–1.5 and red FC ≥ 1.5). Furthermore, FC between cirrhosis and HCC samples clustered in different clade (A, B Figure 1C) are calculated separately. | | | | | |
