# Supplementary material for: Specific patterns of PIWI-interacting small noncoding RNA expression in dysplastic liver nodules and hepatocellular carcinoma
Source: Oncotarget. 2016 Jul 13;7(34):54650–61. doi: 10.18632/oncotarget.10567 (PMC5342370; doi:10.18632/oncotarget.10567)
Supplement: Supplementary file 5 [file oncotarget-07-54650-s005.docx]

| Supplementary Table S6A: piRNA–like expression in cirrhosis and HCC tissues | | |
| --- | --- | --- |
| **piRNA ID** | **Median CIRR** | **Median HCC** |
| piR_LLi_11090 | 49277 | 99104 |
| piR_LLi_6364 | 44378 | 78456 |
| piR_LLi_1982 | 43577 | 22884 |
| piR_LLi_30238 | 33286 | 37183 |
| piR_LLi_14613 | 31829 | 5887 |
| piR_LLi_3740 | 25155 | 4938 |
| piR_LLi_25269 | 23587 | 47007 |
| piR_LLi_21904 | 21036 | 6905 |
| piR_LLi_30564 | 18536 | 53039 |
| piR_LLi_24 | 13593 | 13775 |
| piR_LLi_16037 | 6376 | 33774 |
| piR_LLi_4594 | 5384 | 975 |
| piR_LLi_21177 | 5365 | 6148 |
| piR_LLi_25376 | 5073 | 13031 |
| piR_LLi_11071 | 4740 | 293 |
| piR_LLi_25990 | 4664 | 4699 |
| piR_LLi_27965 | 4487 | 2650 |
| piR_LLi_1844 | 3584 | 1782 |
| piR_LLi_8147 | 3370 | 2333 |
| piR_LLi_26431 | 3224 | 640 |
| piR_LLi_17604 | 2977 | 3268 |
| piR_LLi_11041 | 2753 | 11482 |
| piR_LLi_27644 | 2649 | 3418 |
| piR_LLi_3706 | 2056 | 2356 |
| piR_LLi_2555 | 2014 | 2210 |
| piR_LLi_21958 | 1701 | 531 |
| piR_LLi_11091 | 1627 | 1020 |
| piR_LLi_20856 | 1437 | 13500 |
| piR_LLi_10414 | 1397 | 343 |
| piR_LLi_21240 | 1395 | 2726 |
| piR_LLi_18347 | 1386 | 7217 |
| piR_LLi_11086 | 1318 | 1195 |
| piR_LLi_10379 | 1297 | 839 |
| piR_LLi_29900 | 1269 | 1240 |
| piR_LLi_1970 | 1170 | 3770 |
| piR_LLi_15689 | 1137 | 4734 |
| piR_LLi_19839 | 1104 | 1101 |
| piR_LLi_7967 | 1078 | 1323 |
| piR_LLi_23939 | 1076 | 473 |
| piR_LLi_21856 | 1044 | 1001 |
| piR_LLi_30579 | 1028 | 5873 |
| piR_LLi_27140 | 746 | 1221 |
| piR_LLi_10747 | 727 | 891 |
| piR_LLi_26065 | 704 | 528 |
| piR_LLi_11072 | 685 | 8105 |
| piR_LLi_19055 | 663 | 460 |
| piR_LLi_11084 | 585 | 1673 |
| piR_LLi_11958 | 581 | 3180 |
| piR_LLi_27814 | 580 | 93 |
| piR_LLi_1152 | 567 | 1129 |
| piR_LLi_7316 | 542 | 176 |
| piR_LLi_59 | 536 | 298 |
| piR_LLi_582 | 514 | 0 |
| piR_LLi_7579 | 458 | 226 |
| piR_LLi_25648 | 440 | 932 |
| piR_LLi_1841 | 426 | 49 |
| piR_LLi_19358 | 423 | 43 |
| piR_LLi_589 | 418 | 3320 |
| piR_LLi_5920 | 408 | 2625 |
| piR_LLi_1675 | 398 | 420 |
| piR_LLi_11020 | 398 | 178 |
| piR_LLi_13847 | 394 | 1143 |
| piR_LLi_28890 | 381 | 2100 |
| piR_LLi_16316 | 379 | 614 |
| piR_LLi_14132 | 378 | 818 |
| piR_LLi_3762 | 367 | 4315 |
| piR_LLi_14302 | 349 | 153 |
| piR_LLi_12035 | 345 | 50 |
| piR_LLi_23031 | 342 | 210 |
| piR_LLi_27495 | 324 | 764 |
| piR_LLi_22205 | 293 | 437 |
| piR_LLi_30389 | 286 | 371 |
| piR_LLi_2559 | 284 | 266 |
| piR_LLi_16724 | 283 | 0 |
| piR_LLi_2564 | 273 | 273 |
| piR_LLi_16880 | 266 | 283 |
| piR_LLi_14784 | 252 | 1049 |
| piR_LLi_30732 | 248 | 3354 |
| piR_LLi_19836 | 248 | 914 |
| piR_LLi_667 | 216 | 414 |
| piR_LLi_1736 | 200 | 554 |
| piR_LLi_1975 | 193 | 873 |
| piR_LLi_14165 | 189 | 0 |
| piR_LLi_4613 | 186 | 0 |
| piR_LLi_17146 | 170 | 1433 |
| piR_LLi_26811 | 163 | 779 |
| piR_LLi_25604 | 157 | 0 |
| piR_LLi_27414 | 130 | 670 |
| piR_LLi_17912 | 126 | 0 |
| piR_LLi_3158 | 116 | 471 |
| piR_LLi_28226 | 110 | 2898 |
| piR_LLi_11030 | 110 | 0 |
| piR_LLi_5407 | 80 | 105 |
| piR_LLi_14703 | 70 | 691 |
| piR_LLi_2558 | 70 | 177 |
| piR_LLi_14720 | 60 | 329 |
| piR_LLi_25374 | 60 | 289 |
| piR_LLi_30118 | 40 | 388 |
| piR_LLi_3157 | 30 | 0 |
| piR_LLi_1977 | 0 | 4665 |
| piR_LLi_6296 | 0 | 1289 |
| piR_LLi_11378 | 0 | 966 |
| piR_LLi_54 | 0 | 847 |
| piR_LLi_5762 | 0 | 719 |
| piR_LLi_72 | 0 | 662 |
| piR_LLi_29425 | 0 | 606 |
| piR_LLi_24763 | 0 | 604 |
| piR_LLi_13491 | 0 | 563 |
| piR_LLi_752 | 0 | 552 |
| piR_LLi_9693 | 0 | 549 |
| piR_LLi_2581 | 0 | 525 |
| piR_LLi_27190 | 0 | 517 |
| piR_LLi_30517 | 0 | 477 |
| piR_LLi_23766 | 0 | 469 |
| piR_LLi_29876 | 0 | 460 |
| piR_LLi_17603 | 0 | 431 |
| piR_LLi_30580 | 0 | 425 |
| piR_LLi_22184 | 0 | 413 |
| piR_LLi_18601 | 0 | 411 |
| piR_LLi_8111 | 0 | 402 |
| piR_LLi_14590 | 0 | 401 |
| piR_LLi_15226 | 0 | 397 |
| piR_LLi_29854 | 0 | 388 |
| piR_LLi_4699 | 0 | 387 |
| piR_LLi_12844 | 0 | 382 |
| piR_LLi_9694 | 0 | 379 |
| piR_LLi_17229 | 0 | 371 |
| piR_LLi_13629 | 0 | 370 |
| piR_LLi_13839 | 0 | 361 |
| piR_LLi_7 | 0 | 352 |
| piR_LLi_28197 | 0 | 349 |
| piR_LLi_14969 | 0 | 342 |
| piR_LLi_24736 | 0 | 320 |
| piR_LLi_29551 | 0 | 317 |
| piR_LLi_13788 | 0 | 274 |
| piR_LLi_26197 | 0 | 270 |
| piR_LLi_25265 | 0 | 270 |
| piR_LLi_11094 | 0 | 264 |
| piR_LLi_18697 | 0 | 253 |
| piR_LLi_5195 | 0 | 244 |
| piR_LLi_17148 | 0 | 239 |
| piR_LLi_2847 | 0 | 238 |
| piR_LLi_20985 | 0 | 234 |
| piR_LLi_24737 | 0 | 226 |
| piR_LLi_9589 | 0 | 222 |
| piR_LLi_10267 | 0 | 220 |
| piR_LLi_29368 | 0 | 218 |
| piR_LLi_29354 | 0 | 217 |
| piR_LLi_19050 | 0 | 216 |
| piR_LLi_11052 | 0 | 210 |
| piR_LLi_18303 | 0 | 207 |
| piR_LLi_1712 | 0 | 206 |
| piR_LLi_13330 | 0 | 199 |
| piR_LLi_24259 | 0 | 199 |
| piR_LLi_8643 | 0 | 199 |
| piR_LLi_11297 | 0 | 194 |
| piR_LLi_590 | 0 | 188 |
| piR_LLi_6618 | 0 | 188 |
| piR_LLi_10166 | 0 | 187 |
| piR_LLi_27963 | 0 | 187 |
| piR_LLi_9699 | 0 | 186 |
| piR_LLi_4085 | 0 | 186 |
| piR_LLi_8211 | 0 | 182 |
| piR_LLi_4883 | 0 | 182 |
| piR_LLi_20098 | 0 | 171 |
| piR_LLi_12717 | 0 | 165 |
| piR_LLi_7125 | 0 | 163 |
| piR_LLi_25502 | 0 | 162 |
| piR_LLi_10266 | 0 | 162 |
| piR_LLi_5518 | 0 | 161 |
| piR_LLi_27815 | 0 | 160 |
| piR_LLi_13838 | 0 | 160 |
| piR_LLi_30566 | 0 | 157 |
| piR_LLi_24164 | 0 | 155 |
| piR_LLi_1842 | 0 | 152 |
| piR_LLi_30581 | 0 | 148 |
| piR_LLi_25271 | 0 | 144 |
| piR_LLi_9854 | 0 | 137 |
| piR_LLi_2292 | 0 | 135 |
| piR_LLi_4767 | 0 | 134 |
| piR_LLi_26453 | 0 | 132 |
| piR_LLi_25890 | 0 | 132 |
| piR_LLi_22437 | 0 | 132 |
| piR_LLi_14391 | 0 | 126 |
| piR_LLi_4430 | 0 | 115 |
| piR_LLi_15728 | 0 | 110 |
| piR_LLi_24879 | 0 | 104 |
| piR_LLi_11596 | 0 | 104 |
| piR_LLi_24573 | 0 | 95 |
| piR_LLi_27812 | 0 | 94 |
| piR_LLi_17391 | 0 | 86 |
| piR_LLi_17332 | 0 | 82 |
| piR_LLi_30552 | 0 | 79 |
| piR_LLi_3659 | 0 | 72 |
| piR_LLi_11019 | 0 | 68 |
| piR_LLi_1635 | 0 | 66 |
| piR_LLi_13150 | 0 | 65 |
| piR_LLi_5788 | 0 | 58 |
| piR_LLi_2776 | 0 | 56 |
| piR_LLi_18599 | 0 | 53 |
| piR_LLi_24877 | 0 | 52 |
| piR_LLi_4491 | 0 | 50 |
| piR_LLi_7111 | 0 | 50 |
| piR_LLi_43 | 0 | 49 |
| piR_LLi_28783 | 0 | 43 |
| piR_LLi_24416 | 0 | 42 |
| piR_LLi_16039 | 0 | 42 |
| piR_LLi_5876 | 0 | 0 |
| piR_LLi_3064 | 0 | 0 |
| piR_LLi_3931 | 0 | 0 |
| piR_LLi_17190 | 0 | 0 |
| piR_LLi_27753 | 0 | 0 |
| piR_LLi_25661 | 0 | 0 |
| piR_LLi_12334 | 0 | 0 |
| piR_LLi_3443 | 0 | 0 |
| piR_LLi_23419 | 0 | 0 |
| piR_LLi_17627 | 0 | 0 |
| piR_LLi_24151 | 0 | 0 |
| piR_LLi_118 | 0 | 0 |
| piR_LLi_16817 | 0 | 0 |
| piR_LLi_15025 | 0 | 0 |
| piR_LLi_11042 | 0 | 0 |
| piR_LLi_29206 | 0 | 0 |
| piR_LLi_11022 | 0 | 0 |
| piR_LLi_24875 | 0 | 0 |
| piR_LLi_30374 | 0 | 0 |
| piR_LLi_30117 | 0 | 0 |
| piR_LLi_28658 | 0 | 0 |
| piR_LLi_18042 | 0 | 0 |
| piR_LLi_2951 | 0 | 0 |
| piR_LLi_15397 | 0 | 0 |
| piR_LLi_10740 | 0 | 0 |
| piR_LLi_16727 | 0 | 0 |
| piR_LLi_27192 | 0 | 0 |
| piR_LLi_26446 | 0 | 0 |
| piR_LLi_28485 | 0 | 0 |
| piR_LLi_1686 | 0 | 0 |
| piR_LLi_26571 | 0 | 0 |
| piR_LLi_19431 | 0 | 0 |
| piR_LLi_30561 | 0 | 0 |
| piR_LLi_13836 | 0 | 0 |
| piR_LLi_2794 | 0 | 0 |
| piR_LLi_6887 | 0 | 0 |
| piR_LLi_30709 | 0 | 0 |
| piR_LLi_22221 | 0 | 0 |
| piR_LLi_17931 | 0 | 0 |
| piR_LLi_22204 | 0 | 0 |
| piR_LLi_25275 | 0 | 0 |
| piR_LLi_27286 | 0 | 0 |
| piR_LLi_9801 | 0 | 0 |
| piR_LLi_2552 | 0 | 0 |
| piR_LLi_2554 | 0 | 0 |
| piR_LLi_30612 | 0 | 0 |
| piR_LLi_1987 | 0 | 0 |
| piR_LLi_30619 | 0 | 0 |
| piR_LLi_29570 | 0 | 0 |
| piR_LLi_29741 | 0 | 0 |
| piR_LLi_25715 | 0 | 0 |
| piR_LLi_1845 | 0 | 0 |
| piR_LLi_14833 | 0 | 0 |
| piR_LLi_16636 | 0 | 0 |
| piR_LLi_14392 | 0 | 0 |
| piR_LLi_29667 | 0 | 0 |
| piR_LLi_18651 | 0 | 0 |
| piR_LLi_7090 | 0 | 0 |
| piR_LLi_25024 | 0 | 0 |
| piR_LLi_11080 | 0 | 0 |
| piR_LLi_11081 | 0 | 0 |
| piR_LLi_5264 | 0 | 0 |
| piR_LLi_26064 | 0 | 0 |
| piR_LLi_25273 | 0 | 0 |
| piR_LLi_21681 | 0 | 0 |
| piR_LLi_9857 | 0 | 0 |
| piR_LLi_5921 | 0 | 0 |
| piR_LLi_30525 | 0 | 0 |
| piR_LLi_18840 | 0 | 0 |
| piR_LLi_1983 | 0 | 0 |
| piR_LLi_30518 | 0 | 0 |
| piR_LLi_23011 | 0 | 0 |
| piR_LLi_19264 | 0 | 0 |
| piR_LLi_26268 | 0 | 0 |
| piR_LLi_19518 | 0 | 0 |
| piR_LLi_27586 | 0 | 0 |
| piR_LLi_22311 | 0 | 0 |
| piR_LLi_25984 | 0 | 0 |
| piR_LLi_22668 | 0 | 0 |
| piR_LLi_20356 | 0 | 0 |
| piR_LLi_6469 | 0 | 0 |
| piR_LLi_19508 | 0 | 0 |
| piR_LLi_20474 | 0 | 0 |
| piR_LLi_479 | 0 | 0 |
| piR_LLi_10745 | 0 | 0 |
| piR_LLi_7377 | 0 | 0 |
| piR_LLi_21512 | 0 | 0 |
| piR_LLi_3162 | 0 | 0 |
| piR_LLi_4413 | 0 | 0 |
| piR_LLi_10255 | 0 | 0 |
| piR_LLi_1309 | 0 | 0 |
| piR_LLi_1687 | 0 | 0 |
| piR_LLi_809 | 0 | 0 |
| piR_LLi_11079 | 0 | 0 |
| piR_LLi_19825 | 0 | 0 |
| piR_LLi_30737 | 0 | 0 |
| piR_LLi_23633 | 0 | 0 |
| piR_LLi_30662 | 0 | 0 |
| piR_LLi_4431 | 0 | 0 |
| piR_LLi_11085 | 0 | 0 |
| piR_LLi_3159 | 0 | 0 |
| piR_LLi_1767 | 0 | 0 |
| piR_LLi_1584 | 0 | 0 |
| piR_LLi_2557 | 0 | 0 |
| piR_LLi_9853 | 0 | 0 |
| piR_LLi_30596 | 0 | 0 |
| piR_LLi_22595 | 0 | 0 |
| piR_LLi_35 | 0 | 0 |
| piR_LLi_11054 | 0 | 0 |
| piR_LLi_22088 | 0 | 0 |
| piR_LLi_2204 | 0 | 0 |
| piR_LLi_21498 | 0 | 0 |
| piR_LLi_8338 | 0 | 0 |
| piR_LLi_11059 | 0 | 0 |
| piR_LLi_2287 | 0 | 0 |
| piR_LLi_1976 | 0 | 0 |
| piR_LLi_24574 | 0 | 0 |
| piR_LLi_44 | 0 | 0 |
| piR_LLi_18179 | 0 | 0 |
| piR_LLi_24350 | 0 | 0 |
| piR_LLi_21753 | 0 | 0 |
| piR_LLi_8224 | 0 | 0 |
| piR_LLi_3492 | 0 | 0 |
| piR_LLi_30521 | 0 | 0 |
| piR_LLi_15846 | 0 | 0 |
| piR_LLi_24396 | 0 | 0 |
| piR_LLi_8095 | 0 | 0 |
| piR_LLi_16652 | 0 | 0 |
| piR_LLi_11056 | 0 | 0 |
| piR_LLi_17322 | 0 | 0 |
| piR_LLi_17188 | 0 | 0 |
| piR_LLi_7385 | 0 | 0 |
| piR_LLi_11176 | 0 | 0 |
| piR_LLi_22237 | 0 | 0 |
| piR_LLi_2565 | 0 | 0 |
| piR_LLi_28232 | 0 | 0 |
| piR_LLi_4429 | 0 | 0 |
| piR_LLi_592 | 0 | 0 |
| piR_LLi_15480 | 0 | 0 |
| piR_LLi_27101 | 0 | 0 |
| piR_LLi_27064 | 0 | 0 |
| piR_LLi_17450 | 0 | 0 |
| piR_LLi_5639 | 0 | 0 |
| piR_LLi_9954 | 0 | 0 |
| piR_LLi_24388 | 0 | 0 |
| piR_LLi_1974 | 0 | 0 |
| piR_LLi_24134 | 0 | 0 |
| piR_LLi_808 | 0 | 0 |
| piR_LLi_4281 | 0 | 0 |
| piR_LLi_30628 | 0 | 0 |
| piR_LLi_6293 | 0 | 0 |
| piR_LLi_22182 | 0 | 0 |
| Expression value (read per million) of 359 piRNA–Like liver detected in cirrhosis and HCC tissues. | | |
