# Supplementary material for: Specific patterns of PIWI-interacting small noncoding RNA expression in dysplastic liver nodules and hepatocellular carcinoma
Source: Oncotarget. 2016 Jul 13;7(34):54650–61. doi: 10.18632/oncotarget.10567 (PMC5342370; doi:10.18632/oncotarget.10567)
Supplement: Supplementary file 6 [file oncotarget-07-54650-s006.docx]

| Supplementary Table S6B: Annotation of liver piRNA–Like | | | | | | | | | | |
| --- | --- | --- | --- | --- | --- | --- | --- | --- | --- | --- |
| **piR_LLi_ID** | **piR_LLi_chr** | **piR_LLi_start** | **piR_LLi_end** | **piR_LLi_strand** | **Gene_Region** | **Gene_name** | **Gene_chr** | **Gene_start** | **Gene_end** | **Gene_strand** |
| piR_LLi_17450 | chr10 | 50713240 | 50713271 | – | INTRON | ERCC6 | chr10 | 50708742 | 50713929 | – |
| piR_LLi_27192 | chr19 | 13007585 | 13007612 | + | INTRON | GCDH | chr19 | 13007235 | 13007723 | + |
| piR_LLi_28783 | chr21 | 38179657 | 38179687 | – | INTRON | HLCS | chr21 | 38139586 | 38269159 | – |
| piR_LLi_7967 | chr4 | 74284963 | 74284991 | + | INTRON | ALB | chr4 | 74284028 | 74285223 | + |
| piR_LLi_17931 | chr10 | 96699613 | 96699640 | + | INTRON | CYP2C9 | chr10 | 96698607 | 96701614 | + |
| piR_LLi_9693 | chr5 | 79946906 | 79946935 | – | INTRON | DHFR | chr5 | 79945313 | 79949826 | – |
| piR_LLi_9694 | chr5 | 79946933 | 79946965 | – | INTRON | DHFR | chr5 | 79945313 | 79949826 | – |
| piR_LLi_9699 | chr5 | 79947460 | 79947495 | – | INTRON | DHFR | chr5 | 79945313 | 79949826 | – |
| piR_LLi_13150 | chr7 | 75573099 | 75573126 | + | INTRON | POR | chr7 | 75544497 | 75583306 | + |
| piR_LLi_25502 | chr17 | 27050476 | 27050510 | + | INTRON | RPL23A | chr17 | 27049917 | 27050588 | + |
| piR_LLi_29354 | chr22 | 46548657 | 46548689 | + | INTRON | PPARA | chr22 | 46547873 | 46594238 | + |
| piR_LLi_28890 | chr21 | 45744008 | 45744043 | + | INTRON | PFKL | chr21 | 45743801 | 45744373 | + |
| piR_LLi_18179 | chr10 | 116239155 | 116239186 | – | INTRON | ABLIM1 | chr10 | 116233715 | 116247716 | – |
| piR_LLi_18179 | chr10 | 116239155 | 116239186 | – | INTRON | ABLIM1 | chr10 | 116233715 | 116245055 | – |
| piR_LLi_24350 | chr16 | 16346162 | 16346197 | + | INTRON | NOMO3 | chr16 | 16345957 | 16346289 | + |
| piR_LLi_25265 | chr17 | 7809381 | 7809411 | + | INTRON | CHD3 | chr17 | 7809307 | 7809870 | + |
| piR_LLi_25265 | chr17 | 7809381 | 7809411 | + | INTRON | CHD3 | chr17 | 7809307 | 7809870 | + |
| piR_LLi_3443 | chr2 | 55479917 | 55479944 | – | INTRON | MTIF2 | chr2 | 55479789 | 55481176 | – |
| piR_LLi_25661 | chr17 | 37881865 | 37881894 | + | INTRON | ERBB2 | chr17 | 37881655 | 37881959 | + |
| piR_LLi_24388 | chr16 | 20463429 | 20463456 | + | INTRON | ACSM2A | chr16 | 20463003 | 20463702 | + |
| piR_LLi_18697 | chr11 | 17096257 | 17096292 | – | INTRON | RPS13 | chr11 | 17096013 | 17096643 | – |
| piR_LLi_14590 | chr8 | 56986396 | 56986430 | – | INTRON | RPS20 | chr8 | 56986327 | 56986618 | – |
| piR_LLi_12035 | chr6 | 142740789 | 142740824 | + | INTRON | ADGRG6 | chr6 | 142738506 | 142740957 | + |
| piR_LLi_12035 | chr6 | 142740789 | 142740824 | + | INTRON | ADGRG6 | chr6 | 142738506 | 142740957 | + |
| piR_LLi_12717 | chr7 | 33600966 | 33600995 | + | INTRON | BBS9 | chr7 | 33573788 | 33644476 | + |
| piR_LLi_12717 | chr7 | 33600966 | 33600995 | + | INTRON | BBS9 | chr7 | 33573788 | 33644476 | + |
| piR_LLi_5264 | chr2 | 236508530 | 236508565 | + | INTRON | AGAP1 | chr2 | 236403493 | 236617822 | + |
| piR_LLi_22668 | chr14 | 70235789 | 70235821 | + | INTRON | SRSF5 | chr14 | 70235613 | 70235898 | + |
| piR_LLi_29667 | chrX | 41078548 | 41078574 | + | INTRON | USP9X | chrX | 41078484 | 41082469 | + |
| piR_LLi_29667 | chrX | 41078548 | 41078574 | + | INTRON | USP9X | chrX | 41078484 | 41082469 | + |
| piR_LLi_12334 | chr6 | 168343921 | 168343950 | + | INTRON | MLLT4 | chr6 | 168343874 | 168344081 | + |
| piR_LLi_589 | chr1 | 28834048 | 28834083 | + | INTRON | RCC1 | chr1 | 28832596 | 28834639 | + |
| piR_LLi_590 | chr1 | 28835068 | 28835103 | + | INTRON | RCC1 | chr1 | 28834672 | 28835341 | + |
| piR_LLi_592 | chr1 | 28835245 | 28835274 | + | INTRON | RCC1 | chr1 | 28834672 | 28835341 | + |
| piR_LLi_20985 | chr12 | 109677885 | 109677914 | + | INTRON | ACACB | chr12 | 109677790 | 109678882 | + |
| piR_LLi_17188 | chr10 | 24813056 | 24813085 | + | INTRON | KIAA1217 | chr10 | 24810858 | 24813251 | + |
| piR_LLi_16724 | chr9 | 131702507 | 131702536 | + | INTRON | PHYHD1 | chr9 | 131700057 | 131702647 | + |
| piR_LLi_16724 | chr9 | 131702507 | 131702536 | + | INTRON | PHYHD1 | chr9 | 131700057 | 131702647 | + |
| piR_LLi_29206 | chr22 | 36891827 | 36891861 | – | INTRON | FOXRED2 | chr22 | 36889850 | 36892013 | – |
| piR_LLi_7377 | chr4 | 6999028 | 6999054 | + | INTRON | TBC1D14 | chr4 | 6998134 | 7000811 | + |
| piR_LLi_7377 | chr4 | 6999028 | 6999054 | + | INTRON | TBC1D14 | chr4 | 6998134 | 7000811 | + |
| piR_LLi_26446 | chr18 | 21407158 | 21407188 | + | INTRON | LAMA3 | chr18 | 21406763 | 21407277 | + |
| piR_LLi_3659 | chr2 | 75739770 | 75739804 | – | INTRON | EVA1A | chr2 | 75720735 | 75745181 | – |
| piR_LLi_5876 | chr3 | 49282204 | 49282235 | + | INTRON | CCDC36 | chr3 | 49282166 | 49292822 | + |
| piR_LLi_16727 | chr9 | 131844912 | 131844939 | + | INTRON | DOLPP1 | chr9 | 131843486 | 131846946 | + |
| piR_LLi_25715 | chr17 | 41105504 | 41105534 | – | INTRON | PTGES3L–AARSD1 | chr17 | 41103911 | 41105740 | – |
| piR_LLi_27190 | chr19 | 12814448 | 12814482 | – | INTRON | TNPO2 | chr19 | 12814340 | 12814527 | – |
| piR_LLi_27190 | chr19 | 12814448 | 12814482 | – | INTRON | TNPO2 | chr19 | 12814340 | 12814527 | – |
| piR_LLi_4613 | chr2 | 171877221 | 171877256 | – | INTRON | TLK1 | chr2 | 171871458 | 171884848 | – |
| piR_LLi_4613 | chr2 | 171877221 | 171877256 | – | INTRON | TLK1 | chr2 | 171871458 | 171884848 | – |
| piR_LLi_14613 | chr8 | 59509872 | 59509902 | – | INTRON | NSMAF | chr8 | 59508218 | 59509945 | – |
| piR_LLi_19358 | chr11 | 74907424 | 74907459 | + | INTRON | SLCO2B1 | chr11 | 74904620 | 74907558 | + |
| piR_LLi_19358 | chr11 | 74907424 | 74907459 | + | INTRON | SLCO2B1 | chr11 | 74904620 | 74907558 | + |
| piR_LLi_9801 | chr5 | 93905171 | 93905206 | – | INTRON | KIAA0825 | chr5 | 93872832 | 93918137 | – |
| piR_LLi_14969 | chr8 | 99159770 | 99159797 | + | INTRON | POP1 | chr8 | 99158911 | 99161042 | + |
| piR_LLi_14969 | chr8 | 99159770 | 99159797 | + | INTRON | POP1 | chr8 | 99158911 | 99161042 | + |
| piR_LLi_14720 | chr8 | 70602297 | 70602328 | – | INTRON | SLCO5A1 | chr8 | 70594578 | 70617265 | – |
| piR_LLi_14720 | chr8 | 70602297 | 70602328 | – | INTRON | SLCO5A1 | chr8 | 70594578 | 70617265 | – |
| piR_LLi_14590 | chr8 | 56986396 | 56986430 | – | INTRON | RPS20 | chr8 | 56986327 | 56986618 | – |
| piR_LLi_6469 | chr3 | 114387142 | 114387172 | – | INTRON | ZBTB20 | chr3 | 114219238 | 114412374 | – |
| piR_LLi_6469 | chr3 | 114387142 | 114387172 | – | INTRON | ZBTB20 | chr3 | 114219238 | 114412374 | – |
| piR_LLi_6469 | chr3 | 114387142 | 114387172 | – | INTRON | ZBTB20 | chr3 | 114219238 | 114412374 | – |
| piR_LLi_15025 | chr8 | 104321271 | 104321302 | + | INTRON | FZD6 | chr8 | 104312512 | 104330817 | + |
| piR_LLi_15025 | chr8 | 104321271 | 104321302 | + | INTRON | FZD6 | chr8 | 104312512 | 104330817 | + |
| piR_LLi_30117 | chrX | 135961356 | 135961389 | – | INTRON | RBMX | chrX | 135961282 | 135961477 | – |
| piR_LLi_30118 | chrX | 135961401 | 135961432 | – | INTRON | RBMX | chrX | 135961282 | 135961477 | – |
| piR_LLi_18303 | chr10 | 126176856 | 126176890 | + | INTRON | LHPP | chr10 | 126172895 | 126176990 | + |
| piR_LLi_22204 | chr14 | 21860308 | 21860342 | – | INTRON | CHD8 | chr14 | 21860105 | 21860665 | – |
| piR_LLi_22205 | chr14 | 21865450 | 21865484 | – | INTRON | CHD8 | chr14 | 21864051 | 21865981 | – |
| piR_LLi_20474 | chr12 | 50850355 | 50850390 | + | INTRON | LARP4 | chr12 | 50848200 | 50856364 | + |
| piR_LLi_20474 | chr12 | 50850355 | 50850390 | + | INTRON | LARP4 | chr12 | 50848200 | 50854917 | + |
| piR_LLi_20474 | chr12 | 50850355 | 50850390 | + | INTRON | LARP4 | chr12 | 50848200 | 50856364 | + |
| piR_LLi_16636 | chr9 | 125918876 | 125918903 | – | INTRON | STRBP | chr9 | 125910149 | 125920290 | – |
| piR_LLi_16316 | chr9 | 97831454 | 97831488 | + | INTRON | C9orf3 | chr9 | 97823092 | 97842975 | + |
| piR_LLi_10266 | chr5 | 138611867 | 138611894 | + | INTRON | MATR3 | chr5 | 138611839 | 138614739 | + |
| piR_LLi_10267 | chr5 | 138611977 | 138612012 | + | INTRON | MATR3 | chr5 | 138611839 | 138614739 | + |
| piR_LLi_14302 | chr8 | 26485549 | 26485584 | + | INTRON | DPYSL2 | chr8 | 26485456 | 26492295 | + |
| piR_LLi_2204 | chr1 | 196885601 | 196885632 | + | INTRON | CFHR4 | chr1 | 196884268 | 196887339 | + |
| piR_LLi_2204 | chr1 | 196885601 | 196885632 | + | INTRON | CFHR4 | chr1 | 196884268 | 196887339 | + |
| piR_LLi_12334 | chr6 | 168343921 | 168343950 | + | INTRON | MLLT4 | chr6 | 168343871 | 168344081 | + |
| piR_LLi_118 | chr1 | 2594162 | 2594191 | – | INTRON | TTC34 | chr1 | 2577002 | 2700153 | – |
| piR_LLi_28783 | chr21 | 38179657 | 38179687 | – | INTRON | HLCS | chr21 | 38139586 | 38269159 | – |
| piR_LLi_28783 | chr21 | 38179657 | 38179687 | – | INTRON | HLCS | chr21 | 38139586 | 38269159 | – |
| piR_LLi_22088 | chr13 | 111273523 | 111273554 | + | INTRON | CARKD | chr13 | 111268121 | 111274562 | + |
| piR_LLi_22088 | chr13 | 111273523 | 111273554 | + | INTRON | CARKD | chr13 | 111267994 | 111274562 | + |
| piR_LLi_22088 | chr13 | 111273523 | 111273554 | + | INTRON | CARKD | chr13 | 111267994 | 111279785 | + |
| piR_LLi_13330 | chr7 | 98479322 | 98479356 | + | INTRON | TRRAP | chr7 | 98478873 | 98479597 | + |
| piR_LLi_14302 | chr8 | 26485549 | 26485584 | + | INTRON | DPYSL2 | chr8 | 26485456 | 26492295 | + |
| piR_LLi_5264 | chr2 | 236508530 | 236508565 | + | INTRON | AGAP1 | chr2 | 236403493 | 236617822 | + |
| piR_LLi_10414 | chr5 | 150434593 | 150434623 | – | INTRON | TNIP1 | chr5 | 150431820 | 150436326 | – |
| piR_LLi_10414 | chr5 | 150434593 | 150434623 | – | INTRON | TNIP1 | chr5 | 150431820 | 150436326 | – |
| piR_LLi_10414 | chr5 | 150434593 | 150434623 | – | INTRON | TNIP1 | chr5 | 150431820 | 150436326 | – |
| piR_LLi_10414 | chr5 | 150434593 | 150434623 | – | INTRON | TNIP1 | chr5 | 150431820 | 150436326 | – |
| piR_LLi_10414 | chr5 | 150434593 | 150434623 | – | INTRON | TNIP1 | chr5 | 150431820 | 150436326 | – |
| piR_LLi_10414 | chr5 | 150434593 | 150434623 | – | INTRON | TNIP1 | chr5 | 150431820 | 150436326 | – |
| piR_LLi_5639 | chr3 | 27474772 | 27474803 | – | INTRON | SLC4A7 | chr3 | 27465643 | 27475406 | – |
| piR_LLi_5639 | chr3 | 27474772 | 27474803 | – | INTRON | SLC4A7 | chr3 | 27465643 | 27475406 | – |
| piR_LLi_10414 | chr5 | 150434593 | 150434623 | – | INTRON | TNIP1 | chr5 | 150431820 | 150436326 | – |
| piR_LLi_10414 | chr5 | 150434593 | 150434623 | – | INTRON | TNIP1 | chr5 | 150431820 | 150436326 | – |
| piR_LLi_10414 | chr5 | 150434593 | 150434623 | – | INTRON | TNIP1 | chr5 | 150431820 | 150436326 | – |
| piR_LLi_25715 | chr17 | 41105504 | 41105534 | – | INTRON | AARSD1 | chr17 | 41103911 | 41105740 | – |
| piR_LLi_12844 | chr7 | 45144051 | 45144082 | – | INTRON | TBRG4 | chr7 | 45143855 | 45144136 | – |
| piR_LLi_24763 | chr16 | 58582503 | 58582538 | – | INTRON | CNOT1 | chr16 | 58581586 | 58583622 | – |
| piR_LLi_27644 | chr19 | 49993872 | 49993906 | + | INTRON | RPL13A | chr19 | 49993833 | 49994035 | + |
| piR_LLi_15728 | chr9 | 19063754 | 19063785 | – | INTRON | HAUS6 | chr9 | 19063578 | 19070216 | – |
| piR_LLi_7125 | chr3 | 184083101 | 184083129 | + | INTRON | POLR2H | chr3 | 184083014 | 184084504 | + |
| piR_LLi_7125 | chr3 | 184083101 | 184083129 | + | INTRON | POLR2H | chr3 | 184083014 | 184084504 | + |
| piR_LLi_7125 | chr3 | 184083101 | 184083129 | + | INTRON | POLR2H | chr3 | 184083014 | 184084504 | + |
| piR_LLi_7125 | chr3 | 184083101 | 184083129 | + | INTRON | POLR2H | chr3 | 184083014 | 184085964 | + |
| piR_LLi_7125 | chr3 | 184083101 | 184083129 | + | INTRON | POLR2H | chr3 | 184083014 | 184085964 | + |
| piR_LLi_10266 | chr5 | 138611867 | 138611894 | + | INTRON | MATR3 | chr5 | 138611839 | 138614015 | + |
| piR_LLi_10267 | chr5 | 138611977 | 138612012 | + | INTRON | MATR3 | chr5 | 138611839 | 138614015 | + |
| piR_LLi_27965 | chr20 | 17943351 | 17943386 | – | INTRON | SNX5 | chr20 | 17937681 | 17949342 | – |
| piR_LLi_15846 | chr9 | 33933115 | 33933148 | – | INTRON | UBAP2 | chr9 | 33932626 | 33933487 | – |
| piR_LLi_15846 | chr9 | 33933115 | 33933148 | – | INTRON | UBAP2 | chr9 | 33932626 | 33933487 | – |
| piR_LLi_17188 | chr10 | 24813056 | 24813085 | + | INTRON | KIAA1217 | chr10 | 24810858 | 24813251 | + |
| piR_LLi_17188 | chr10 | 24813056 | 24813085 | + | INTRON | KIAA1217 | chr10 | 24810858 | 24813251 | + |
| piR_LLi_17188 | chr10 | 24813056 | 24813085 | + | INTRON | KIAA1217 | chr10 | 24810858 | 24813251 | + |
| piR_LLi_17188 | chr10 | 24813056 | 24813085 | + | INTRON | KIAA1217 | chr10 | 24810858 | 24813251 | + |
| piR_LLi_1767 | chr1 | 155723708 | 155723734 | – | INTRON | GON4L | chr1 | 155723205 | 155724165 | – |
| piR_LLi_1767 | chr1 | 155723708 | 155723734 | – | INTRON | GON4L | chr1 | 155723205 | 155724165 | – |
| piR_LLi_1767 | chr1 | 155723708 | 155723734 | – | INTRON | GON4L | chr1 | 155723205 | 155724165 | – |
| piR_LLi_17603 | chr10 | 70496928 | 70496955 | + | INTRON | CCAR1 | chr10 | 70496805 | 70500385 | + |
| piR_LLi_17604 | chr10 | 70514961 | 70514996 | + | INTRON | CCAR1 | chr10 | 70514584 | 70515126 | + |
| piR_LLi_17603 | chr10 | 70496928 | 70496955 | + | INTRON | CCAR1 | chr10 | 70496805 | 70500385 | + |
| piR_LLi_17604 | chr10 | 70514961 | 70514996 | + | INTRON | CCAR1 | chr10 | 70514584 | 70515126 | + |
| piR_LLi_7377 | chr4 | 6999028 | 6999054 | + | INTRON | TBC1D14 | chr4 | 6998134 | 7000811 | + |
| piR_LLi_8147 | chr4 | 94076198 | 94076232 | + | INTRON | GRID2 | chr4 | 94032104 | 94128554 | + |
| piR_LLi_3162 | chr2 | 29355208 | 29355236 | + | INTRON | CLIP4 | chr2 | 29355111 | 29356520 | + |
| piR_LLi_3162 | chr2 | 29355208 | 29355236 | + | INTRON | CLIP4 | chr2 | 29355111 | 29356520 | + |
| piR_LLi_667 | chr1 | 35530868 | 35530895 | + | INTRON | ZMYM1 | chr1 | 35525526 | 35544594 | + |
| piR_LLi_25661 | chr17 | 37881865 | 37881894 | + | INTRON | ERBB2 | chr17 | 37881655 | 37881959 | + |
| piR_LLi_25661 | chr17 | 37881865 | 37881894 | + | INTRON | ERBB2 | chr17 | 37881655 | 37881959 | + |
| piR_LLi_23633 | chr15 | 66639649 | 66639683 | – | INTRON | TIPIN | chr15 | 66633694 | 66641397 | – |
| piR_LLi_18347 | chr10 | 128609375 | 128609406 | + | INTRON | DOCK1 | chr10 | 128594132 | 128768965 | + |
| piR_LLi_9693 | chr5 | 79946906 | 79946935 | – | INTRON | DHFR | chr5 | 79945313 | 79950222 | – |
| piR_LLi_9694 | chr5 | 79946933 | 79946965 | – | INTRON | DHFR | chr5 | 79945313 | 79950222 | – |
| piR_LLi_9699 | chr5 | 79947460 | 79947495 | – | INTRON | DHFR | chr5 | 79945313 | 79950222 | – |
| piR_LLi_9693 | chr5 | 79946906 | 79946935 | – | INTRON | DHFR | chr5 | 79945313 | 79949826 | – |
| piR_LLi_9694 | chr5 | 79946933 | 79946965 | – | INTRON | DHFR | chr5 | 79945313 | 79949826 | – |
| piR_LLi_9699 | chr5 | 79947460 | 79947495 | – | INTRON | DHFR | chr5 | 79945313 | 79949826 | – |
| piR_LLi_12334 | chr6 | 168343921 | 168343950 | + | INTRON | MLLT4 | chr6 | 168343874 | 168344081 | + |
| piR_LLi_808 | chr1 | 45136641 | 45136675 | – | INTRON | TMEM53 | chr1 | 45120881 | 45140002 | – |
| piR_LLi_809 | chr1 | 45136689 | 45136716 | – | INTRON | TMEM53 | chr1 | 45120881 | 45140002 | – |
| piR_LLi_808 | chr1 | 45136641 | 45136675 | – | INTRON | TMEM53 | chr1 | 45125967 | 45140002 | – |
| piR_LLi_809 | chr1 | 45136689 | 45136716 | – | INTRON | TMEM53 | chr1 | 45125967 | 45140002 | – |
| piR_LLi_808 | chr1 | 45136641 | 45136675 | – | INTRON | TMEM53 | chr1 | 45125967 | 45140002 | – |
| piR_LLi_809 | chr1 | 45136689 | 45136716 | – | INTRON | TMEM53 | chr1 | 45125967 | 45140002 | – |
| piR_LLi_1987 | chr1 | 173938453 | 173938481 | – | INTRON | RC3H1 | chr1 | 173934258 | 173939642 | – |
| piR_LLi_1987 | chr1 | 173938453 | 173938481 | – | INTRON | RC3H1 | chr1 | 173934258 | 173939642 | – |
| piR_LLi_1987 | chr1 | 173938453 | 173938481 | – | INTRON | RC3H1 | chr1 | 173934258 | 173939642 | – |
| piR_LLi_10379 | chr5 | 148591345 | 148591378 | + | INTRON | ABLIM3 | chr5 | 148590382 | 148596521 | + |
| piR_LLi_10379 | chr5 | 148591345 | 148591378 | + | INTRON | ABLIM3 | chr5 | 148590382 | 148596521 | + |
| piR_LLi_10379 | chr5 | 148591345 | 148591378 | + | INTRON | ABLIM3 | chr5 | 148590382 | 148596521 | + |
| piR_LLi_10379 | chr5 | 148591345 | 148591378 | + | INTRON | ABLIM3 | chr5 | 148590382 | 148596521 | + |
| piR_LLi_752 | chr1 | 41465807 | 41465838 | + | INTRON | CTPS1 | chr1 | 41463198 | 41466700 | + |
| piR_LLi_20098 | chr12 | 7052994 | 7053023 | + | INTRON | C12orf57 | chr12 | 7052825 | 7053269 | + |
| piR_LLi_20098 | chr12 | 7052994 | 7053023 | + | INTRON | C12orf57 | chr12 | 7052825 | 7053638 | + |
| piR_LLi_20098 | chr12 | 7052994 | 7053023 | + | 5_UTR | C12orf57 | chr12 | 7052979 | 7053284 | + |
| piR_LLi_20098 | chr12 | 7052994 | 7053023 | + | 5_UTR | C12orf57 | chr12 | 7052979 | 7053336 | + |
| piR_LLi_5762 | chr3 | 39449880 | 39449915 | + | INTRON | RPSA | chr3 | 39449277 | 39450096 | + |
| piR_LLi_24388 | chr16 | 20463429 | 20463456 | + | INTRON | ACSM2A | chr16 | 20463003 | 20476838 | + |
| piR_LLi_24388 | chr16 | 20463429 | 20463456 | + | INTRON | ACSM2A | chr16 | 20463003 | 20471428 | + |
| piR_LLi_18347 | chr10 | 128609375 | 128609406 | + | INTRON | DOCK1 | chr10 | 128594132 | 128768965 | + |
| piR_LLi_14302 | chr8 | 26485549 | 26485584 | + | INTRON | DPYSL2 | chr8 | 26485456 | 26492295 | + |
| piR_LLi_8147 | chr4 | 94076198 | 94076232 | + | INTRON | GRID2 | chr4 | 94032104 | 94128554 | + |
| piR_LLi_16880 | chr9 | 139908198 | 139908228 | – | INTRON | ABCA2 | chr9 | 139908015 | 139908283 | – |
| piR_LLi_752 | chr1 | 41465807 | 41465838 | + | INTRON | CTPS1 | chr1 | 41463198 | 41466700 | + |
| piR_LLi_27064 | chr19 | 3982536 | 3982571 | – | INTRON | EEF2 | chr19 | 3982422 | 3982804 | – |
| piR_LLi_21498 | chr13 | 41209625 | 41209655 | – | INTRON | FOXO1 | chr13 | 41134997 | 41239719 | – |
| piR_LLi_30117 | chrX | 135961356 | 135961389 | – | INTRON | RBMX | chrX | 135961282 | 135961477 | – |
| piR_LLi_30118 | chrX | 135961401 | 135961432 | – | INTRON | RBMX | chrX | 135961282 | 135961477 | – |
| piR_LLi_5762 | chr3 | 39449880 | 39449915 | + | INTRON | RPSA | chr3 | 39449277 | 39450096 | + |
| piR_LLi_18179 | chr10 | 116239155 | 116239186 | – | INTRON | ABLIM1 | chr10 | 116233715 | 116247716 | – |
| piR_LLi_3443 | chr2 | 55479917 | 55479944 | – | INTRON | MTIF2 | chr2 | 55479789 | 55481176 | – |
| piR_LLi_28890 | chr21 | 45744008 | 45744043 | + | INTRON | PFKL | chr21 | 45743801 | 45744373 | + |
| piR_LLi_2847 | chr2 | 3302907 | 3302939 | – | INTRON | TSSC1 | chr2 | 3261226 | 3341787 | – |
| piR_LLi_13330 | chr7 | 98479322 | 98479356 | + | INTRON | TRRAP | chr7 | 98478873 | 98479597 | + |
| piR_LLi_15025 | chr8 | 104321271 | 104321302 | + | INTRON | FZD6 | chr8 | 104312512 | 104330817 | + |
| piR_LLi_14613 | chr8 | 59509872 | 59509902 | – | INTRON | NSMAF | chr8 | 59508218 | 59509945 | – |
| piR_LLi_5639 | chr3 | 27474772 | 27474803 | – | INTRON | SLC4A7 | chr3 | 27473160 | 27475406 | – |
| piR_LLi_4594 | chr2 | 169855925 | 169855953 | – | INTRON | ABCB11 | chr2 | 169853232 | 169869781 | – |
| piR_LLi_26268 | chr18 | 3084961 | 3084990 | – | INTRON | MYOM1 | chr18 | 3084025 | 3085042 | – |
| piR_LLi_26197 | chr17 | 80047838 | 80047873 | – | INTRON | FASN | chr17 | 80047602 | 80048250 | – |
| piR_LLi_10255 | chr5 | 137894659 | 137894692 | – | INTRON | HSPA9 | chr5 | 137894346 | 137895552 | – |
| piR_LLi_22311 | chr14 | 32954082 | 32954112 | + | INTRON | AKAP6 | chr14 | 32903023 | 33004759 | + |
| piR_LLi_25661 | chr17 | 37881865 | 37881894 | + | INTRON | ERBB2 | chr17 | 37881655 | 37881959 | + |
| piR_LLi_1152 | chr1 | 76252755 | 76252790 | + | INTRON | RABGGTB | chr1 | 76251959 | 76253181 | + |
| piR_LLi_12844 | chr7 | 45144051 | 45144082 | – | INTRON | TBRG4 | chr7 | 45143855 | 45144136 | – |
| piR_LLi_29354 | chr22 | 46548657 | 46548689 | + | INTRON | PPARA | chr22 | 46547873 | 46572649 | + |
| piR_LLi_5195 | chr2 | 232321206 | 232321235 | – | INTRON | NCL | chr2 | 232320847 | 232321341 | – |
| piR_LLi_24164 | chr16 | 3336165 | 3336194 | + | INTRON | ZNF263 | chr16 | 3336149 | 3338453 | + |
| piR_LLi_25265 | chr17 | 7809381 | 7809411 | + | INTRON | CHD3 | chr17 | 7809307 | 7809870 | + |
| piR_LLi_8224 | chr4 | 103668701 | 103668727 | – | INTRON | MANBA | chr4 | 103647840 | 103681874 | – |
| piR_LLi_10414 | chr5 | 150434593 | 150434623 | – | INTRON | TNIP1 | chr5 | 150431820 | 150436326 | – |
| piR_LLi_10745 | chr5 | 180668864 | 180668890 | – | INTRON | GNB2L1 | chr5 | 180668639 | 180669173 | – |
| piR_LLi_10747 | chr5 | 180670344 | 180670378 | – | INTRON | GNB2L1 | chr5 | 180669345 | 180670691 | – |
| piR_LLi_7125 | chr3 | 184083101 | 184083129 | + | INTRON | POLR2H | chr3 | 184083014 | 184084504 | + |
| piR_LLi_27812 | chr20 | 2634898 | 2634933 | + | INTRON | NOP56 | chr20 | 2634039 | 2635059 | + |
| piR_LLi_27814 | chr20 | 2637583 | 2637615 | + | INTRON | NOP56 | chr20 | 2637541 | 2637726 | + |
| piR_LLi_27815 | chr20 | 2637624 | 2637658 | + | INTRON | NOP56 | chr20 | 2637541 | 2637726 | + |
| piR_LLi_7316 | chr4 | 2261982 | 2262012 | – | INTRON | MXD4 | chr4 | 2259738 | 2263451 | – |
| piR_LLi_19836 | chr11 | 122929677 | 122929703 | – | INTRON | HSPA8 | chr11 | 122929538 | 122929766 | – |
| piR_LLi_19839 | chr11 | 122930100 | 122930131 | – | INTRON | HSPA8 | chr11 | 122929969 | 122930180 | – |
| piR_LLi_2204 | chr1 | 196885601 | 196885632 | + | INTRON | CFHR4 | chr1 | 196884268 | 196887339 | + |
| piR_LLi_18179 | chr10 | 116239155 | 116239186 | – | INTRON | ABLIM1 | chr10 | 116233715 | 116245055 | – |
| piR_LLi_17912 | chr10 | 95359655 | 95359683 | – | INTRON | RBP4 | chr10 | 95353792 | 95360149 | – |
| piR_LLi_22668 | chr14 | 70235789 | 70235821 | + | INTRON | SRSF5 | chr14 | 70235613 | 70235898 | + |
| piR_LLi_19358 | chr11 | 74907424 | 74907459 | + | INTRON | SLCO2B1 | chr11 | 74904620 | 74907558 | + |
| piR_LLi_4613 | chr2 | 171877221 | 171877256 | – | INTRON | TLK1 | chr2 | 171871458 | 171884848 | – |
| piR_LLi_25984 | chr17 | 65423021 | 65423056 | + | INTRON | PITPNC1 | chr17 | 65374318 | 65528917 | + |
| piR_LLi_27644 | chr19 | 49993872 | 49993906 | + | INTRON | RPL13A | chr19 | 49993833 | 49994035 | + |
| piR_LLi_27190 | chr19 | 12814448 | 12814482 | – | INTRON | TNPO2 | chr19 | 12814340 | 12814527 | – |
| piR_LLi_27192 | chr19 | 13007585 | 13007612 | + | INTRON | GCDH | chr19 | 13007235 | 13007723 | + |
| piR_LLi_5920 | chr3 | 52722933 | 52722967 | + | INTRON | GNL3 | chr3 | 52722208 | 52723089 | + |
| piR_LLi_5921 | chr3 | 52725393 | 52725421 | + | INTRON | GNL3 | chr3 | 52725087 | 52725557 | + |
| piR_LLi_27965 | chr20 | 17943351 | 17943386 | – | INTRON | SNX5 | chr20 | 17937681 | 17949017 | – |
| piR_LLi_22237 | chr14 | 24776482 | 24776513 | – | INTRON | CIDEB | chr14 | 24775729 | 24776576 | – |
| piR_LLi_12717 | chr7 | 33600966 | 33600995 | + | INTRON | BBS9 | chr7 | 33573788 | 33644476 | + |
| piR_LLi_4767 | chr2 | 190440190 | 190440221 | – | INTRON | SLC40A1 | chr2 | 190440046 | 190444538 | – |
| piR_LLi_5264 | chr2 | 236508530 | 236508565 | + | INTRON | AGAP1 | chr2 | 236403493 | 236617822 | + |
| piR_LLi_10379 | chr5 | 148591345 | 148591378 | + | INTRON | ABLIM3 | chr5 | 148590382 | 148596521 | + |
| piR_LLi_14969 | chr8 | 99159770 | 99159797 | + | INTRON | POP1 | chr8 | 99158911 | 99161042 | + |
| piR_LLi_10166 | chr5 | 132892601 | 132892628 | – | INTRON | FSTL4 | chr5 | 132736678 | 132902876 | – |
| piR_LLi_29368 | chr22 | 47050339 | 47050370 | + | INTRON | GRAMD4 | chr22 | 47033857 | 47054083 | + |
| piR_LLi_3157 | chr2 | 29149982 | 29150012 | + | INTRON | WDR43 | chr2 | 29149420 | 29150434 | + |
| piR_LLi_3158 | chr2 | 29150848 | 29150882 | + | INTRON | WDR43 | chr2 | 29150566 | 29152444 | + |
| piR_LLi_3159 | chr2 | 29150873 | 29150899 | + | INTRON | WDR43 | chr2 | 29150566 | 29152444 | + |
| piR_LLi_6469 | chr3 | 114387142 | 114387172 | – | INTRON | ZBTB20 | chr3 | 114219238 | 114412374 | – |
| piR_LLi_4883 | chr2 | 203142838 | 203142871 | + | INTRON | NOP58 | chr2 | 203142725 | 203147073 | + |
| piR_LLi_72 | chr1 | 1166549 | 1166575 | – | INTRON | SDF4 | chr1 | 1164326 | 1167271 | – |
| piR_LLi_24763 | chr16 | 58582503 | 58582538 | – | INTRON | CNOT1 | chr16 | 58581586 | 58583622 | – |
| piR_LLi_72 | chr1 | 1166549 | 1166575 | – | INTRON | SDF4 | chr1 | 1164326 | 1167271 | – |
| piR_LLi_15728 | chr9 | 19063754 | 19063785 | – | INTRON | HAUS6 | chr9 | 19063578 | 19070216 | – |
| piR_LLi_23633 | chr15 | 66639649 | 66639683 | – | INTRON | TIPIN | chr15 | 66633694 | 66641397 | – |
| piR_LLi_24875 | chr16 | 70812939 | 70812970 | – | INTRON | VAC14 | chr16 | 70806075 | 70814692 | – |
| piR_LLi_22088 | chr13 | 111273523 | 111273554 | + | INTRON | CARKD | chr13 | 111268121 | 111274562 | + |
| piR_LLi_17603 | chr10 | 70496928 | 70496955 | + | INTRON | CCAR1 | chr10 | 70496805 | 70497601 | + |
| piR_LLi_17604 | chr10 | 70514961 | 70514996 | + | INTRON | CCAR1 | chr10 | 70514584 | 70515126 | + |
| piR_LLi_16636 | chr9 | 125918876 | 125918903 | – | INTRON | STRBP | chr9 | 125910149 | 125920290 | – |
| piR_LLi_15846 | chr9 | 33933115 | 33933148 | – | INTRON | UBAP2 | chr9 | 33932626 | 33933487 | – |
| piR_LLi_24151 | chr16 | 2737222 | 2737255 | + | INTRON | KCTD5 | chr16 | 2732801 | 2745935 | + |
| piR_LLi_17188 | chr10 | 24813056 | 24813085 | + | INTRON | KIAA1217 | chr10 | 24810858 | 24813251 | + |
| piR_LLi_26268 | chr18 | 3084961 | 3084990 | – | INTRON | MYOM1 | chr18 | 3084025 | 3085042 | – |
| piR_LLi_14703 | chr8 | 68497629 | 68497655 | – | INTRON | CPA6 | chr8 | 68430282 | 68536410 | – |
| piR_LLi_16727 | chr9 | 131844912 | 131844939 | + | INTRON | DOLPP1 | chr9 | 131843486 | 131846946 | + |
| piR_LLi_12035 | chr6 | 142740789 | 142740824 | + | INTRON | ADGRG6 | chr6 | 142738506 | 142740957 | + |
| piR_LLi_7377 | chr4 | 6999028 | 6999054 | + | INTRON | TBC1D14 | chr4 | 6998134 | 7000811 | + |
| piR_LLi_7385 | chr4 | 7584331 | 7584365 | + | INTRON | SORCS2 | chr4 | 7533356 | 7640054 | + |
| piR_LLi_22204 | chr14 | 21860308 | 21860342 | – | INTRON | CHD8 | chr14 | 21860105 | 21860665 | – |
| piR_LLi_22205 | chr14 | 21865450 | 21865484 | – | INTRON | CHD8 | chr14 | 21864051 | 21865981 | – |
| piR_LLi_22182 | chr14 | 20791337 | 20791364 | – | INTRON | CCNB1IP1 | chr14 | 20784719 | 20793697 | – |
| piR_LLi_13491 | chr7 | 111928980 | 111929007 | + | INTRON | ZNF277 | chr7 | 111927129 | 111935923 | + |
| piR_LLi_18303 | chr10 | 126176856 | 126176890 | + | INTRON | LHPP | chr10 | 126172895 | 126176990 | + |
| piR_LLi_808 | chr1 | 45136641 | 45136675 | – | INTRON | TMEM53 | chr1 | 45125967 | 45140002 | – |
| piR_LLi_809 | chr1 | 45136689 | 45136716 | – | INTRON | TMEM53 | chr1 | 45125967 | 45140002 | – |
| piR_LLi_3162 | chr2 | 29355208 | 29355236 | + | INTRON | CLIP4 | chr2 | 29355111 | 29356520 | + |
| piR_LLi_29206 | chr22 | 36891827 | 36891861 | – | INTRON | FOXRED2 | chr22 | 36889850 | 36892013 | – |
| piR_LLi_25715 | chr17 | 41105504 | 41105534 | – | INTRON | PTGES3L–AARSD1 | chr17 | 41103911 | 41105740 | – |
| piR_LLi_12844 | chr7 | 45144051 | 45144082 | – | INTRON | TBRG4 | chr7 | 45143042 | 45145039 | – |
| piR_LLi_14720 | chr8 | 70602297 | 70602328 | – | INTRON | SLCO5A1 | chr8 | 70594578 | 70617265 | – |
| piR_LLi_3659 | chr2 | 75739770 | 75739804 | – | INTRON | EVA1A | chr2 | 75720735 | 75745181 | – |
| piR_LLi_28232 | chr20 | 40196679 | 40196704 | – | INTRON | CHD6 | chr20 | 40179999 | 40246978 | – |
| piR_LLi_3931 | chr2 | 103340480 | 103340513 | – | INTRON | MFSD9 | chr2 | 103340368 | 103343303 | – |
| piR_LLi_16316 | chr9 | 97831454 | 97831488 | + | INTRON | C9orf3 | chr9 | 97823092 | 97842975 | + |
| piR_LLi_20474 | chr12 | 50850355 | 50850390 | + | INTRON | LARP4 | chr12 | 50848200 | 50854917 | + |
| piR_LLi_19264 | chr11 | 69464718 | 69464744 | + | INTRON | CCND1 | chr11 | 69462910 | 69465885 | + |
| piR_LLi_20098 | chr12 | 7052994 | 7053023 | + | 5_UTR | C12orf57 | chr12 | 7052979 | 7053284 | + |
| piR_LLi_27965 | chr20 | 17943351 | 17943386 | – | INTRON | SNX5 | chr20 | 17937681 | 17949017 | – |
| piR_LLi_3492 | chr2 | 61413387 | 61413418 | + | INTRON | AHSA2 | chr2 | 61412753 | 61413580 | + |
| piR_LLi_19836 | chr11 | 122929677 | 122929703 | – | INTRON | HSPA8 | chr11 | 122929538 | 122929766 | – |
| piR_LLi_19839 | chr11 | 122930100 | 122930131 | – | INTRON | HSPA8 | chr11 | 122929969 | 122930180 | – |
| piR_LLi_29876 | chrX | 79955304 | 79955338 | – | INTRON | BRWD3 | chrX | 79952362 | 79955455 | – |
| piR_LLi_1987 | chr1 | 173938453 | 173938481 | – | INTRON | RC3H1 | chr1 | 173934258 | 173939642 | – |
| piR_LLi_26811 | chr18 | 59533152 | 59533181 | – | INTRON | RNF152 | chr18 | 59483831 | 59560027 | – |
| piR_LLi_9801 | chr5 | 93905171 | 93905206 | – | INTRON | KIAA0825 | chr5 | 93872832 | 93918137 | – |
| piR_LLi_16724 | chr9 | 131702507 | 131702536 | + | INTRON | PHYHD1 | chr9 | 131698924 | 131702647 | + |
| piR_LLi_24134 | chr16 | 2054973 | 2055001 | – | INTRON | ZNF598 | chr16 | 2053729 | 2059524 | – |
| piR_LLi_5876 | chr3 | 49282204 | 49282235 | + | INTRON | CCDC36 | chr3 | 49282166 | 49292822 | + |
| piR_LLi_25984 | chr17 | 65423021 | 65423056 | + | INTRON | PITPNC1 | chr17 | 65374318 | 65528917 | + |
| piR_LLi_14165 | chr8 | 16903870 | 16903901 | + | INTRON | MICU3 | chr8 | 16885169 | 16921592 | + |
| piR_LLi_22182 | chr14 | 20791337 | 20791364 | – | INTRON | CCNB1IP1 | chr14 | 20784719 | 20793697 | – |
| piR_LLi_22182 | chr14 | 20791337 | 20791364 | – | INTRON | CCNB1IP1 | chr14 | 20786133 | 20793697 | – |
| piR_LLi_26446 | chr18 | 21407158 | 21407188 | + | INTRON | LAMA3 | chr18 | 21406763 | 21407277 | + |
| piR_LLi_12717 | chr7 | 33600966 | 33600995 | + | INTRON | BBS9 | chr7 | 33573788 | 33644476 | + |
| piR_LLi_12035 | chr6 | 142740789 | 142740824 | + | INTRON | ADGRG6 | chr6 | 142738506 | 142740957 | + |
| piR_LLi_12844 | chr7 | 45144051 | 45144082 | – | INTRON | TBRG4 | chr7 | 45143042 | 45145039 | – |
| piR_LLi_20474 | chr12 | 50850355 | 50850390 | + | INTRON | LARP4 | chr12 | 50848200 | 50854917 | + |
| piR_LLi_10266 | chr5 | 138611867 | 138611894 | + | INTRON | MATR3 | chr5 | 138611839 | 138614015 | + |
| piR_LLi_10267 | chr5 | 138611977 | 138612012 | + | INTRON | MATR3 | chr5 | 138611839 | 138614015 | + |
| piR_LLi_20474 | chr12 | 50850355 | 50850390 | + | INTRON | LARP4 | chr12 | 50848200 | 50854917 | + |
| piR_LLi_21958 | chr13 | 100542424 | 100542456 | + | INTRON | CLYBL | chr13 | 100523316 | 100543571 | + |
| piR_LLi_5920 | chr3 | 52722933 | 52722967 | + | INTRON | GNL3 | chr3 | 52722208 | 52723089 | + |
| piR_LLi_5921 | chr3 | 52725393 | 52725421 | + | INTRON | GNL3 | chr3 | 52725087 | 52725557 | + |
| piR_LLi_5920 | chr3 | 52722933 | 52722967 | + | INTRON | GNL3 | chr3 | 52722208 | 52723089 | + |
| piR_LLi_5921 | chr3 | 52725393 | 52725421 | + | INTRON | GNL3 | chr3 | 52725087 | 52725557 | + |
| piR_LLi_24763 | chr16 | 58582503 | 58582538 | – | INTRON | CNOT1 | chr16 | 58581586 | 58583622 | – |
| piR_LLi_16880 | chr9 | 139908198 | 139908228 | – | INTRON | ABCA2 | chr9 | 139908015 | 139908283 | – |
| piR_LLi_1974 | chr1 | 173833322 | 173833357 | – | INTRON | GAS5 | chr1 | 173833213 | 173833394 | – |
| piR_LLi_1975 | chr1 | 173833505 | 173833540 | – | INTRON | GAS5 | chr1 | 173833442 | 173833621 | – |
| piR_LLi_1976 | chr1 | 173833550 | 173833584 | – | INTRON | GAS5 | chr1 | 173833442 | 173833621 | – |
| piR_LLi_1977 | chr1 | 173834007 | 173834042 | – | INTRON | GAS5 | chr1 | 173833842 | 173834366 | – |
| piR_LLi_1982 | chr1 | 173836015 | 173836049 | – | INTRON | GAS5 | chr1 | 173835934 | 173836128 | – |
| piR_LLi_1983 | chr1 | 173836045 | 173836077 | – | INTRON | GAS5 | chr1 | 173835934 | 173836128 | – |
| piR_LLi_589 | chr1 | 28834048 | 28834083 | + | INTRON | SNHG3 | chr1 | 28832596 | 28834639 | + |
| piR_LLi_590 | chr1 | 28835068 | 28835103 | + | INTRON | SNHG3 | chr1 | 28834672 | 28835341 | + |
| piR_LLi_592 | chr1 | 28835245 | 28835274 | + | INTRON | SNHG3 | chr1 | 28834672 | 28835341 | + |
| piR_LLi_11596 | chr6 | 86387304 | 86387339 | – | INTRON | SNHG5 | chr6 | 86387210 | 86387512 | – |
| piR_LLi_10266 | chr5 | 138611867 | 138611894 | + | INTRON | SNHG4 | chr5 | 138611839 | 138614015 | + |
| piR_LLi_10267 | chr5 | 138611977 | 138612012 | + | INTRON | SNHG4 | chr5 | 138611839 | 138614015 | + |
| piR_LLi_28197 | chr20 | 37078114 | 37078149 | + | INTRON | SNHG11 | chr20 | 37077373 | 37078978 | + |
| piR_LLi_8338 | chr4 | 119200343 | 119200378 | + | INTRON | SNHG8 | chr4 | 119200292 | 119200543 | + |
| piR_LLi_9954 | chr5 | 111497179 | 111497213 | + | INTRON | EPB41L4A–AS1 | chr5 | 111496992 | 111497399 | + |
| piR_LLi_14784 | chr8 | 77557821 | 77557849 | – | INTRON | ZFHX4–AS1 | chr8 | 77528686 | 77585061 | – |
| piR_LLi_27644 | chr19 | 49993872 | 49993906 | + | INTRON | RPL13AP5 | chr19 | 49993833 | 49994035 | + |
| piR_LLi_25374 | chr17 | 16342817 | 16342851 | + | INTRON | LRRC75A–AS1 | chr17 | 16342728 | 16343498 | + |
| piR_LLi_25376 | chr17 | 16344537 | 16344568 | + | INTRON | LRRC75A–AS1 | chr17 | 16343567 | 16344681 | + |
| piR_LLi_25374 | chr17 | 16342817 | 16342851 | + | INTRON | LRRC75A–AS1 | chr17 | 16342728 | 16343498 | + |
| piR_LLi_25376 | chr17 | 16344537 | 16344568 | + | INTRON | LRRC75A–AS1 | chr17 | 16344444 | 16344681 | + |
| piR_LLi_25374 | chr17 | 16342817 | 16342851 | + | INTRON | LRRC75A–AS1 | chr17 | 16342728 | 16342894 | + |
| piR_LLi_25376 | chr17 | 16344537 | 16344568 | + | INTRON | LRRC75A–AS1 | chr17 | 16344444 | 16344681 | + |
| piR_LLi_25376 | chr17 | 16344537 | 16344568 | + | INTRON | LRRC75A–AS1 | chr17 | 16343567 | 16344681 | + |
| piR_LLi_25376 | chr17 | 16344537 | 16344568 | + | INTRON | LRRC75A–AS1 | chr17 | 16344444 | 16344681 | + |
| piR_LLi_25376 | chr17 | 16344537 | 16344568 | + | INTRON | LRRC75A–AS1 | chr17 | 16344444 | 16344681 | + |
| piR_LLi_25374 | chr17 | 16342817 | 16342851 | + | INTRON | LRRC75A–AS1 | chr17 | 16342728 | 16342894 | + |
| piR_LLi_25376 | chr17 | 16344537 | 16344568 | + | INTRON | LRRC75A–AS1 | chr17 | 16344444 | 16344681 | + |
| piR_LLi_25374 | chr17 | 16342817 | 16342851 | + | INTRON | LRRC75A–AS1 | chr17 | 16342728 | 16342973 | + |
| piR_LLi_25376 | chr17 | 16344537 | 16344568 | + | INTRON | LRRC75A–AS1 | chr17 | 16343567 | 16344681 | + |
| piR_LLi_25374 | chr17 | 16342817 | 16342851 | + | INTRON | LRRC75A–AS1 | chr17 | 16342728 | 16343498 | + |
| piR_LLi_25376 | chr17 | 16344537 | 16344568 | + | INTRON | LRRC75A–AS1 | chr17 | 16344444 | 16344681 | + |
| piR_LLi_25374 | chr17 | 16342817 | 16342851 | + | INTRON | LRRC75A–AS1 | chr17 | 16342728 | 16342894 | + |
| piR_LLi_25376 | chr17 | 16344537 | 16344568 | + | INTRON | LRRC75A–AS1 | chr17 | 16343567 | 16344681 | + |
| piR_LLi_25374 | chr17 | 16342817 | 16342851 | + | INTRON | LRRC75A–AS1 | chr17 | 16342728 | 16342894 | + |
| piR_LLi_25376 | chr17 | 16344537 | 16344568 | + | INTRON | LRRC75A–AS1 | chr17 | 16344444 | 16344681 | + |
| piR_LLi_25376 | chr17 | 16344537 | 16344568 | + | INTRON | LRRC75A–AS1 | chr17 | 16343567 | 16344681 | + |
| piR_LLi_25374 | chr17 | 16342817 | 16342851 | + | INTRON | LRRC75A–AS1 | chr17 | 16342728 | 16342973 | + |
| piR_LLi_25376 | chr17 | 16344537 | 16344568 | + | INTRON | LRRC75A–AS1 | chr17 | 16344444 | 16344681 | + |
| piR_LLi_25374 | chr17 | 16342817 | 16342851 | + | INTRON | LRRC75A–AS1 | chr17 | 16342728 | 16343498 | + |
| piR_LLi_25376 | chr17 | 16344537 | 16344568 | + | INTRON | LRRC75A–AS1 | chr17 | 16344444 | 16373615 | + |
| piR_LLi_25374 | chr17 | 16342817 | 16342851 | + | INTRON | LRRC75A–AS1 | chr17 | 16342728 | 16343498 | + |
| piR_LLi_25376 | chr17 | 16344537 | 16344568 | + | INTRON | LRRC75A–AS1 | chr17 | 16344444 | 16373615 | + |
| piR_LLi_25374 | chr17 | 16342817 | 16342851 | + | INTRON | LRRC75A–AS1 | chr17 | 16342728 | 16343498 | + |
| piR_LLi_25376 | chr17 | 16344537 | 16344568 | + | INTRON | LRRC75A–AS1 | chr17 | 16344444 | 16366146 | + |
| piR_LLi_25374 | chr17 | 16342817 | 16342851 | + | INTRON | LRRC75A–AS1 | chr17 | 16342728 | 16343498 | + |
| piR_LLi_25376 | chr17 | 16344537 | 16344568 | + | INTRON | LRRC75A–AS1 | chr17 | 16344444 | 16366146 | + |
| piR_LLi_25374 | chr17 | 16342817 | 16342851 | + | INTRON | LRRC75A–AS1 | chr17 | 16342728 | 16343498 | + |
| piR_LLi_25376 | chr17 | 16344537 | 16344568 | + | INTRON | LRRC75A–AS1 | chr17 | 16344444 | 16373615 | + |
| piR_LLi_25374 | chr17 | 16342817 | 16342851 | + | INTRON | LRRC75A–AS1 | chr17 | 16342728 | 16343498 | + |
| piR_LLi_25376 | chr17 | 16344537 | 16344568 | + | INTRON | LRRC75A–AS1 | chr17 | 16343567 | 16364206 | + |
| piR_LLi_25374 | chr17 | 16342817 | 16342851 | + | INTRON | LRRC75A–AS1 | chr17 | 16342728 | 16343498 | + |
| piR_LLi_25376 | chr17 | 16344537 | 16344568 | + | INTRON | LRRC75A–AS1 | chr17 | 16344444 | 16366146 | + |
| piR_LLi_25374 | chr17 | 16342817 | 16342851 | + | INTRON | LRRC75A–AS1 | chr17 | 16342728 | 16343498 | + |
| piR_LLi_25376 | chr17 | 16344537 | 16344568 | + | INTRON | LRRC75A–AS1 | chr17 | 16344444 | 16366146 | + |
| piR_LLi_25374 | chr17 | 16342817 | 16342851 | + | INTRON | LRRC75A–AS1 | chr17 | 16342728 | 16343498 | + |
| piR_LLi_25374 | chr17 | 16342817 | 16342851 | + | INTRON | LRRC75A–AS1 | chr17 | 16342728 | 16342973 | + |
| piR_LLi_25376 | chr17 | 16344537 | 16344568 | + | INTRON | LRRC75A–AS1 | chr17 | 16344444 | 16344681 | + |
| piR_LLi_27812 | chr20 | 2634898 | 2634933 | + | INTRON | NOP56 | chr20 | 2634039 | 2635059 | + |
| piR_LLi_27814 | chr20 | 2637583 | 2637615 | + | INTRON | NOP56 | chr20 | 2637541 | 2637726 | + |
| piR_LLi_27815 | chr20 | 2637624 | 2637658 | + | INTRON | NOP56 | chr20 | 2637541 | 2637726 | + |
| piR_LLi_13847 | chr7 | 149286161 | 149286190 | – | INTRON | ZNF767P | chr7 | 149255930 | 149317014 | – |
| piR_LLi_13847 | chr7 | 149286161 | 149286190 | – | INTRON | ZNF767P | chr7 | 149250107 | 149317014 | – |
| piR_LLi_30117 | chrX | 135961356 | 135961389 | – | INTRON | RBMX | chrX | 135961282 | 135961477 | – |
| piR_LLi_30118 | chrX | 135961401 | 135961432 | – | INTRON | RBMX | chrX | 135961282 | 135961477 | – |
| piR_LLi_30117 | chrX | 135961356 | 135961389 | – | INTRON | RBMX | chrX | 135961317 | 135961477 | – |
| piR_LLi_30118 | chrX | 135961401 | 135961432 | – | INTRON | RBMX | chrX | 135961317 | 135961477 | – |
| piR_LLi_589 | chr1 | 28834048 | 28834083 | + | INTRON | RCC1 | chr1 | 28832596 | 28834639 | + |
| piR_LLi_590 | chr1 | 28835068 | 28835103 | + | INTRON | RCC1 | chr1 | 28834672 | 28835341 | + |
| piR_LLi_592 | chr1 | 28835245 | 28835274 | + | INTRON | RCC1 | chr1 | 28834672 | 28835341 | + |
| piR_LLi_589 | chr1 | 28834048 | 28834083 | + | INTRON | RCC1 | chr1 | 28832596 | 28834639 | + |
| piR_LLi_590 | chr1 | 28835068 | 28835103 | + | INTRON | RCC1 | chr1 | 28834672 | 28835341 | + |
| piR_LLi_592 | chr1 | 28835245 | 28835274 | + | INTRON | RCC1 | chr1 | 28834672 | 28835341 | + |
| piR_LLi_20474 | chr12 | 50850355 | 50850390 | + | INTRON | LARP4 | chr12 | 50848200 | 50854917 | + |
| piR_LLi_16636 | chr9 | 125918876 | 125918903 | – | INTRON | STRBP | chr9 | 125910149 | 125920290 | – |
| piR_LLi_8338 | chr4 | 119200343 | 119200378 | + | INTRON | SNHG8 | chr4 | 119200292 | 119200543 | + |
| piR_LLi_8338 | chr4 | 119200343 | 119200378 | + | INTRON | SNHG8 | chr4 | 119199947 | 119200543 | + |
| piR_LLi_589 | chr1 | 28834048 | 28834083 | + | INTRON | SNHG3 | chr1 | 28832839 | 28834639 | + |
| piR_LLi_590 | chr1 | 28835068 | 28835103 | + | INTRON | SNHG3 | chr1 | 28834672 | 28835341 | + |
| piR_LLi_592 | chr1 | 28835245 | 28835274 | + | INTRON | SNHG3 | chr1 | 28834672 | 28835341 | + |
| piR_LLi_10266 | chr5 | 138611867 | 138611894 | + | INTRON | SNHG4 | chr5 | 138611839 | 138614015 | + |
| piR_LLi_10267 | chr5 | 138611977 | 138612012 | + | INTRON | SNHG4 | chr5 | 138611839 | 138614015 | + |
| piR_LLi_27753 | chr19 | 56906736 | 56906767 | + | INTRON | ZNF582–AS1 | chr19 | 56905810 | 56909249 | + |
| piR_LLi_27753 | chr19 | 56906736 | 56906767 | + | INTRON | ZNF582–AS1 | chr19 | 56905422 | 56909249 | + |
| piR_LLi_27753 | chr19 | 56906736 | 56906767 | + | INTRON | ZNF582–AS1 | chr19 | 56905810 | 56909249 | + |
| piR_LLi_7111 | chr3 | 183169640 | 183169674 | + | INTRON | LINC00888 | chr3 | 183165735 | 183169865 | + |
| piR_LLi_7111 | chr3 | 183169640 | 183169674 | + | INTRON | LINC00888 | chr3 | 183165553 | 183169865 | + |
| piR_LLi_22088 | chr13 | 111273523 | 111273554 | + | INTRON | CARKD | chr13 | 111267994 | 111274562 | + |
| piR_LLi_22088 | chr13 | 111273523 | 111273554 | + | INTRON | CARKD | chr13 | 111267994 | 111274562 | + |
| piR_LLi_25374 | chr17 | 16342817 | 16342851 | + | INTRON | LRRC75A–AS1 | chr17 | 16342707 | 16343498 | + |
| piR_LLi_25376 | chr17 | 16344537 | 16344568 | + | INTRON | LRRC75A–AS1 | chr17 | 16344444 | 16344681 | + |
| piR_LLi_25374 | chr17 | 16342817 | 16342851 | + | INTRON | LRRC75A–AS1 | chr17 | 16342728 | 16342973 | + |
| piR_LLi_25376 | chr17 | 16344537 | 16344568 | + | INTRON | LRRC75A–AS1 | chr17 | 16344444 | 16344681 | + |
| piR_LLi_25374 | chr17 | 16342817 | 16342851 | + | INTRON | LRRC75A–AS1 | chr17 | 16342728 | 16343498 | + |
| piR_LLi_25376 | chr17 | 16344537 | 16344568 | + | INTRON | LRRC75A–AS1 | chr17 | 16343567 | 16373615 | + |
| piR_LLi_25374 | chr17 | 16342817 | 16342851 | + | INTRON | LRRC75A–AS1 | chr17 | 16342728 | 16343498 | + |
| piR_LLi_25374 | chr17 | 16342817 | 16342851 | + | INTRON | LRRC75A–AS1 | chr17 | 16342374 | 16343498 | + |
| piR_LLi_25376 | chr17 | 16344537 | 16344568 | + | INTRON | LRRC75A–AS1 | chr17 | 16344444 | 16373615 | + |
| piR_LLi_25374 | chr17 | 16342817 | 16342851 | + | INTRON | LRRC75A–AS1 | chr17 | 16342728 | 16343498 | + |
| piR_LLi_25376 | chr17 | 16344537 | 16344568 | + | INTRON | LRRC75A–AS1 | chr17 | 16343567 | 16344681 | + |
| piR_LLi_25374 | chr17 | 16342817 | 16342851 | + | INTRON | LRRC75A–AS1 | chr17 | 16342728 | 16342973 | + |
| piR_LLi_25376 | chr17 | 16344537 | 16344568 | + | INTRON | LRRC75A–AS1 | chr17 | 16343567 | 16344681 | + |
| piR_LLi_25374 | chr17 | 16342817 | 16342851 | + | INTRON | LRRC75A–AS1 | chr17 | 16342728 | 16343424 | + |
| piR_LLi_25376 | chr17 | 16344537 | 16344568 | + | INTRON | LRRC75A–AS1 | chr17 | 16344444 | 16344681 | + |
| piR_LLi_24763 | chr16 | 58582503 | 58582538 | – | INTRON | CNOT1 | chr16 | 58581586 | 58583622 | – |
| piR_LLi_16727 | chr9 | 131844912 | 131844939 | + | INTRON | DOLPP1 | chr9 | 131843486 | 131846946 | + |
| piR_LLi_27644 | chr19 | 49993872 | 49993906 | + | INTRON | RPL13A | chr19 | 49993833 | 49994035 | + |
| piR_LLi_1152 | chr1 | 76252755 | 76252790 | + | INTRON | RABGGTB | chr1 | 76251959 | 76253181 | + |
| piR_LLi_27192 | chr19 | 13007585 | 13007612 | + | INTRON | GCDH | chr19 | 13007235 | 13007723 | + |
| piR_LLi_27192 | chr19 | 13007585 | 13007612 | + | INTRON | GCDH | chr19 | 13007235 | 13007723 | + |
| piR_LLi_17603 | chr10 | 70496928 | 70496955 | + | INTRON | CCAR1 | chr10 | 70496805 | 70497601 | + |
| piR_LLi_17604 | chr10 | 70514961 | 70514996 | + | INTRON | CCAR1 | chr10 | 70514584 | 70515126 | + |
| piR_LLi_21958 | chr13 | 100542424 | 100542456 | + | INTRON | CLYBL | chr13 | 100523316 | 100543571 | + |
| piR_LLi_3162 | chr2 | 29355208 | 29355236 | + | INTRON | CLIP4 | chr2 | 29354263 | 29356520 | + |
| piR_LLi_25661 | chr17 | 37881865 | 37881894 | + | INTRON | ERBB2 | chr17 | 37881655 | 37881959 | + |
| piR_LLi_26064 | chr17 | 73030044 | 73030074 | + | INTRON | KCTD2 | chr17 | 73028819 | 73030127 | + |
| piR_LLi_9693 | chr5 | 79946906 | 79946935 | – | INTRON | DHFR | chr5 | 79945313 | 79950222 | – |
| piR_LLi_9694 | chr5 | 79946933 | 79946965 | – | INTRON | DHFR | chr5 | 79945313 | 79950222 | – |
| piR_LLi_9699 | chr5 | 79947460 | 79947495 | – | INTRON | DHFR | chr5 | 79945313 | 79950222 | – |
| piR_LLi_6469 | chr3 | 114387142 | 114387172 | – | INTRON | ZBTB20 | chr3 | 114219238 | 114519947 | – |
| piR_LLi_752 | chr1 | 41465807 | 41465838 | + | INTRON | CTPS1 | chr1 | 41463198 | 41466700 | + |
| piR_LLi_20098 | chr12 | 7052994 | 7053023 | + | 5_UTR | C12orf57 | chr12 | 7052979 | 7053336 | + |
