# Supplementary material for: Specific patterns of PIWI-interacting small noncoding RNA expression in dysplastic liver nodules and hepatocellular carcinoma
Source: Oncotarget. 2016 Jul 13;7(34):54650–61. doi: 10.18632/oncotarget.10567 (PMC5342370; doi:10.18632/oncotarget.10567)
Supplement: Supplementary file 7 [file oncotarget-07-54650-s007.docx]

| Supplementary Table S7: piRNA–like differential expression analysis between cirrhosis and HCC tissues | | | |
| --- | --- | --- | --- |
| **piRNA ID** | ***p*Value** | **FDR** | **FC** |
| piR_LLi_11071 | 0.00245 | 0.02047 | –16.15 |
| piR_LLi_3740 | 0.00294 | 0.02291 | –5.09 |
| piR_LLi_11084 | 0.00669 | 0.03431 | 2.86 |
| piR_LLi_30564 | 0.00100 | 0.01568 | 2.86 |
| piR_LLi_1970 | 0.00696 | 0.03472 | 3.22 |
| piR_LLi_15689 | 0.00251 | 0.02047 | 4.16 |
| piR_LLi_11041 | 0.00598 | 0.03203 | 4.17 |
| piR_LLi_16037 | 0.00024 | 0.00870 | 5.30 |
| piR_LLi_11958 | 0.00669 | 0.03431 | 5.47 |
| piR_LLi_30579 | 0.00251 | 0.02047 | 5.71 |
| piR_LLi_5920 | 0.00433 | 0.02704 | 6.42 |
| piR_LLi_589 | 0.00153 | 0.01777 | 7.93 |
| piR_LLi_17146 | 0.00134 | 0.01657 | 8.37 |
| piR_LLi_20856 | 0.00241 | 0.02047 | 9.39 |
| piR_LLi_3762 | 0.00042 | 0.00888 | 11.72 |
| piR_LLi_11072 | 0.00251 | 0.02047 | 11.81 |
| piR_LLi_30732 | 0.00059 | 0.01017 | 13.49 |
| piR_LLi_28226 | 0.00015 | 0.00785 | 26.08 |
| piR_LLi_28783 | 0.00235 | 0.02047 | 44.14 |
| piR_LLi_43 | 0.00235 | 0.02047 | 50.07 |
| piR_LLi_7111 | 0.00235 | 0.02047 | 50.54 |
| piR_LLi_2776 | 0.00235 | 0.02047 | 56.75 |
| piR_LLi_30552 | 0.00119 | 0.01639 | 79.97 |
| piR_LLi_24573 | 0.00119 | 0.01639 | 95.90 |
| piR_LLi_25890 | 0.00509 | 0.02857 | 133.25 |
| piR_LLi_22437 | 0.00119 | 0.01639 | 133.47 |
| piR_LLi_2292 | 0.00476 | 0.02801 | 135.82 |
| piR_LLi_13838 | 0.00349 | 0.02614 | 161.39 |
| piR_LLi_10266 | 0.00165 | 0.01853 | 162.86 |
| piR_LLi_25502 | 0.00556 | 0.03026 | 163.33 |
| piR_LLi_27963 | 0.00183 | 0.01991 | 187.86 |
| piR_LLi_590 | 0.00051 | 0.00966 | 189.35 |
| piR_LLi_24259 | 0.00029 | 0.00872 | 199.52 |
| piR_LLi_8643 | 0.00349 | 0.02614 | 199.60 |
| piR_LLi_13330 | 0.00708 | 0.03483 | 200.49 |
| piR_LLi_18303 | 0.00396 | 0.02704 | 208.30 |
| piR_LLi_10267 | 0.00092 | 0.01509 | 220.67 |
| piR_LLi_9589 | 0.00058 | 0.01017 | 223.28 |
| piR_LLi_17148 | 0.00235 | 0.02047 | 240.50 |
| piR_LLi_18697 | 0.00633 | 0.03340 | 254.39 |
| piR_LLi_11094 | 0.00484 | 0.02801 | 264.64 |
| piR_LLi_13788 | 0.00556 | 0.03026 | 275.08 |
| piR_LLi_14969 | 0.00497 | 0.02835 | 342.65 |
| piR_LLi_28197 | 0.00045 | 0.00888 | 349.54 |
| piR_LLi_7 | 0.00009 | 0.00640 | 352.56 |
| piR_LLi_13839 | 0.00128 | 0.01642 | 362.11 |
| piR_LLi_13629 | 0.00756 | 0.03666 | 370.70 |
| piR_LLi_12844 | 0.00009 | 0.00640 | 383.30 |
| piR_LLi_4699 | 0.00941 | 0.04274 | 387.53 |
| piR_LLi_29854 | 0.00127 | 0.01642 | 388.71 |
| piR_LLi_14590 | 0.00008 | 0.00640 | 402.14 |
| piR_LLi_18601 | 0.00012 | 0.00729 | 412.33 |
| piR_LLi_22184 | 0.00039 | 0.00888 | 413.95 |
| piR_LLi_30580 | 0.00682 | 0.03451 | 425.61 |
| piR_LLi_29876 | 0.00953 | 0.04274 | 461.45 |
| piR_LLi_23766 | 0.00007 | 0.00640 | 469.65 |
| piR_LLi_30517 | 0.00020 | 0.00870 | 477.64 |
| piR_LLi_27190 | 0.00042 | 0.00888 | 517.64 |
| piR_LLi_9693 | 0.00036 | 0.00888 | 549.66 |
| piR_LLi_13491 | 0.00025 | 0.00870 | 564.36 |
| piR_LLi_24763 | 0.00279 | 0.02225 | 605.13 |
| piR_LLi_29425 | 0.00375 | 0.02704 | 606.83 |
| piR_LLi_5762 | 0.00032 | 0.00888 | 720.28 |
| piR_LLi_54 | 0.00027 | 0.00870 | 848.35 |
| piR_LLi_11378 | 0.00196 | 0.02047 | 967.04 |
| piR_LLi_6296 | 0.00153 | 0.01777 | 1290.13 |
| piR_LLi_1977 | 0.00008 | 0.00640 | 4666.27 |
| The 67 piRNA–Like liver differential expressed between cirrhosis and HCC tissues, for each piRNA is reported p Value, FDR and FC (in green FC ≤–1.5 and red FC ≥ 1.5). | | | |
