# Supplementary material for: Specific patterns of PIWI-interacting small noncoding RNA expression in dysplastic liver nodules and hepatocellular carcinoma
Source: Oncotarget. 2016 Jul 13;7(34):54650–61. doi: 10.18632/oncotarget.10567 (PMC5342370; doi:10.18632/oncotarget.10567)
Supplement: Supplementary file 8 [file oncotarget-07-54650-s008.docx]

| Supplementary Table S8: Top canonical pathways predicted to be target of piRNAs deregulated in HCC | | | |
| --- | --- | --- | --- |
| **Canonical Pathway** | **Ratio** | **#Molecules** | **piRNA–targeted pathway components** |
| Aryl Hydrocarbon Receptor Signaling | 0,11 | 20 | JUN, CDK4, AHRR, GSTM3, ALDH6A1, RXRG, TP53, ALDH9A1, HSPB7, ALDH1L2, ALDH5A1, MDM2, RBL1, CDK6, MGST3 |
| AMPK Signaling | 0,17 | 15 | PFKFB2, PRKAA1, CPT1A, PPM1L, CHRNA3, CHRNB1, PPP2R3A, AKT2, PFKFB1, EEF2K, AK3, FOXG1, SLC2A4, MAPK13, NOS3, ELAVL1, CAMKK2, PPP2R1B, FOXO3, CHRNA9 |
| Apoptosis Signaling | 0,11 | 15 | CASP10, TNFRSF1B, DFFA, CASP9, CYCS, TP53, CASP6, NAIP, NFKBIB, XIAP, DFFB, AIFM1, CASP2, CASP8, DIABLO |
| ATM Signaling | 0,14 | 8 | JUN, BLM, FANCD2, CBX5, MAPK13, MDM2, TP53, MDM4 |
| Autophagy | 0,11 | 4 | STX17, ATG10, VPS41, ATG12 |
| CDK5 Signaling | 0,11 | 11 | PPP1R10, ITGA2, PPM1L, MAPK13, ADCY2, NTRK2, PPP2R3A, PPP2R1B, ADCY8, GNAL, ADCY10 |
| Cell Cycle Regulation by BTG Family Proteins | 0,20 | 7 | CDK4, E2F2, CCRN4L, PPM1L, PPP2R3A, PPP2R1B, CNOT7 |
| Cell Cycle: G1/S Checkpoint Regulation | 0,14 | 9 | HDAC2, CDK4, E2F2, MDM2, RBL1, CDK6, HDAC9, TP53, HDAC7 |
| Cyclins and Cell Cycle Regulation | 0,13 | 10 | HDAC2, CDK4, E2F2, PPM1L, PPP2R3A, CDK6, PPP2R1B, HDAC9, TP53, HDAC7 |
| Death Receptor Signaling | 0,21 | 19 | PARP12, CASP10, TNFRSF1B, DFFA, CASP9, CYCS, TNFRSF10B, CASP6, NAIP, CFLAR, TNFRSF10A, ZC3HAV1, HSPB7, NFKBIB, XIAP, DFFB, CASP2, CASP8, DIABLO |
| DNA damage–induced 14–3–3σ Signaling | 0,21 | 4 | TP53, AKT2, RAD1, HUS1 |
| DNA Double–Strand Break Repair by Non–Homologous End Joining | 0,14 | 2 | XRCC5, DCLRE1C |
| DNA Methylation and Transcriptional Repression Signaling | 0,15 | 3 | HDAC2, SAP30, RBBP4 |
| GADD45 Signaling | 0,11 | 2 | CDK4, TP53 |
| Hepatic Cholestasis | 0,10 | 16 | ABCB1, JUN, TNFRSF1B, NR1H4, PPARA, ATP8B1, ADCY10, IRAK4, ABCB11, HSD3B7, IL18, IL11, NFKBIB, IL17C, ADCY2, ADCY8 |
| HIPPO signaling | 0,17 | 14 | LATS1, AMOT, NF2, PPM1L, PPP2R3A, WWC1, PPP1R10, SMAD5, ITCH, SAV1, PPP2R1B, YWHAB, DLG2, STK4 |
| HMGB1 Signaling | 0,09 | 11 | JUN, MAP2K5, IL18, CDC42, IL11, HMGB1, TNFRSF1B, MAPK13, IL17C, SERPINE1, AKT2 |
| Myc Mediated Apoptosis Signaling | 0,10 | 6 | CASP9, CYCS, TP53, AKT2, YWHAB, CASP8 |
| p53 Signaling | 0,14 | 14 | JUN, CDK4, BIRC5, TP53, AKT2, TNFRSF10B, CASP6, TNFRSF10A, TRIM29, PLAGL1, MDM2, HDAC9, TIGAR, MDM4 |
| PI3K/AKT Signaling | 0,13 | 16 | GDF15, INPP5F, ITGA2, PPM1L, JAK3, PPP2R3A, TP53, AKT2, NFKBIB, MDM2, NOS3, EIF4E, LIMS1, PPP2R1B, YWHAB, FOXO3 |
| Protein Ubiquitination Pathway | 0,11 | 29 | USP19, USP29, UBR2, DNAJC16, USP11, CBL, HSPA14, DNAJC21, HSPB7, XIAP, PSMB2, PSMD5, USP4, PSMA5, DNAJC30, PARK2, DNAJC10, USP3, UBE2G2, DNAJC14, VHL, DNAJC3, USP15, USP13, USP49, UBE2C, MDM2, PAN2, NEDD4L |
| PTEN Signaling | 0,09 | 11 | FOXG1, CDC42, INPP5F, ITGA2, CASP9, BCAR1, NTRK2, TNFRSF11A, CBL, AKT2, FOXO3 |
| PXR/RXR Activation | 0,13 | 8 | ABCB1, ABCB11, CPT1A, PPARA, G6PC, CES2, AKT2, FOXO3 |
| Retinoic acid Mediated Apoptosis Signaling | 0,19 | 11 | PARP12, CFLAR, TNFRSF10A, ZC3HAV1, CASP9, RXRG, CYCS, DAP3, IFNAR1, CASP8, TNFRSF10B |
| Role of CHK Proteins in Cell Cycle Checkpoint Control | 0,15 | 8 | E2F2, PPM1L, CLSPN, PPP2R3A, PPP2R1B, TP53, RAD1, HUS1 |
| Telomerase Signaling | 0,14 | 13 | HDAC2, TEP1, PPM1L, IL2RA, PPP2R3A, TP53, AKT2, HDAC7, EGF, ELF5, TERF1, PPP2R1B, HDAC9 |
| TNFR1 Signaling | 0,21 | 10 | JUN, NAIP, CDC42, NFKBIB, CASP9, XIAP, CYCS, CASP2, CASP8, CASP6 |
| TNFR2 Signaling | 0,18 | 5 | JUN, NAIP, TNFRSF1B, NFKBIB, XIAP |
| Tumoricidal Function of Hepatic Natural Killer Cells | 0,33 | 8 | ITGAL, CASP9, DFFA, DFFB, AIFM1, CYCS, CASP8, CASP6 |
| TWEAK Signaling | 0,21 | 7 | NAIP, NFKBIB, CASP9, XIAP, CYCS, CASP8, CASP6 |
| Wnt/β–catenin Signaling | 0,10 | 17 | JUN, CDH2, SOX6, PPM1L, PPP2R3A, CDH3, TP53, AKT2, APPL1, KREMEN1, LRP1, FZD3, MDM2, CDH1, PPP2R1B, CSNK1G1, DVL3 |
| Xenobiotic Metabolism Signaling | 0,10 | 25 | ABCB1, MAP2K5, PPP2R3A, SUMO1, MAP3K13, ALDH9A1, SULT1A1, ALDH1L2, PPP2R1B, MGST3, CHST3, AHRR, PPM1L, ALDH6A1, GSTM3, CAMK1D, CES1, MAP3K9, SULT1C2, CAMK2G, MAPK13, ALDH5A1, CES2, MAP3K15, CHST5 |
| Ingenuity Pathway Analysis was used to identify canonical pathways associated with piRNA targets | | | |
